# A deep learning model captures position-specific effects of plant regulatory sequences and suggests genes under complex regulation

**DOI:** 10.1093/plphys/kiag473

**Published:** 2026-07-07

**Authors:** Kevin C Rockenbach, Silvia F Zanini, Alison C Tidy, Richard J Morris, Rachel Wells, Agnieszka A Golicz

**Affiliations:** Department of Agrobioinformatics, Justus-Liebig-University, Heinrich-Buff-Ring 38, 35392 Giessen, Germany; Department of Agrobioinformatics, Justus-Liebig-University, Heinrich-Buff-Ring 38, 35392 Giessen, Germany; Division of Plant and Crop Sciences, School of Biosciences, University of Nottingham, Sutton Bonington Campus, Sutton Bonington, LE12 5RD, Leicestershire, United Kingdom; Department of Computational and Systems Biology, John Innes Centre, Norwich Research Park, Colney Lane, Norwich, NR4 7UH, Norfolk, United Kingdom; Department of Crop Genetics, John Innes Centre, Norwich Research Park, Colney Lane, Norwich, NR4 7UH, Norfolk, United Kingdom; Department of Agrobioinformatics, Justus-Liebig-University, Heinrich-Buff-Ring 38, 35392 Giessen, Germany; Plant Breeding, Wageningen University & Research, Droevendaalsesteeg 1, Building 107, 6708 PB Wageningen, The Netherlands

## Abstract

Deep neural networks can be trained to predict gene expression directly from genomic sequence, thereby implicitly learning regulatory sequence patterns from scratch, minimizing the bias imposed by prior assumptions. A challenging, yet promising prospect is the extraction of novel insights into gene-regulatory mechanisms by probing and interpreting such gene expression models. Using a branched convolutional neural network architecture trained on promoter and terminator sequences, we predict gene expression for allopolyploid *Brassica napus* and the closely related model organism *Arabidopsis thaliana*. We validate the model by comparing predicted and measured expression across ecotypes. We also show that deep learning models can successfully capture the positional binding preferences of some transcription factor families without having been trained on transcription factor binding data. Furthermore, we show that our model did not only detect local sequence patterns but was also able to determine their function based on their positional context. We also found that increased prediction error correlated with additional more distal or epigenetic regulatory input. Our results demonstrate that deep learning can be used to understand the regulatory architecture of gene expression in plants. A better understanding of gene regulation in the context of polyploid genomes is of particular economic importance due to their prevalence among major crops. In the future, we hope that such models may facilitate the targeted engineering of gene regulation in crops.

## Background

Although complete genome assemblies of eukaryotic organisms have been available since the turn of the millennium (Goffeau et al. 1996; C. elegans Sequencing Consortium* 1998; Arabidopsis Genome Initiative 2000), deciphering the grammar by which regulatory information is encoded in the genome remains challenging (Duttke et al. 2024). At the level of transcriptional regulation, transcription factors (TFs) are known to play an important role in facilitating the assembly of the transcriptional machinery (Wingender 1997). TFs are DNA-binding proteins that can selectively bind to cis-regulatory elements (CREs) in the genome based on sequence- and shape-dependent physical interactions with DNA, as well as interactions with other TFs and epigenetic factors (Suzuki and Yagi 1994; Garvie and Wolberger 2001; O’Malley et al. 2016; Samee et al. 2019). Genetic variations in CREs that lead to differences in the ability of TFs to bind have been shown to explain most of the heritable phenotypic variation in maize (Engelhorn et al. 2024). CREs tend to be clustered into cis-regulatory modules (CRMs), such as promoters, enhancers, silencers, and insulators, but the underlying grammar of the constituent CREs, meaning the set of rules governing their relative positioning and orientation, is not well understood (Schmitz et al. 2021). Active regulatory sequences are typically associated with accessible chromatin, which enables binding of TFs to take place. About 75% of accessible chromatin regions in plants are located outside of transcribed regions but within 2 kb upstream of the transcription start site (TSS) or 1 kb downstream of the transcription termination site (TTS), indicating a much more localized regulation of gene expression compared to animals (Shen et al. 2016; Maher et al. 2017). This aligns with in vivo profiling of sequences bound by TFs in maize (Savadel et al. 2021), which showed that 69% of TFs were bound to sequences in and around genes and 31% were bound to intergenic regions. Likewise, an in vitro profiling method for TF occupancy performed in *Arabidopsis thaliana* (O’Malley et al. 2016) found that CREs were enriched in promoters and 5′ untranslated regions (UTRs) and moderately depleted in coding sequences. Voichek et al. (2024) experimentally confirmed that 5′ UTRs and introns downstream of the TSS play a profound role in TF-mediated transcriptional regulation. In particular, they showed that TFs of the C2C2-GATA family were able to exert a strong positive influence on gene expression, independent of the genomic context, across plant species and most tissues, as long as the respective binding motif was located within 500 bp downstream of the TSS (Voichek et al. 2024). They suggested that the position-dependent activity of plant enhancers is a defining feature that distinguishes them from enhancers in other kingdoms of life. However, there is also increasing evidence for position-dependent regulatory activity of TFs in humans (Barbadilla-Martínez et al. 2026; Duttke et al. 2024) and yeast (Nambiar et al. 2023). Many TF families in plants show characteristic positional binding preferences relative to the TSS (Yu et al. 2016; Chen et al. 2017; Voichek et al. 2024) and, in some cases, such as C2C2-GATA factors, there appears to be a clear functional dependence between their regulatory activity and their binding position relative to the TSS. Nevertheless, it is largely unknown to what extent the function of other TF families depends on their binding position relative to the TSS, since positional preferences can also arise as a byproduct of other dependencies such as the co-occurrence of CREs within CRMs (Acevedo-Luna et al. 2016).

Convolutional neural networks (CNNs), originally developed for image recognition tasks (Lecun et al. 1998; Krizhevsky et al. 2017), have since become well-established as a tool for automatically identifying relevant DNA motifs and learning to interpret their local grammar (Eraslan et al. 2019; Zrimec et al. 2021; Wei et al. 2023). CNNs process input sequences in a layer-wise manner, employing convolutional filters that scan across the input and aggregate local input features into higher-order output features. Various flavors of CNNs have been successfully used to predict gene expression and other molecular phenotypes across a wide range of organisms and were used to gain insight into the regulatory architecture of gene expression at a genome-wide scale (Kelley et al. 2018; Washburn et al. 2019; Agarwal and Shendure 2020; Kelley 2020; Zrimec et al. 2020; Avsec et al. 2021; Wrightsman et al. 2022; Nambiar et al. 2023; Li et al. 2024; Peleke et al. 2024; Wang et al. 2024; Avsec et al. 2026; Linder et al. 2025; Opdebeeck et al. 2025; Qiu et al. 2025). Among them, Agarwal and Shendure (Agarwal and Shendure 2020) created a tissue-agnostic regression model, called Xpresso, for predicting median gene expression in humans and mice from sequence intervals spanning 10.5 kb around the TSS, as well as a set of 8 additional sequence features associated with mRNA decay in mammals. To our knowledge, only 4 models predicting gene expression values (regression models) in plants have been published thus far (Zrimec et al. 2020; Li et al. 2024; Wang et al. 2024; Qiu et al. 2025), and only Li et al. (2024) have performed extensive hyperparameter optimization. They built a CNN-transformer hybrid model called PhytoExpr (subsequently referred to as “PhytoExpr-transformer”) using a concatenated 10 kb input sequence encompassing 5 kb around the TSS and 5 kb around the TTS. Transformers have a highly flexible architecture that can selectively recombine information along the entire input using self-attention (Vaswani et al. 2017). Transformer-like models have been successfully used for gene expression prediction on very large and diverse datasets (Avsec et al. 2021; Li et al. 2024); however, compared to CNNs, they often underperform on small-scale datasets due to having a weaker inductive bias (Akkaya et al. 2024). Li et al. (2024) also built an ensemble model consisting of 27 stacked CNNs that use 6 to 10 kb of proximal regulatory sequences as inputs (subsequently referred to as “PhytoExpr-ensemble”). Ensemble modeling describes a set of techniques for recombining the outputs of an array of smaller submodels into a single output. While this typically leads to an increase in performance and robustness compared to any of the constituent submodels, optimizing an ensemble of models is computationally expensive and doesn’t necessarily provide any benefit over increasing the number of parameters of a single model (Abe et al. 2022). Most recently, Qiu et al. (2025) adapted an existing architecture to identify putative CREs in 4 plant species. Using the previously published Basenji2 architecture (Kelley 2020), a CNN using dilated convolutions and residual connections, with an input sequence of 5 kb around the TSS (subsequently referred to as Basenji-5K), they were able to explain 41% of the variation in maximum gene expression in *A. thaliana*.

Polyploid plants are among some of the world's most economically important crops, such as hexaploid wheat (*Triticum aestivum*), hexaploid sugar cane (*Saccharum officinarum*), tetraploid potato (*Solanum tuberosum*) and tetraploid oilseed rape (*Brassica napus*); however, the complex and redundant structure of their genomes poses many challenges for the analysis of omic data (Cheng et al. 2025). This can also affect the training of sequence-to-expression models due to ambiguous mapping of sequencing reads, making it difficult to gather accurate ground truth data (Page and Udall 2015; Deschamps-Francoeur et al. 2020; Kuo et al. 2020) and arbitrary sequence similarities between related genes, which can lead models to overfit on evolutionary artifacts and overinflate performance estimates (Washburn et al. 2019). Oilseed rape (*B. napus*) is exceptionally well-suited for the study of complex polyploid genomes, due to its close evolutionary relationship with the model plant *A. thaliana* and many other important crops in the mustard family (Brassicaceae), as well as its very recent history of polyploidization (Mason and Snowdon 2016; Friedt et al. 2018). Previously published models for predicting gene expression from genomic sequence have either focused on diploid/paleopolyploid plants or have used a broad range of plants without giving special attention to the difficulties arising from widespread sequence redundancy in polyploids. Here, we devised a strategy for systematically addressing these issues. We achieved state-of-the-art performance in predicting gene expression from proximal noncoding sequences of unseen gene families in allotetraploid *B. napus* using a branched CNN architecture, without the need for ensemble modeling or additional model inputs. We found that the model was able to recapitulate the position-specific upregulation effect of C2C2-GATA TFs recently described by Voichek et al. (2024). Inspection of genes whose expression was poorly predicted by the model revealed an over-representation of genes associated with additional regulatory enhancer elements and Polycomb repressive complexes. Taken together, our results show that sequence-to-expression models are a viable tool for gaining biological insight and generating novel hypotheses in any species of interest, even complex polyploid species, provided that a genome assembly and appropriate gene expression data are available.

## Results

### Deep learning models can be effectively trained on polyploid data

#### *N*emo predicts gene expression from proximal noncoding sequences

**Figure 1:**
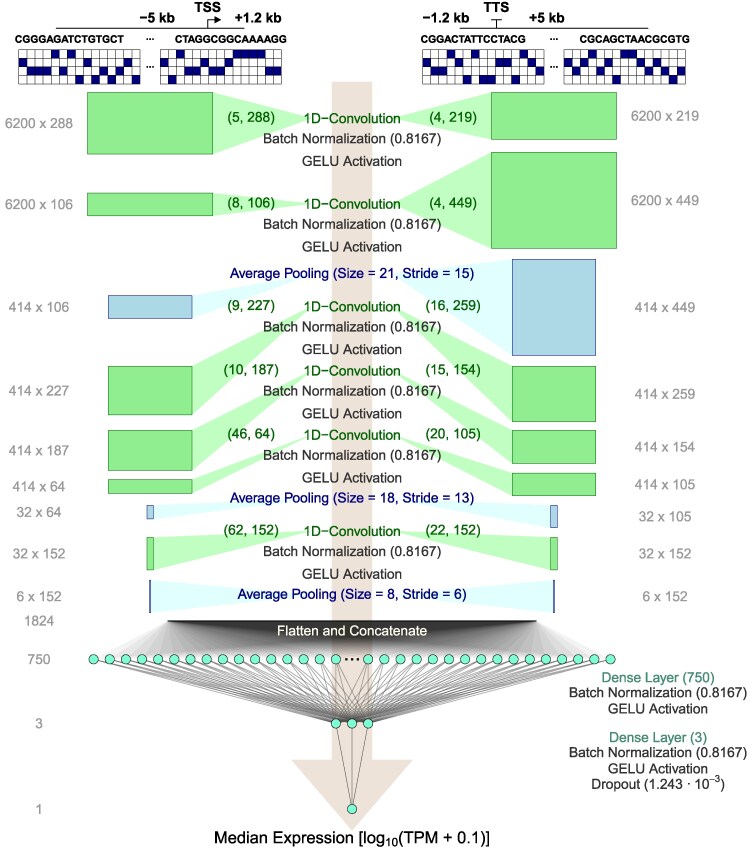
The *n*emo architecture. Optimized hyperparameters of the branched convolutional neural network architecture. Convolutional kernel sizes and the number of filters for each convolutional layer are given in the braces beside the respective layer. The dimensions of boxes are scaled to the output dimensions of the respective layer in the respective branch. Numerical values given for batch normalization layers, dense layers, and the dropout layer represent values for the momentum of the moving average, the number of neurons, and the dropout rate, respectively.

We implemented a branched CNN architecture called *n*emo (***n****apus*  **e**xpression **mo**del), for gene expression prediction from promoter and terminator sequences in allotetraploid *B. napus* (Fig. 1, Figure S1). To train the model, we used RNA-Seq data from a broad range of tissues and developmental stages of *B. napus* cv. Express617, corresponding to the Express617 v1 genome assembly (Lee et al. 2020) from which we gathered the input sequences. The RNA-Seq data included publicly available samples from leaves at 21, 42, and 63 days after planting (DAP) (Calderwood et al. 2021), various seed tissues, as well as silique and gynoecia walls (Woolfenden et al. 2025) and bulk tissue samples from roots, siliques, seedlings, leaves, and flowers (Zanini et al. 2025). Additionally, samples from leaves and apices (7 to 98 DAP) and floral tissues (Sanders stages 6 to 13) were produced for this study (PRJEB107092). All samples were collected from plants grown under standard greenhouse conditions. An overview of all RNA-Seq samples that were used in this study is given in Table S1. During data preprocessing, we attempted to mitigate issues associated with the high degree of sequence redundancy found in polyploid genomes in 4 major ways. (i) We used the EAGLE-RC pipeline (Kuo et al. 2020) to obtain accurate homoeolog-specific expression values. (ii) We discarded homoeologs with identical CDS sequences from the dataset, due to inherent difficulties in obtaining accurate expression values from short RNA-Seq reads. (iii) We used gene family-guided data partitioning based on CDS similarity (Teufel et al. 2023) to partition data into 10 evolutionarily distinct data folds in order to minimize arbitrary sequence relationships between data used for training, validation, and testing, which the model could potentially exploit (Washburn et al. 2019). (iv) Since homology-based methods depend on arbitrary thresholds, we hard-masked coding sequences to further minimize any potentially remaining dependencies that the model could exploit.

**Figure 2:**
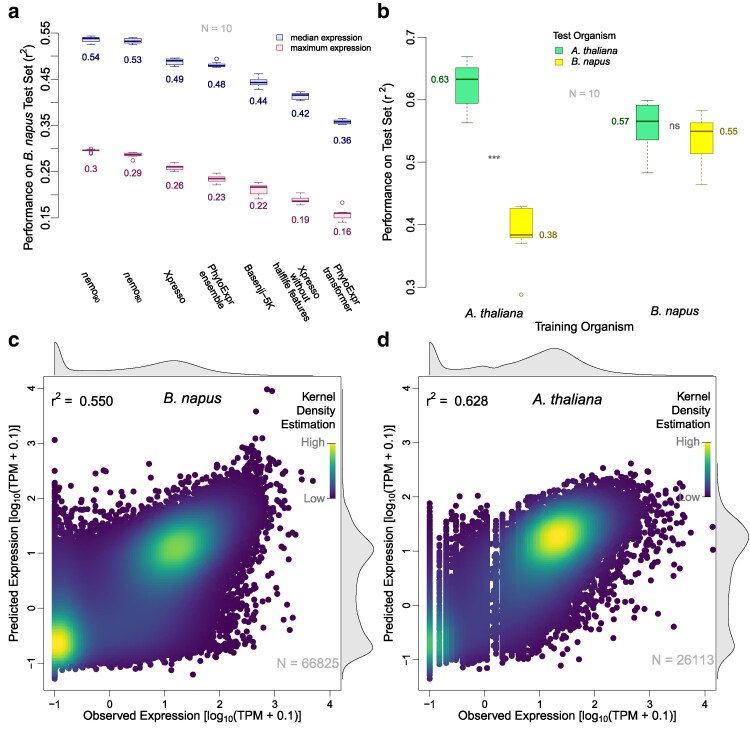
*N*emo robustly predicts median gene expression. a) Performance comparisons on unseen *B. napus* test data. Models were trained to predict median and maximum expression, respectively. Numbers below boxes indicate median performance across 10 replications. From left to right: *n*emo_90_, trained on the same training set, consisting of all the data used during hyperparameter optimization, including the validation set; *n*emo_80_ instances, trained on the 80% of data used as a training set during hyperparameter optimization; Xpresso; PhytoExpr-ensemble; Basenji-5K; Xpresso without halflife features and PhytoExpr-transformer, all trained on the same 80% of data and tested on the same unseen test data. b) Cross-species comparison between *n*emo_90_ trained on *B. napus* data and *n*emo_90_ trained on *A. thaliana* data. The box plots represent test performance across all 10-fold permutations. Median performance across data folds is indicated beside each box. *B. napus* test sets were randomly down-sampled to match the smaller *A. thaliana* test set size. c, d) Minimally biased predictions on jointly partitioned data. Ten instances of *n*emo_90_ were trained on different permutations of data folds from *B. napus* (c) and *A. thaliana* (d), respectively. Each instance was tested on the respective held-out test set. All 10 sets of test predictions for each species were then concatenated and plotted against the observed median expression to arrive at a minimally biased set of predictions across the entire dataset. Colors represent Gaussian kernel density estimations (KDE).

We focused on the prediction of median gene expression across tissues, which was supported by a high overall correlation between tissues (Figures S2 and S3) and has previously been successfully performed on human and mouse data (Agarwal and Shendure 2020) and data from various plant species (Li et al. 2024). The *n*emo architecture contains separate convolutional branches to independently extract features from the 2 sequence inputs. The model inputs span 5 kb upstream of the TSS to 1.2 kb downstream of the TSS and 1.2 kb upstream of the TTS to 5 kb downstream of the TTS, respectively, which were determined to be the optimal input sequence lengths during hyperparameter optimization. Hyperparameters were optimized extensively over a large search space in 6 stages, covering general architectural parameters in the first 3 stages (Tables S2 to S4) and focusing mainly on parameters related to the training process in the last 3 stages (Tables S5 to S7). The main architectural difference of the *n*emo architecture compared to previously published sequence-to-expression models is the use of average pooling with overlapping pooling windows, which was found to increase performance during hyperparameter optimization. This has the effect of decreasing translational invariance while increasing spatial resolution, compared to the common approach of using nonoverlapping max-pooling windows, suggesting that the exact relative positions of sequence features play an important role in making accurate predictions. The main difference in the approach we took to training the model is the use of a fixed training regimen of 6 epochs, using a one-cycle learning rate policy (Smith 2018), where we slowly and gradually increase the learning rate up to a fairly large maximum value of 0.0663, after which it is rapidly reduced below the initial learning rate (Figure S4a). Empirically, we found that this resulted in consistent and reproducible model convergence between training runs, illustrated by low variability in validation loss at the end of training runs (Figure S4b), as well as good generalization on unseen data (Fig. 2), suggesting that this approach helped the training algorithm converge to flat local minima in the loss landscape (Smith and Topin 2019). The performance of the *n*emo architecture, trained on a fixed 80% split of the *B. napus* data (*n*emo_80_) and tested on a held-out test set (10% of the data) containing unseen gene families, performed better (*r*^2^ = 0.53) than 4 previously published architectures (Xpresso: *r*^2^ = 0.49, PhytoExpr-ensemble: *r*^2^ = 0.48, Basenji-5K: *r*^2^ = 0.44, PhytoExpr-transformer: *r*^2^ = 0.36), which we re-implemented, trained and tested on the same datasets (Fig. 2a). Except for minor modifications needed to ensure model convergence (see materials), we stuck to the original implementations in terms of the architecture, the input sequence intervals and the training regimens. When incorporating the validation set into the training set and therefore training the *n*emo architecture on 90% of the data (*n*emo_90_), the performance on the held-out test set increased slightly (*r*^2^ = 0.54). This was possible due to the fixed training regimen that did not depend on monitoring validation loss to stop the training process. The decision to mask coding sequences was supported by the observation that, while it marginally reduced model performance, it also reduced the difference in performance between gene family-guided partitioning and random partitioning (Figure S5).

When training the architectures to instead predict maximum expression across all samples, the order of best to worst performing architectures remained the same; however, overall performance was much lower than for the prediction of median expression (Fig. 2a). Interestingly, even Basenji-5K, which was reported to be able to explain 41% of the variation in maximum expression in *A. thaliana* (Qiu et al. 2025), was only able to explain 22% of maximum expression on the *B. napus* data used in this study. This drop in performance may be explainable in part by the switch from a simple diploid model organism to a more complex polyploid organism, which may be more difficult to predict; however, it may also be partially based on the masking of coding sequences in our dataset, suggesting that memorization of gene families could have been a contributing factor.

Unlike the Xpresso architecture, the *n*emo architecture does not rely on additional hand-crafted sequence features. However, the *n*emo architecture is also much larger, with a little less than 7 · 10^6^ trainable parameters compared to approximately 10^5^ trainable parameters for the Xpresso architecture. When excluding the halflife features from the input, the performance of the Xpresso architecture was second to last among all tested architectures (*r*^2^ = 0.42), showing that its additional input features partially explain its high performance despite the comparatively low number of model parameters (Figure S6). In its original form, the Xpresso model receives, among other features, the GC contents of coding sequences, whereas the other models have no access to the coding sequence at all, perhaps giving the Xpresso architecture an unfair advantage. Some of the performance of the *n*emo architecture may be attributable to the large sequence context to which it has access (12.4 kb). It is followed by Xpresso (10.5 kb), Basenji-5K and PhytoExpr-transformer (10 kb), and PhytoExpr-ensemble, where each individual submodel has its own input that is 6 to 10 kb in length. The masking of coding sequences may have affected some architectures disproportionately, in particular Xpresso and Basenji-5K, for which the gene body makes up a large portion of the input sequence. The performance of the *n*emo architecture on *B. napus* data was similar to the reported performance of the Xpresso architecture on human data, which it had been optimized for (*r*^2^ = 0.59) (Agarwal and Shendure 2020). Although these performance estimates cannot be directly compared between studies due to differences in data preprocessing, this result gives a rough indication that proximal genomic sequences may have a similar potential for explaining gene expression in plants and animals, respectively. This is consistent with Li et al. (2024), who reported similar performance estimates in plants, and Zrimec et al. (2020), who found that a gene expression model trained on data from *A. thaliana* only performed marginally better than the same model trained on human data, despite large differences in gene density between the 2 species. To our knowledge, there is no published sequence-to-expression model trained only on proximal regulatory sequences in plants that has a substantially higher performance than the performance reported here. Taken together, based on the performance of various proximal sequence-to-expression models, including our own, the proportion of proximal gene regulation does not appear to be substantially higher in plants compared to mammals, despite large differences in chromatin organization that would suggest otherwise (Liu et al. 2016; Maher et al. 2017). However, this is contingent on taking the various performance estimates at face value and would need further investigation to confirm. An alternative interpretation could be that proximal regulatory patterns in plants are harder to learn for contemporary sequence-to-expression models, suggesting perhaps a more complex grammar of regulatory elements or a higher proportion of proximal regulatory information that is modulated epigenetically.

To test the contributions of the 2 input sequences to the predictive performance of the *nemo* architecture, we derived variants of all data folds, where we randomized the upstream and downstream region of promoter or terminator sequences, while maintaining the pattern of masked segments, and performed test predictions on these modified inputs using the *nemo*_90_ instance that had not been trained on the respective data fold (Figure S7). When randomizing the terminator sequence upstream of the TTS (*r*^2^ = 0.48) or downstream of the TTS (*r*^2^ = 0.45) while maintaining all other sequence regions, *n*emo was able to explain 7% to 10% less of the variation in gene expression compared to the unmodified inputs, indicating that the contribution of the terminator sequence to the model's predictions is nonzero, but fairly marginal. However, when randomizing the promoter sequence upstream of the TSS, the correlation between predicted and observed gene expression disappeared almost entirely (*r*^2^ = 0.11). Randomizing the downstream promoter affected model performance to a lesser extent, but still substantially (*r*^2^ = 0.36). However, it is also important to note that the regions downstream of the TSS and upstream of the TTS are shorter (1.2 kb) than the respective regions outside of the gene body (5 kb). Overall, this analysis suggests a crucial role of the promoter region, which includes the 5′ UTR, in setting a coarse baseline of expression, which then may be up- or down-regulated by CREs in the terminator and distal enhancers.

#### Polyploid training data increases model robustness

For cross-species performance comparisons, we jointly partitioned data from *B. napus* and *A. thaliana* so that evolutionary dependencies would only occur between the corresponding data folds of the 2 species. We then performed 10-fold cross-testing where, for each species, we trained 10 *n*emo_90_ model instances on 90% of the data. Each model instance was trained on a different permutation of data folds and tested on the remaining data fold of the same species and the corresponding data fold from the other species (Fig. 2b). The predictions made by the 10 model instances of each species were aggregated to obtain minimally biased predictions across the complete datasets of both species (Fig. 2c and d). Since predictions made on the jointly partitioned data were also made on gene families that were used during hyperparameter optimization, the cross-species performances should only be evaluated in relation to each other and not in absolute terms. The *n*emo_90_ instances trained on *A. thaliana* data were able to explain about 8% more of the variation in observed median expression in *A. thaliana* (*r*^2^ = 0.63) than the model instances trained on *B. napus* data were able to explain in *B. napus* (*r*^2^ = 0.55). However, while the model instances trained on *B. napus* data showed no significant difference in performance when tested on data from *A. thaliana* (*r*^2^ = 0.57; Wilcoxon rank sum test: *N* = 10, *P* = 0.2475), the model instances trained on data from *A. thaliana* performed significantly worse when tested on data from *B. napus* (*r*^2^ = 0.38; Wilcoxon rank sum test: *N* = 10, *P* = 1.083 · 10^−5^) (Fig. 2b). This indicates that models trained on data that is based on a manually curated high quality genome annotation, such as TAIR10 (*A. thaliana*) (Lamesch et al. 2012), may be able to achieve a higher peak performance when tested on data from the same source, since TSSs, TTSs, and intron–exon boundaries are well defined, but do not perform robustly on data that is based on minimally or noncurated annotations, which may contain less well-defined structural features and a higher rate of pseudogenes. On the other hand, previous studies have also reported immense difficulty in transferring models from simple diploid genomes to complex polyploid genomes (Qiu et al. 2025). Models trained on polyploid genomes are exposed to a larger quantity of data and, in particular, a larger quantity of redundant sequences and, therefore, may be able to implicitly learn how variations between similar sequences affect gene expression. In situations where the number of training examples is limited, data augmentation can help to improve models (Opdebeeck et al. 2025); however, we argue that training models on polyploid organisms provides an inherent form of data augmentation. Gene family-guided partitioning of data not only prevents data leakage between sets but also maximizes the sequence redundancy that the model is exposed to during training, since each gene family is concentrated in one of the sets, instead of being scattered between sets. The fact that model instances trained and tested on data from *A. thaliana* achieved a higher peak performance than model instances trained and tested on data from *B. napus* served as an encouraging sign that the high degree of sequence redundancy found in the *B. napus* genome had been accounted for and therefore did not lead to overinflated performance estimates.

#### *N*emo predictions are consistent with real-world gene expression variation

**Figure 3:**
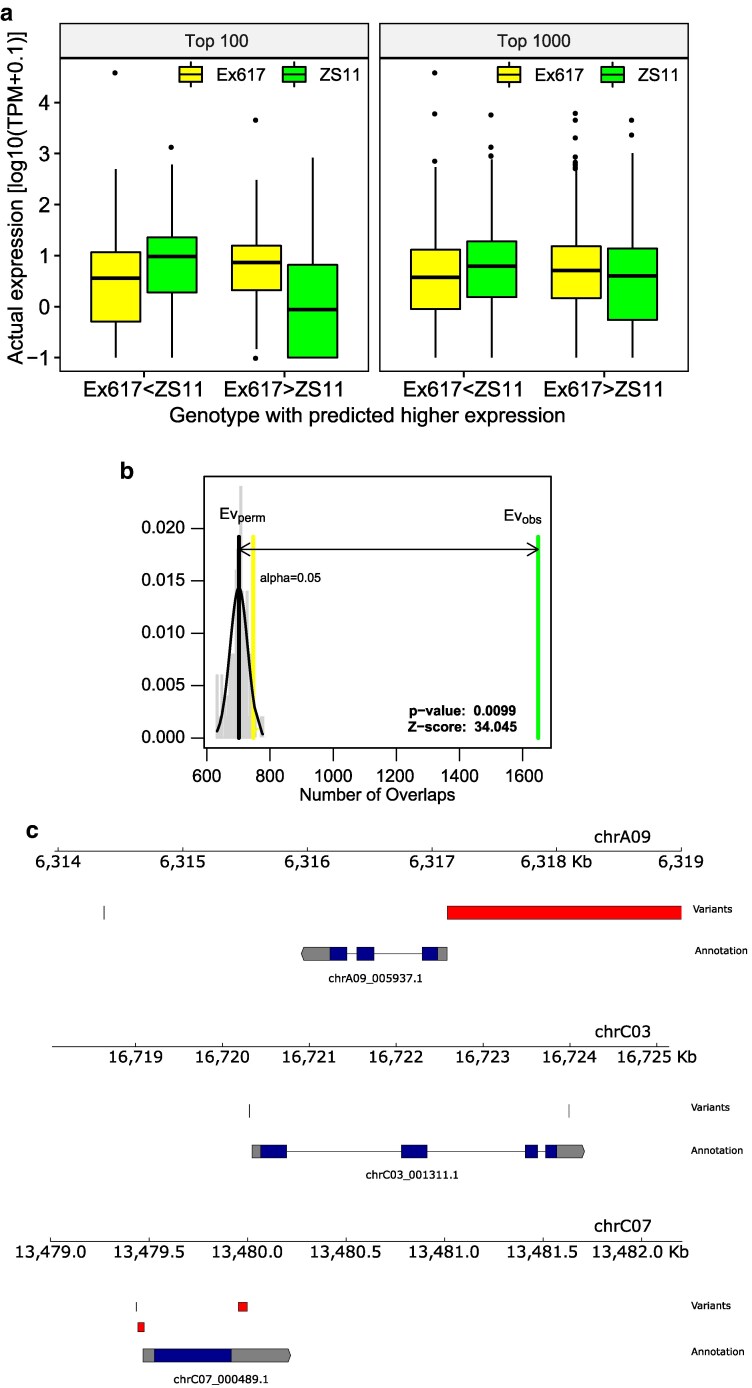
Validation of *n*emo_100_ gene expression prediction across *B. napus* ecotypes. a) Comparison of gene expression between ecotypes ZS11 and Express617 for the top 100 (left) and top 1,000 (right) genes with the highest predicted difference in expression in either direction. b) Permutation test checking for enrichment or depletion of SVs intersecting with extended gene bodies (±1 kb) predicted to have a difference in expression between ecotypes. EV_perm_ is the expected number of overlaps from random permutations; EV_obs_ is the observed number of overlaps. The significance threshold is denoted as alpha. The *P*-value denotes the probability by which the observation would be expected by random chance, and the *Z*-score denotes the number of standard deviations that the observed value differs from the mean of permutations. c) Example of genes which are predicted to have higher expression in Express617 (reference) compared to ZS11. Variants in the ZS11 genome are shown as thin black lines (insertions) and red boxes (deletions), relative to the reference. The ZS11 genome was aligned to Express617 genome using minimap2. SVs were called using SVIM-asm. In total, 43,657 variants were discovered.

To further validate *n*emo predictions, we tested whether the predictions made by *n*emo_100_, trained on the full dataset, agreed with differences in gene expression observed across 2 oilseed rape ecotypes: Express617 (winter type) and ZS11 (semi-winter type). The structural gene annotation was transferred from Express617 to ZS11, and gene expression prediction was made for all genes across the 2 ecotypes (Figure S8). We then selected the top 100 and 1,000 genes with the most extreme differences in predicted expression levels between the 2 ecotypes, meaning where the predicted Express617 expression was either higher (Express617 *>* ZS11) or lower (Express617 *<* ZS11) than ZS11. Actual (observed) gene expression levels were then compared between ecotypes. The model was able to correctly identify the allele with higher expression in 77.5% of cases for the top 100 genes and 60.4% for the top 1,000 (Fig. 3a). Recently, it was postulated that structural variants (SVs) play an important role in rewiring gene expression in *B. napus* (Zhang et al. 2024b; Yildiz et al. 2025). Presence of SVs, which either delete or insert regulatory motifs, could be one of the causes of differences in expression between Express617 and ZS11. We therefore tested if the genes predicted to have different expression across the 2 ecotypes are over-represented in SVs, and indeed observed significant over-representation of this subset (Fig. 3b and 3c). However, more detailed, higher-resolution, genome-wide mapping of SVs to regulatory motif insertion/deletion would be needed to investigate whether these variants affect gene regulation.

### CNNs can learn positional binding preferences of plant TFs

#### Importance scores reflect experimentally derived binding preferences of TFs

**Figure 4:**
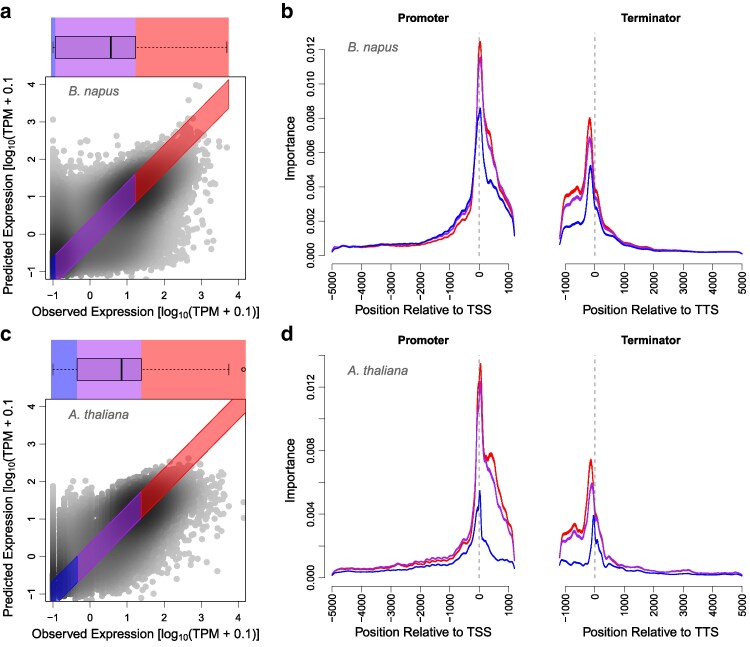
Expression-specific importance. a, c) Segmentation of well-predicted genes (colored areas) into highly (red, upper quatile), moderately (purple, interquartile range), and lowly (blue, lower quartile) expressed groups, based on the upper and lower quartiles of observed expression values for *B. napus* and *A. thaliana*, respectively. b, d) Metagene plots of group-specific importance scores (sum of absolute Shapley values, averaged across genes) along the 2 input sequences for *B. napus* and *A. thaliana*, respectively.

In order to assess which sequence features the model predictions were based on, we attributed prediction outcomes to features in the input sequences of specific genes using Shapley values (Lundberg and Lee 2017), which were then used to calculate importance scores across genes. To gain an overall picture, we first classified well-predicted genes into 3 expression bins using the upper and lower quartiles of observed expression values as thresholds (Fig. 4a and c), and calculated importance scores across all genes in the 3 respective groups (Fig. 4b and d). For all 3 expression classes, the 5′ and 3′ UTRs were, on average, more important for making predictions than the regions upstream of the TSS and downstream of the TTS. While this seemingly contradicted our bulk randomization experiments (Figure S7), this result is in agreement with similar findings from Washburn et al. (2019), as well as Peleke et al. (2024). Due to the additive property of Shapley values (Lundberg and Lee 2017), meaning that attribution values add up to the difference between the predicted value and a reference value (which is based on random sequences and therefore expected to be close to the minimum value), highly expressed genes are expected to be associated with high importance scores. The differences between the 3 expression classes tended to be most pronounced in the UTR regions, with importance scores for highly expressed genes being higher than for lowly and moderately expressed genes. On the other hand, in the promoter regions upstream of the TTS in *B. napus*, lowly and moderately expressed genes tended to have slightly higher importance scores than highly expressed genes (Fig. 4b). We hypothesized that some of the observed patterns could be due to differential presence and effect of TF binding sites.

**Figure 5:**
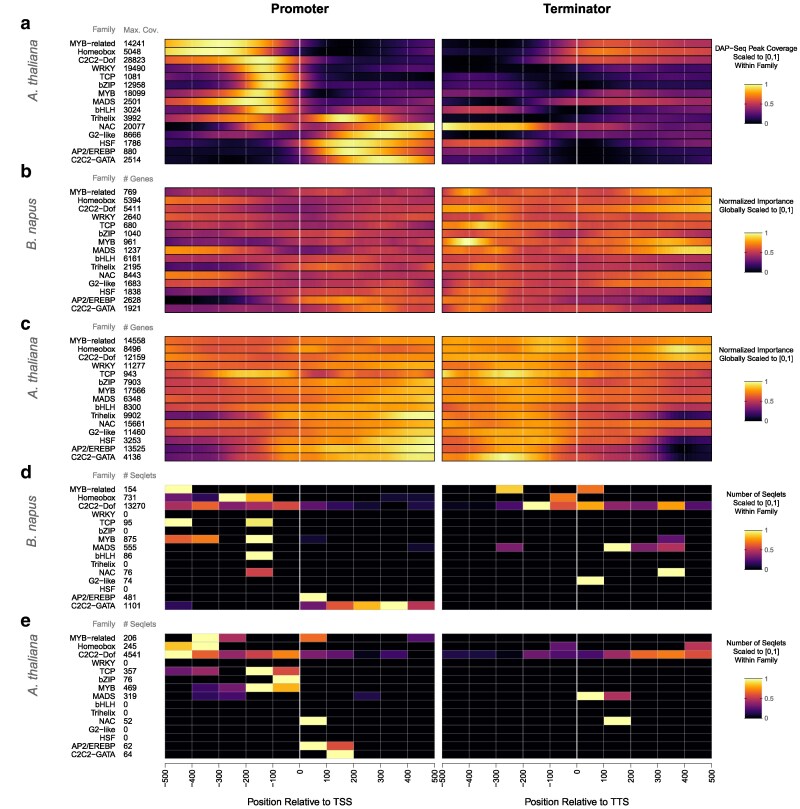
Group-specific positional preferences learned by *n*emo. a) DAP-Seq signals showing TF-binding positions for 15 TF families averaged across genes and scaled to the [0, 1] range across the promoter and terminator within each TF family. b, c) Normalized importance profiles of *n*emo_90_ for well-predicted genes in which a DAP-Seq peak of the respective family intersected within ± 500 bp up- or downstream of the TSS, for *B. napus* and *A. thaliana*, respectively. The normalized group-specific importance scores were scaled to the [0, 1] range globally across all TF families and both input sequences. d, e) Relative number of TF-MoDISco seqlets matching a TF of the respective family, within 100 bp bins. Scaled to [0, 1] within each family. a to e) Legends on the left show maximum DAP-Seq peak coverage, numbers of genes associated with each TF family, and the numbers of seqlets that were found for each family, respectively. a to c) Values were smoothed using a sliding window of 101 bp.

To investigate the possibility of importance scores reflecting distinct effects of different TF families, *A. thaliana* genes were classified based on experimentally derived information (DAP-Seq; O’Malley et al. 2016) on TFs binding to their promoters (TSS ± 500 bp). Genes of *B. napus* were then classified based on homology to the respective *A. thaliana* genes. We chose a selection of 15 of the largest TF families (at least 10 members), that had been shown to have strong positional binding preferences. Importance scores were calculated for each group and normalized (Figures S9 and S10) to highlight position-specific differences between the groups (Fig. 5b and c). When interpreting these results, it is important to note that while DAP-Seq results highlight binding preferences of specific TF families (Fig. 5a), the importance scores for the corresponding genes (Fig. 5b and c) will highlight all the sites that are important for predictions, for example, reflecting the presence of motifs for multiple families. However, even accounting for the resulting signal diffusion, for promoters and terminators of both species, the normalized importance scores (Fig. 5b and c, Figure S11 to S16) of some of the groups showed a clear correspondence with the respective DAP-Seq profile (Fig. 5a). TFs of the Homeobox and C2C2-Dof families have a preference for binding up to several hundred base pairs upstream of the TSS and downstream of the TTS. Normalized importance scores for these families highlighted this region to a varying but distinguishable degree in both species, especially when compared to genes associated with TF families that have a preference for binding closer to the gene body. Within the groups themselves, for *A. thaliana* in particular, normalized importance scores appeared more or less constant along the displayed regions of the input sequence, more so than in many of the other groups, making it impossible to decipher a family-specific signal that is independent of the signal produced by other families. In *B. napus*, the region upstream of the TSS in genes associated with MYB-related factors appeared to be less important than the region downstream of the TSS, despite DAP-Seq data indicating a strong preference for these factors to bind upstream. While a pattern shift like this might indicate some regulatory divergence between the 2 species, it appears more likely that this is a data artifact that was either caused by model bias or introduced during downstream data processing, such as the homology-based classification of *B. napus* genes. For TFs of the MADS family, which also have a preference for binding outside the gene body, the normalized importance scores in *B. napus* clearly highlighted these regions and mirrored the DAP-Seq signal (Fig. 5b, Figure S13). For *A. thaliana*, normalized importance scores at least highlighted the regions outside the gene body in comparison to genes associated with TF families that tend to bind inside the gene body. For TFs of the TCP family, which preferentially bind immediately upstream of the TSS, the corresponding normalized importance scores distinctly highlighted this area in the normalized importance scores of *A. thaliana* (Fig. 1c, Figure S12). For both species, the same was also true for TF families that preferentially bind downstream of the TSS and upstream of the TTS, such as HSF, AP2/EREBP, and C2C2-GATA (Figures S15 and S16). When taking into account the entire interval ± 500 bp around the TSS, we observed particularly strong positive Spearman correlations between the DAP-Seq signal of the 3 TF families that have the strongest tendency to bind downstream of the TSS (HSF, AP2/EREBP and C2C2-GATA) and the respective normalized importance scores in both species (Figure S17a and b). Some of the discrepancies between DAP-Seq signals and normalized importance scores may be explained by the presence of other important features, such as co-occurring CREs that bind TFs of other families (Figure S17c); however, these may also reflect model-specific artifacts. A sharper boundary between importance scores of upstream and downstream positions was observed in *A. thaliana* (Figure S10) compared to *B. napus* (Figure S9), indicating a lower precision of annotated TSSs in *B. napus*.

We validated the general patterns found in the analysis of normalized importance scores by analyzing the enrichment of seqlets (small sequence fragments found in regions with high positive or low negative Shapley values) that were identified by TF-MoDISco (Shrikumar et al. 2018), within 100 bp bins in the ± 500 bp region around the TSS and TTS. TF-MoDISco aligns seqlets into consensus patterns and matches these patterns with known TF-binding motifs. We attributed each seqlet to the closest matching TF binding motif that was found for the respective pattern and, by extension, associated them with the corresponding TF families (Fig. 5d and e). This analysis mostly confirmed the patterns described above but also provided complementary information in some instances. For example, while the seqlet enrichment did not show MADS TFs binding upstream of the TSS in *B. napus*, it highlighted the binding of MYB-related factors upstream of the TSS, which the normalized importance scores did not show. The analysis of seqlets also confirmed that the model had learned the binding patterns of C2C2-GATA factors downstream of the TSS. However, for several TF families, TF-MoDICco was only able to find a few or zero matching seqlets.

#### In silico mutagenesis suggests position-specific effects of some TF families

**Figure 6:**
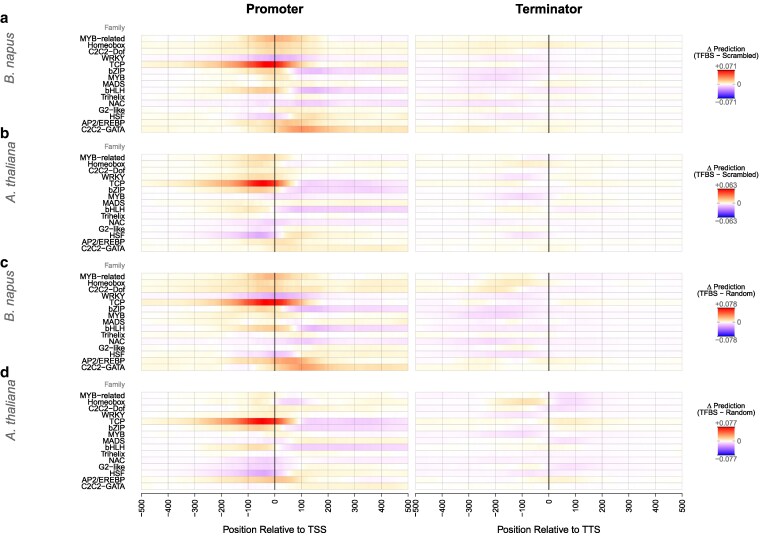
Position-specific effect of motif insertions on predicted expression. a to d) Median predicted difference in expression [log_10_(TPM + 0.1)] between test and control sequences. For each TF family and position, 10 moderately expressed genes were randomly selected to create test sequences by inserting 10 randomly selected TF-binding motifs of the respective family at the respective positions (denoted as TFBS). For each test sequence, control sequences were created by (a, b) scrambling the respective TF-binding motif or (c,d) replacing it with a random motif of equal length. Color values were scaled with respect to the maximum absolute predicted expression difference across all families and both input sequences. Red color indicates increased expression of the test sequence (TF-binding motif inserted) with respect to the control sequence; blue indicates decreased expression. a to d) Values were smoothed using a sliding window of 101 bp.

Recent experimental evidence suggests that plant and animal TFs can exert position-specific effects on gene expression (Duttke et al. 2024; Voichek et al. 2024). We performed in silico mutations to confirm that the model had not only learned to recognize CREs, which happened to be preferentially located in specific regions in the input, but also learned to associate specific motifs and their effect with specific regions. We inserted 10 randomly selected motifs of each family at each position ± 500 bp around the TSS and TTS of moderately expressed genes whose promoters did not intersect with a DAP-Seq peak of the respective TF family, and measured how the predicted expression changed with respect to the location of the insertion (Fig. 6, Figures S18 to S26). By measuring the median predicted change in expression across different sequence contexts, we focused on the additive effects of individual motifs of a given family, disregarding interactions with other motifs. Because it is possible for the model to learn positional sensitivity to sequence composition or GC content rather than biologically meaningful motif-position interactions, we included three types of negative controls (random—no preservation of sequence composition, scrambled—sequence composition preserved, reversed—sequence composition preserved). In the promoter region, some position-specific signal was observed for scrambled and reversed motif controls, especially for the TCP, bZIP, MYB, and AP2/EREBP families (Figures S24 to S26). In the terminator region, all controls displayed a position-specific signal just upstream of the TTS (Figure S24). To quantitatively assess the effects of motif-position interactions above the background level, the change in expression was measured relative to changes caused by insertion of control sequences, which were constructed from the same underlying wild-type sequences by inserting either a scrambled version of the respective motif to control for GC-content (Fig. 6a and b), or a random motif of equal length to control for purely positional effects that are independent of the motif (Fig. 6c and d). We observed distinct patterns for some TF families that were mostly consistent between the 2 species.

To our knowledge, C2C2-GATA TFs are the only family with extensive experimental validation demonstrating a position-specific effect (expression upregulation only if found downstream of TSS) across a number of species (Voichek et al. 2024). Concordantly, our in silico motif insertion analysis predicted increased expression following C2C2-GATA motif insertion, but only within a few hundred bp downstream of the TSS (Fig. 6). This effect was particularly evident in *B. napus* (Fig. 6a and c, Figure S22). A positive change in expression was also predicted for AP2/EREBP motifs inserted immediately downstream of the TSS (Fig. 6, Figures S22 and S23), with some spillover to upstream positions. This result is roughly consistent with the observed positional binding preference of AP2/EREBP factors (Fig. 5a); however, a similar pattern also emerged when inserting reversed or scrambled versions of AP2/EREBP motifs (Figure S24). The effect sizes were also in a similar range (42% to 119%) compared to insertions of intact motifs and reflected in Fig. 6, where subtracting the effects of controls weakened the position-specific signal. When controlling for GC-content (Fig. 6a and b), the pattern was much weaker than when just controlling for positional bias (Fig. 6c and d), indicating that, in this case, the position-dependent effect learned by the model may be largely based on sequence composition (AP2/EREBP motifs are GC-rich) rather than exact motifs. A strong positive change in predicted expression was observed in both species when TCP motifs were inserted around the TSS (Figures S18 and S19), which is consistent with experimental data pointing to TCP factors binding in the immediate proximity of TSSs (Fig. 5a). TCP motifs also produced similar patterns, when reversed or scrambled, especially in *A. thaliana*, however, in this case the effect sizes were much smaller relative to insertions of intact motifs (8% to 23%), consistently the position-specific signal remained after subtracting the effects of controls (Fig. 6, Figure S24). The predicted effect size for intact TCP factors was also much larger than for intact C2C2-GATA and AP2/EREBP factors. Interestingly, for bHLH factors, which have a bimodal binding pattern, frequently binding directly upstream of the TSS and less frequently 150+ bp downstream of the TSS (Fig. 5a), the model associated a positive change in expression with upstream motifs and a negative change in expression with downstream motifs (Fig. 6, Figures S20 and S21). A similar pattern was predicted for bZIP factors, whereas the opposite pattern was predicted for HSF factors. NAC factors were predicted to have a predominantly negative influence on gene expression when inserted in the promoter sequence. Especially in *B. napus*, the highest effect sizes for MYB-related factors were predicted in the immediate vicinity of the TSS, which was unexpected considering the DAP-Seq signal, suggesting a strong preference to bind further upstream. The predicted effect of the remaining TF families was either weak or inconsistent between species. Insertions of random motifs with the same lengths as the corresponding TF binding motifs in the 3′ UTR region upstream of the TTS consistently resulted in an increase in predicted expression (Figure S24g and h), indicating that insertions in this region may disrupt other predictive features, perhaps linked to mRNA turnover (Cui et al. 2020; Zhang et al. 2024a). A similar but opposite pattern (predicted downregulation in response to almost any kind of insertion) was observable downstream of the TSS (Figure S24g and h), suggesting this region may predominantly harbor features that have an upregulating effect, which then get disrupted by the inserted sequences.

### Epigenetics and distal cis-regulation influence prediction outcomes

**Figure 7:**
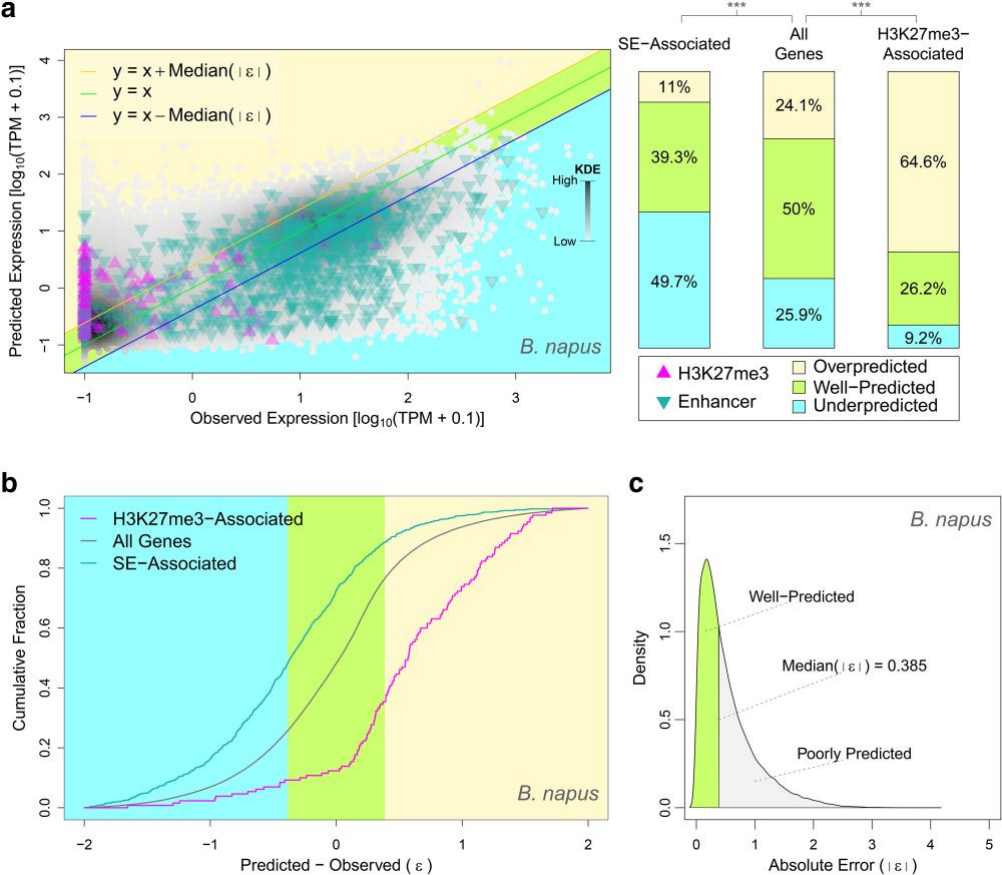
Regulatory features not captured by the model. a) Relative proportions of prediction error-based gene classifications for genes associated with H3K27me3 histone marks (identified by homology to published *A. thaliana* genes) or nearby SEs (based on a published set of SE-associated genes in Express617), compared to the overall distribution. b) Empirical cumulative distribution function of prediction errors for H3K27me3-associated genes, SE-associated genes, and all genes, respectively. c) Distribution of absolute prediction errors.

While inspecting prediction results, we observed distinct groups of genes whose expression was either over- or underpredicted by our model. We hypothesized that those genes could be under additional control either by additional CREs or epigenetic factors. We therefore compared them with identified *B. napus* genes that are associated with enhancer elements and H3K27-trimethylation, respectively. H3K27me3 histone marks are deposited by Polycomb proteins and are typically found in stably repressed genes (Xiao and Wagner 2015). Super-enhancers (SEs) are clusters of enhancers that can increase the expression of cognate genes over long distances and typically have a strong effect on surrounding genes (Zhao et al. 2022). We identified genes associated with H3K27-trimethylation via homology to Polycomb-repressed *A. thaliana* genes (Borg et al. 2020) and used a published set of SE-associated genes in *B. napus* cv. Express617 (Zanini et al. 2025). To assess the effect of these factors on prediction errors, *B. napus* genes were categorized based on the magnitude and directionality of their prediction error (predicted expression − observed expression). Up to an error of ± the median absolute error across all genes, predictions were considered good (Fig. 7c). If the error was above this range, the respective gene was considered overpredicted. If it was below this range, it was considered underpredicted (Fig. 7a). By definition, well-predicted genes make up 50% of the entire dataset. The other 50% are distributed among the other 2 error-based classes.

When comparing the overall distribution of the error-based classes with the distributions of genes associated with SEs and H3K27-trimethylation, there was a clear enrichment of underpredicted genes among the subset of genes that were associated with SEs (*χ*^2^-test: *N*_SE,over_ = 105, *N*_SE,well_ = 376, *N*_SE,under_ = 475, *N*_all,over_ = 16,116, *N*_all,well_ = 33,413, *N*_all,under_ = 17,296; *P* < 2.2 · 10^−16^) and an enrichment of overpredicted genes among those associated with H3K27me3 histone marks (*χ*^2^-test: *N*_H3K27,over_ = 84, *N*_H3K27,well_ = 34, *N*_H3K27,under_ = 12, *N*_all,over_ = 16,116, *N*_all,well_ = 33,413, *N*_all,under_ = 17,296; *P* < 2.2 · 10^−16^). Importantly, SE-associated genes (Zanini et al. 2025) and underpredicted genes (Figure S27) were not more tissue-specific than well-predicted genes, suggesting that the prediction error for underpredicted genes was not a result of more tissue-specific expression patterns. This suggests that the model was not able to properly predict the influence of epigenetic and potentially more distal and complex cis-regulatory features. This could be because the enhancer elements were found too far away or because the model clearly focused on features proximal to the TSS and TTS, respectively (Fig. 4b). The outside influence of enhancers may lead to expression levels that are higher than local regulatory features would suggest, leading the model to predict lower expression levels than what is observed. The opposite of this applies to H3K27me3 histone marks, where, based on features in the local DNA sequence, the model may overestimate the expression of genes that are epigenetically silenced. This suggests that there is a subset of genes for which the proximal cis-regulatory logic encoded in the DNA comes into effect only after epigenetic constraints have been lifted.

## Discussion

Deciphering the gene regulatory architecture of plants remains a challenging task, yet deep learning models are remarkably adept at identifying relevant patterns and predicting gene expression from genomic sequences. We were able to show that a well-optimized CNN model is able to explain more than 50% of the variation in median gene expression, even in complex polyploid plants such as *B. napus*, using only proximal noncoding sequence information. We were able to outperform 4 other competing state-of-the-art architectures when trained and tested on the same data. By systematically mitigating issues associated with sequence redundancy in polyploids and demonstrating the feasibility of training useful and generalizable models from polyploid data, we hope to establish best practices and inspire similar work in the future.

By randomizing regions in the promoter and terminator inputs, respectively (Figure S7), we found that despite using a highly flexible architecture that can learn separate motifs and patterns for the promoter and terminator, most of the model's performance depended on the promoter input alone. However, since the 3′ UTR has been hypothesized to be important in fine-tuning gene expression levels (Washburn et al. 2019), this finding may not hold for predictions of tissue- or condition-specific gene expression. Compared to importance scores derived from Shapley values, the almost complete loss of predictability when randomizing the upstream promoter suggests that global importance scores may not show the full picture and may have a bias for regions harboring features that have a strong and consistent effect. Although Shapley values can be negative and importance scores are based on absolute values, expression values have a lower bound, which is close to the reference value that is used to calculate attributions. This may make it difficult to detect features that have a negative impact on prediction in certain circumstances. For example, if the predicted value is low but higher than the reference value, the average attribution value is expected to be low but positive, which may suggest a low importance. However, regions that harbor features that are perhaps more variable and context-dependent may still be crucially important in their entirety and on the level of individual genes. Although it is possible to train performant regression models for tissue-agnostic gene expression across species (Li et al. 2024), they are ultimately limited to general patterns that are transferable between species. Species-specific models have the potential to yield insights that are more relevant to the species of interest. Furthermore, even though general regulatory patterns may be similar, the structure and function of tissues and the adaptive strategies to environmental cues may differ significantly between species, making it difficult to train condition- or tissue-specific models across species.

Nevertheless, by calculating importance scores and devising a normalization strategy to highlight group-specific regional differences in importance, we were able to partially recapitulate the proximal positional binding preferences of TF families. Genes regulated by specific TF families showed specific regional importance patterns that were largely reproducible between models trained on data from *B. napus* and *A. thaliana*, respectively, and roughly coincided with TF binding profiles gathered from DAP-Seq data in *A. thaliana*. Interestingly, we observed that TFs, whose effect appeared to be strongly upregulating, tend to bind downstream of TSSs (Figure S28), which may contribute to the high importance of UTR regions in plants implied by deep learning models. Since CRMs typically contain multiple CREs that bind specific combinations of TFs, single motifs may not be predictive of gene expression outcomes (Voichek et al. 2024). Therefore, it is not surprising that other regions, in addition to the observed TF-binding regions of the respective TF family, were often also important for predicting the expression of the respective genes. In addition, other regulatory patterns not related to TF-binding may have also been learned by the model.

By inserting individual TF binding motifs in various locations around the TSS and TTS and measuring changes in prediction, we were able to show that the model associated motifs of the C2C2-GATA family with a positive influence on gene expression, but only if they were inserted downstream of the TSS, demonstrating that the model was able to learn biologically relevant positional dependencies that had been demonstrated experimentally (Voichek et al. 2024). This gives some credence to positional dependencies that the model has associated with other TF families. Most notably, the model predicted that TCP factors have a very strong positive position-dependent influence on gene expression. While TCP-binding CREs are highly enriched upstream of the TSS in close proximity to the core promoter, downstream binding of TCP factors is comparatively negligible (Hetzel et al. 2016; O’Malley et al. 2016) (Fig. 5a) and likely unfavorable due to competition with or steric hindrance of the core transcriptional machinery (Jores et al. 2021; Duttke et al. 2024). Additionally, the disruption of core promoter elements would be expected to negatively impact transcriptional activity (Chaturvedi et al. 2007; Ranjan et al. 2009; Srivastava et al. 2014b). This indicates that the model was only able to identify rough positional dependencies at a coarse-grained resolution, since, in general, insertions around TSSs were associated with an increase in expression. Nevertheless, several studies have highlighted the important role that TCP factors play in various aspects of gene regulation, such as promoter strength (Jores et al. 2021), the accessibility of chromatin (Schwope et al. 2021; Wrightsman et al. 2022) and even large-scale chromatin topology (Liu et al. 2017; Marand et al. 2023). TCP factors are involved in basic biological functions such as plant growth and development across many different tissues and developmental stages (Wang et al. 2025). The identification of TCP factors as important regulators under control conditions is therefore not surprising and provides further evidence for the model's ability to identify biologically relevant features. Interestingly, the model predicted bHLH and bZIP factors to have a positive effect on gene expression when binding upstream and around the TSS and a negative effect when binding downstream. DAP-Seq signals indicate that bHLH factors bind both upstream and downstream of the TSS in a bimodal pattern (Fig. 5a), and bZIP factors are known to often interact with bHLH factors in plants (Ezer et al. 2017). The human TFs NRF1 (a member of the mammalian bZIP family), YY1, and NFY have also been shown to act as activators or repressors, depending on the binding position relative to the TSS (Duttke et al. 2024).

As discussed above, single motifs are typically not predictive of gene expression outcomes; therefore, predicting the position-dependent influence of TF families by inserting single motifs comes with several limitations. For one, the consensus motifs of most TFs would be expected to randomly occur at a high frequency throughout the genome, but in vivo TF binding site selection is highly specific and typically context-dependent (de Martin et al. 2021). On the other hand, some TF families are much more diverse than others, not only in terms of recognized binding motifs, but also in terms of their influence on gene expression. Here, we only show median effects within families; however, individual members may act as positive regulators while others act as negative regulators. While some TFs may be less reliant on specific motifs and more reliant on DNA shape or co-regulating factors, other TFs may bind to very specific motifs, but their function depends on other co-regulators (de Martin et al. 2021). The interpretation is especially difficult for genes that are related to stress responses, since they would often be expected to be lowly expressed under control conditions. Therefore, the motifs of TF families associated with stress responses may also be associated with low expression by the model, even if the members of the respective families primarily function as activators. Since the model was trained to predict median gene expression, it is unsurprising that the dispersion of prediction errors was lowest for genes with low tissue-specificity (Figure S27c and d). Tissue- or condition-specific control of gene expression often depends on more complex regulatory mechanisms involving distal or epigenetic factors (Yan et al. 2024), which may be difficult for this type of model architecture to capture. The comparatively low model performance when predicting maximum gene expression (Fig. 2a) may suggest context-dependent activation of genes by mechanisms that are not captured by the model. Despite these limitations, we found that the errors made by the model can serve as a valuable source of information for identifying genes under complex regulation. We found that silenced genes tended to be overpredicted, whereas enhanced genes tended to be underpredicted (Fig. 7a and b). The magnitude and directionality of errors made by the model can therefore give useful hints toward the mechanisms underlying the regulation of any given gene.

## Conclusion

Highly optimized sequence-to-expression models are useful and versatile tools in the field of regulatory genomics. They can be used to independently validate and complement experimental findings and generate new hypotheses. We demonstrated that CNNs can not only recognize relevant motifs, but are also able to capture the spatial context of motifs in relation to their function. We were able to show that genes that may be subject to more complex regulatory mechanisms can be identified by comparing model predictions with observed expression. Having successfully trained a state-of-the-art model on the allotetraploid *B. napus* genome, we hope to lay the foundation for future studies of deep learning in complex crop species, which may be used to elucidate condition- or tissue-specific gene regulation and uncover the genetic and epigenetic factors underlying phenotypic variation and trait determination. The availability of robust species-specific models that can be probed and used to test the effects of mutations at low cost is invaluable for precision genome editing efforts. Far from being impenetrable black boxes, we have demonstrated their usefulness for generating hypotheses and gaining novel insight into gene regulation. In the future, we anticipate such models to aid in the creation of the next generation of resilient crop varieties that are capable of ensuring global food security.

## Methods

### Genomic data

The *B. napus* Express617 v1 assembly (Lee et al. 2020) was used as the genomic reference. A structural genome annotation containing UTR information was generated using Helixer (Stiehler et al. 2020; Holst et al. 2026) with the land plant v0.3 a 0100 model. Gene models were filtered using Gffread v0.12.7 (Pertea and Pertea 2020) with the options -CJV to retain only protein-coding genes with a valid open reading frame. Genes located on unplaced scaffolds were excluded from the dataset.

For *A. thaliana*, nuclear chromosomes of the TAIR10 genome assembly (Lamesch et al. 2012) were used together with the TAIR10 genome annotation, which was likewise filtered for protein-coding genes.

### Gene expression data

For *B. napus*, a total of 36 RNA-Seq samples from the cultivar Express617 with 1 to 3 biological replicates each, covering 8 tissues at various developmental stages, were used to quantify gene expression (Table S1). The RNA-Seq data included samples from 3 studies (Calderwood et al. 2021; Woolfenden et al. 2025; Zanini et al. 2025), as well as previously unpublished samples (PRJEB107092).

The reference genome was divided into subgenome-specific references containing only chromosome-scale scaffolds of the respective subgenome. Adapters were trimmed from RNA-Seq reads using trimmomatic v0.39 (Bolger et al. 2014). Trimmed reads were then aligned to each subgenome using STAR v2.7.10b (Dobin et al. 2012) with options —outSAMstrandField intronMotif, —outFilterIntronMotifs RemoveNoncanonicalUnannotated, —outSJfilterCountUniqueMin 3 2 2 2, —outMultimapperOrder Random, and —outFilterType BySJout. The resulting BAM files were further processed using the EAGLE-RC pipeline (Kuo et al. 2020) to obtain the most likely mapping locations, using Gffread v0.12.7 (Pertea and Pertea 2020) to extract transcript sequences, LAST v1447 (Kiełbasa et al. 2011) to call subgenome-specific variants, and EAGLE v1.1.3 (Kuo et al. 2018) for subgenome-specific read assignment. Transcripts per million (TPM) were estimated for each gene using Stringtie v2.2.1 (Pertea et al. 2015). Expression values were averaged across biological replicates within each sample before calculating the median expression across all 36 samples. Nonexpressed genes, which had a maximum expression of 0 across samples, were excluded from the dataset. Likewise, highly similar homoeologs of *B. napus* (100% identical coding sequence) were removed due to difficulties in obtaining accurate gene expression values, since RNA-Seq reads that cannot be unambiguously assigned to a specific homoeolog are discarded in the EAGLE-RC pipeline.

For *A. thaliana*, a precomputed TPM-normalized gene-level expression matrix for the Araport11 genome annotation (Cheng et al. 2017) , containing data from 56 RNA-Seq experiments covering various tissues, developmental stages, and growth conditions, was obtained from the EMBL-EBI Expression Atlas (https://www.ebi.ac.uk/gxa/experiments/E-CURD-1/Downloads) (Moreno et al. 2021). TAIR10 gene models were used instead of Araport11 gene models, as TAIR10 had been used to calculate TF binding-site distributions with respect to the TSS and TTS by Voichek et al (2024), and TAIR10 transcripts have substantially smaller UTRs on average. Median expression values were calculated across 54 of 56 samples. Two samples were discarded due to low Spearman correlation with the other samples (Figure S3). Nonexpressed genes (max. TPM of zero across the 54 included samples) were discarded.

### Plant growth, RNA extraction, and sequencing

#### Time series data (leaf, apex, floral organs)

*B. napus* cv. Express617 was sown in cereals mix (40% medium grade peat, 40% sterilized soil, 20% horticultural grit, 1.3 kg·m^−3^ PG mix 14-16-18 + Te base fertilizer, 1 kg·m^−3^ Osmocote Mini 16-8-11 2 mg + Te 0.02% B, wetting agent, 3 kg·m^−3^ maglime, 300 g·m^−3^ Exemptor). Material was grown in a Conviron MTPS 144 controlled environment room with Valoya NS1 LED lighting (250 μmol·m^−2^·s^−1^), 18 °C:15 °C, day:night, 70% relative humidity with a 16 h/d. At Day 21, plants were placed into vernalizing conditions at 5 °C (8 h/d) for 6 weeks before return to warm, long-day conditions. Plants were sampled at Day 7 (seedling), Day 21 (3 leaf stage, pre-cold treatment), Day 42 (4 to 5 leaf stage, 3 weeks mid-vernalization treatment), Day 63 (5 leaf stage, 6 weeks vernalization treatment), Day 64 (5 leaf stage, 1 d returned to warm, long day), Day 71 (eighth to ninth true leaf, apex vegetative) and Day 98 (BBCH51, floral buds present). At each sampling timepoint, 3 replicate samples containing either 3 whole seedlings (Day 7) or 3 dissected apices were collected. One leaf sample was collected at Days 64, 71, and 98. Sampling of bud and anther stages was performed at 131 d post-sowing. Three replicate samples of 3 buds from a single inflorescence were collected at Sanders stages 6 to 7 (Pollen Mother Cell (PMC) meiosis and tetrad stage), Sanders stages 10 to 11 (mitosis, polarized microspore to bicellular stage). Three replicates of anthers and gynoecia were collected at Sanders stages 12 to 13, comprising the largest 2 unopened buds and the next opening flower per inflorescence for 2 inflorescences per replicate (36 anthers and 6 gynoecia in total per replicate). Samples were ground in LN_2_ to a fine powder before RNA extraction and DNase treatment were performed following the method provided with the EZNA Plant RNA Kit (Omega Bio-tek Inc., http://omegabiotek.com/store/). RNA samples were processed at Novogene (Beijing, China). Complementary DNA (cDNA) libraries were constructed using NEBNext Ultra Directional Library Kit (New England Biolabs Inc., Ipswich, MA, United States). Sequencing was performed using Illumina NovaSeq 6000 for apex and leaf, and Illumina HiSeq X for bud, anther, and gynoecia, resulting in 150 bp paired-end reads.

#### Bulk tissue data (leaf, root, seedling, silique, immature flower bud)

*B. napus* cv. Express617 was grown under long-day conditions in growth chambers and vernalized for 10 weeks at a constant 5 °C. Five sample types were selected to represent 3 vegetative and 2 reproductive growth stages: leaves, roots, seedlings, immature flower buds, and siliques. RNA was extracted with Quick-RNA Plant Miniprep (Zymo Research), quantity and purity were assessed with Qubit RNA BR Assay Kit and Nanodrop (ThermoFisher Scientific), and RNA integrity with the RNA 6000 Nano Kit for 2100 Bioanalyzer Systems (Agilent). Samples were sent to Novogene for library preparation and sequencing with strand-specific Illumina 150PE. For further information, see Zanini et al. (2025).

### DNA affinity purification sequencing data analysis

DNA affinity purification sequencing (DAP-Seq) narrow peaks for various TFs of 15 TF families (MYB-related, Homeobox, C2C2-Dof, WRKY, TCP, bZIP, MYB, MADS, bHLH, Trihelix, NAC, G2-like, HSF, AP2/EREBP, C2C2-GATA) were obtained from NCBI GEO (Barrett et al. 2012; O’Malley et al. 2016). These 15 families are among the largest TF families (at least 10 members) and were shown to have strong positional binding preferences with respect to the TSS (O’Malley et al. 2016; Voichek et al. 2024). Peak coverages along the *A. thaliana* TAIR 10 reference assembly were calculated using the coverage tool of Bedtools v2.30.0 (Quinlan and Hall 2010) with the -d option and summed across TFs of each respective family. For each TF family, peak coverages within promoter regions (TSS ± 500 bp) and terminator regions (TTS ± 500 bp) were summed across all primary TAIR 10 transcripts. Orthologs in *B. napus* were identified based on protein similarity using BLASTp v2.12.0 (Camacho et al. 2009).

### Identification of genes associated with SEs and H3K27-trimethylation

SE-associated genes in *B. napus* were identified using SE locations provided by Zanini et al. (2025) and applying the function bedtools closest (v2.30.0). A set of Polycomb-silenced genes in *A. thaliana*, which are associated with H3K27me3 histone marks, was taken from the literature (Borg et al. 2020). Orthologs in *B. napus* were identified based on protein similarity using BLASTp 2.12.0 (Camacho et al. 2009).

### Data preprocessing

#### Partitioning of genes based on CDS similarity

Genes were partitioned into 10 evolutionarily independent sets of roughly equal size using GraphPart v1.0.2 (Teufel et al. 2023), which implements gene family-guided partitioning (Washburn et al. 2019). Genes were classified into one of 3 classes based on their expression levels, using the upper and lower quartiles as boundaries to categorize genes as lowly, moderately, or highly expressed. These classes were used to guide partitioning, ensuring that the distribution of expression levels was roughly equal between data folds. GraphPart uses sequence similarity between genes to determine their optimal partitioning to reduce evolutionary dependencies between sets, which could otherwise lead to overinflated performance estimations due to the model being able to overfit to evolutionary artifacts (Washburn et al. 2019). Partitioning was based on CDS similarity using GraphPart's mmseqs2needle function and the options -th 0.3 -re 0.2 -nu -tr.

For hyperparameter optimization and model performance evaluation, *B. napus* genes were partitioned by themselves. For cross-species comparisons of model performance and the calculation of attribution scores, *B. napus* and *A. thaliana* genes were partitioned jointly, using 6 classes based on all possible combinations of expression level and organism of origin to guide partitioning, so that proportional numbers of genes for each expression level and organism landed in each joint data fold. Afterward, genes were split back up, based on the respective organism of origin, resulting in 2 sets of data folds, one for each organism, which corresponded evolutionarily between organisms.

#### Sequence data

CDS features were hard-masked from the reference assemblies using the maskfasta tool from Bedtools v2.31.1. This was done to further mitigate potential overfitting due to spurious correlations between homologs that might have been missed by GraphPart.

For each gene, the TSS and TTS were identified as the outer boundaries of the outermost annotated exons of the longest annotated isoform. Sequence intervals ranging from −15 to +5 kb around the TSS and −5 to +15 kb around the TTS were extracted from the respective reference assembly. Sequence intervals that extended beyond the outer boundaries of the respective chromosome were padded with Ns. For genes annotated on the minus strand, the reverse complement sequence was extracted accordingly.

#### Xpresso gene features

To train the Xpresso model (Agarwal and Shendure 2020), 8 additional features (lengths and GC contents of 5′ UTRs, 3′ UTRs, and total CDS, the total intron length, and the number of CDS-exons per kilobase of CDS sequence) were extracted from the reference assembly and genome annotation.

#### Data scaling

The median expression data, as well as all the numeric input features for the Xpresso model, except for GC-content, were log_10_ transformed after adding an offset of 0.1.

For hyperparameter optimization and performance evaluation, 2 data folds were reserved as test and validation sets. In order to prevent information leakage, a standard scaler was fit only to the numeric data in the 8 remaining data folds, which served as the training set, before applying it to all data folds. For cross-species comparisons of model performance and the calculation of attribution scores, each data fold was held out once as a test fold, while training a model instance on the remaining 9 data folds, respectively. This strategy resembles a 10-fold cross-validation scheme, as is often used in machine learning (Raschka 2018). This was possible because the training regimen was fixed to 6 epochs on a fixed learning rate schedule, which obviated the need for a separate validation set that would typically be used to stop model training based on the validation loss. Accordingly, standard scalers were fitted to the corresponding 9 training folds, preventing data leakage to the respective test fold. For the final predictive model, a standard scaler was fitted to the entire dataset.

### Hyperparameter optimization

Hyperparameters were optimized in a multistep process using Bayesian optimization over large search spaces with Optuna v3.6.0 (Akiba et al. 2019). In the first set of steps, the hyperparameters that govern the general model architecture were tuned using validation loss (mean squared error) as the performance metric. In subsequent steps, the hyperparameters that govern the learning algorithm itself, including the loss function, were optimized using the coefficient of determination (*r*^2^), on the validation set as the performance metric. Hyperparameters were optimized with respect to a single data-fold configuration, setting fold 0 aside as the test set and not using it at all during hyperparameter optimization. Fold 1 was used as the validation set to evaluate model performance for different hyperparameter settings. The remaining 8 folds were used to train the model in each round of optimization.

1. As a first step, the hyperparameters governing the overall architecture of the model were optimized using the Adam optimizer (Kingma and Ba 2014) with default parameters and selecting hyperparameters that minimize the mean squared error on the validation set. The hyperparameter search space for the model architecture (Table S2) was mostly a superset of published architectures that were used successfully for similar tasks (Quang and Xie 2016; Washburn et al. 2019; Agarwal and Shendure 2020). In addition, the search space included convolutional strides larger than 1, overlapping pooling windows, average-pooling, and a large array of activation functions. Overlapping pooling windows (pooling strides smaller than the window size) were used in an effort to improve the ability of the network to resolve the spatial organization of motifs by increasing the overlap of receptive fields, thus mitigating the occurrence of multifaceted neurons (Koo and Eddy 2019; Wei et al. 2023). In the case of image classification, the use of overlapping pooling windows has also been reported to increase performance and reduce overfitting (Krizhevsky et al. 2017; Galanis et al. 2022). Features from promoter and terminator sequences were extracted in separate convolutional branches, so that convolutional kernel sizes could be optimized independently during hyperparameter tuning and the corresponding model weights could be optimized independently during training. This was done to allow for some flexibility between branches, since relevant features may differ between promoter and terminator sequences. However, this flexibility comes at the cost of an increased number of model parameters. Pooling window sizes, pooling strides, and the number of convolutional filters in the last convolutional layer were kept equal between both branches to ensure that their respective output feature spaces have equal dimensions.
2. A selection of hyperparameters found in the first round of optimization, related to the convolutional layers, was validated by switching hyperparameter values between the 2 convolutional branches or using default values instead (Table S3). The hyperparameters that had been found for the promoter and terminator branches, respectively, were tested in all possible combinations, using them for either or both branches. The best performance was seen when the hyperparameters of the promoter branch were used for both branches. The type of pooling layer had been optimized in the first round between max-pooling and average-pooling for each individual pooling layer. To validate this decision, the optimized configuration of pooling types was tested against using only max-pooling or only average-pooling. The best performance was seen when using only average-pooling, instead of the optimized configuration. The use of convolutional strides larger than one and overlapping pooling windows was tested against the respective default. The best performance was seen when using default strides for the convolutional operations and optimized strides (smaller than or equal to the pooling window size) for the pooling operations. The architecture validation was performed by grid search, testing all possible permutations of the above-mentioned aspects.
3. The model architecture was then fine-tuned, using insights from the first 2 rounds as guidance to narrow down the search space (Table S4).
4. Next, the training algorithm and the loss function were optimized, keeping the model architecture constant (Table S5). Instead of using Adam with default parameters, in this step, Nadam (Dozat 2016), a variation of the Adam optimizer that uses Nesterov momentum, was used, treating the function parameters as hyperparameters to optimize. Instead of using a fixed learning rate, at this stage, a one-cycle learning rate schedule was implemented, consisting of a gradual warm-up phase, followed by a high peak learning rate and a subsequent cool-down phase, allowing for fast and consistent model convergence that generalizes well by smoothing out the loss landscape and stabilizing gradient descent (Smith and Topin 2019; Kalra and Barkeshli 2024). The length and shape of the cycle, as well as the initial, final, and peak learning rate, were optimized. The momentum was cycled similarly, but in the opposite direction to the learning rate, optimizing the maximum and minimum momentum (Figure S4a). High peak learning rates provide a regularizing effect (Smith 2018), which was balanced by setting the batch size to a value of 130, which was the highest possible batch size, given the memory constraints of the GPU. Weight decay (Krogh and Hertz 1991) and dropout rates (Srivastava et al. 2014a) were optimized together with the learning rate schedule (Smith 2018) in order to find an optimal combination of regularizing influences. The loss function itself was also optimized, using Huber loss instead of mean squared error and optimizing its delta parameter. Since loss values could not be directly compared between trials, the coefficient of determination (*r*^2^) on the validation set was maximized. The tuning of the Nadam optimizer and the corresponding hyperparameter search space was inspired by Choi et al. (2019), who argued that more sophisticated optimizers should always perform better or equally as well as less sophisticated optimizers, given adequate parameter settings.
5. Finally, the model training was further fine-tuned in 2 final stages, progressively reducing the hyperparameter search spaces based on results from previous stages (Tables S6 and S7), in order to arrive at the final set of hyperparameters (Table S8, Fig. 1, Figure S1).
  When applying the hyperparameters of the promoter branch to both branches in the final architecture, we saw a decrease in performance, confirming that the branch-specific hyperparameters provided an advantage.

### Model training and evaluation

Before training a given model instance, the necessary data folds were loaded into memory, and numeric data were scaled using the appropriate standard scaler. The training folds were concatenated to form the training set. The order of samples within each dataset was randomly shuffled, and sequences were one-hot encoded.

The models were trained on NVIDIA V100 GPUs with 32 GB of VRAM and implemented using the functional API of TensorFlow v2.14.0 (Abadi et al. 2016). All previously published models used for comparison were reimplemented in TensorFlow v2.14.0 and trained on the same data as *n*emo.

#### Training for performance evaluation

For the purpose of evaluating *n*emo against 5 competing architectures for gene expression prediction from proximal regulatory sequences, all models were trained on the same training set used during hyperparameter optimization, encompassing 80% of the entire dataset. The same validation set that had been used to assess model performance during hyperparameter optimization was used to monitor validation loss during training. Instances of *n*emo that were trained on 80% of the data are referred to as *n*emo_80_. All models were trained 10 times on the same training data, due to the stochastic nature of the model training procedure, to capture the variability in performance across training runs (Fig. 2a).

The Xpresso model was trained using the Adam optimizer with parameters learning_rate = 0.001, beta_1 = 0.9, beta_2 = 0.999, epsilon = 10^−8^ and weight decay = 0.0 and mean squared error as the loss function. Early stopping was used with a patience of 10 epochs. The model weights that had achieved the lowest validation loss were saved. The original Xpresso model architecture (Agarwal and Shendure 2020) had to be slightly altered due to issues with model convergence, likely caused by the “dying ReLU” phenomenon (Lu et al. 2020). The authors of the original paper (Agarwal and Shendure 2020) had reported achieving convergence in 9 out of 10 training runs. For our data, the rate of convergence was much lower. Using the leaky ReLU activation function (Maas et al. 2013) with a slope of 0.1 solved the convergence issues. In addition, the TensorFlow callback function ReduceLROnPlateau was used to further stabilize convergence. The input sequence interval for the Xpresso model spanned 7 kb upstream of the TSS to 3.5 kb downstream of the TSS. To train Xpresso without halflife features, the exact same training procedure was applied to the same architecture, except that halflife features were not concatenated to the output features of the convolutional stack.

For both PhytoExpr models, ReLU activation was replaced by leaky ReLU with a slope of 0.1, for the same reason as for the Xpresso model. The output activation was changed to linear, since standard scaling was applied to the expression data that were used in this study, meaning that values were centered around zero with a standard deviation of one. ReLU activation in the output layer would not have allowed the model to predict “negative” expression values. The submodels of the PhytoExpr-ensemble were trained using the Adam optimizer with default parameters, whereas the transformer model was trained using a learning rate of 0.0001, as was used in the original implementation. Mean squared error was used as the loss function. As in the original implementation, early stopping with a patience of 2 was used to stop training based on the validation loss. The input interval of the PhytoExpr-transformer model was a concatenated sequence spanning 4 kb upstream of the TSS to 1 kb downstream of the TSS and 1 kb upstream of the TTS to 4 kb downstream of the TTS. The submodels of the CNN ensemble used inputs of varying lengths. The inputs spanned 2 to 4 kb upstream of the TSS to 1 kb downstream of the TSS and 1 kb upstream of the TTS to 2 to 4 kb downstream of the TTS.

Basenji-5K, which is based on Basenji-3K as implemented by Qiu et al. (2025), takes a sequence interval of ± 5 kb around the TSS as its input, since this is the optimal sequence interval identified for *A. thaliana* by the authors. As in the original implementation, it was trained using the Adam optimizer with default parameters and a learning rate schedule that uses a learning rate of 10^−3^ for the first 3 epochs and 10^−5^ thereafter. Mean squared error was used as the loss function. As in the original implementation, early stopping with a patience of 5 was used to stop training based on the validation loss. The input sequence for the Basenji-5K model consisted of the interval from 5 kb upstream of the TSS to 5 kb downstream of the TSS.

To train *n*emo, the legacy Nadam optimizer of TensorFlow v2.14.0 with parameters beta_2 = 0.9982, epsilon = 1.1330 · 10^−6^, weight_decay = 3.2094 · 10^−5^ was used to minimize Huber loss with delta = 2.9466. The learning rate and momentum (beta_1) were scheduled using a one-cycle policy (Smith and Topin 2019; Smith 2018; Tananaev-Kort 2021) that reliably achieved convergence within a fixed training regimen of 6 epochs. The validation loss was monitored but was not used to stop training. Since the validation set was not needed to stop the training of the *n*emo model, another set of 10 model instances was created by incorporating the validation set into the training set and training the model on 90% of the data, referred to as *n*emo_90_. Importantly, for the purpose of general performance evaluation (Fig. 2a) these instances were only trained on 90% of the *B. napus* data used during hyperparameter optimization and tested on the same unseen test set as *n*emo_80_.

#### Training for model interpretation and cross-species comparisons

Using data folds derived from the joint partitioning of *A. thaliana* and *B. napus* genes, 10 *n*emo_90_ instances were trained for each species. Each model instance was trained on a different training set, holding out one of the data folds as a test set to calculate attributions on, and training on the remaining 9 data folds, similar to 10-fold cross-validation (Raschka 2018). Importantly, the respective test sets remained separate from the training set of each model instance, enabling minimally biased predictions and attributions for the entire dataset when combining the predictions of all 10 model instances on their respective test sets. Due to the joint partitioning of data between species, corresponding data folds contained homologous gene families (eg fold 0 of *A. thaliana* contained gene families related to genes in fold 0 of *B. napus*, but practically unrelated to genes in all other data folds); therefore, corresponding test sets could be used interchangeably between species. Apart from the cross-species comparison shown in Fig. 2b, models were always trained and tested on the same organism.

#### Training a final predictive model

A final instance of *n*emo was trained on the entire dataset to release the model weights as a tool to make predictions on other genotypes and other *Brassica* crops. This model instance, referred to as *n*emo_100_, was used to validate the model on real-world data.

#### Model predictions

For each trained model instance, predictions were made only on held-out test data, complementary to the data on which the respective model instance was trained. The test set used to evaluate general model performance (Fig. 2a) was not used at any point during hyperparameter optimization or model training and contained gene families never before seen by the model. To assess the contributions of promoters and terminators to the total prediction, the same *n*emo_80_ instances used for general performance evaluation were used to make predictions on mutated versions of the test set genes, where the up- or downstream regions around the TSS or TTS were replaced by random sequences, respectively. For calculating attributions and cross-species performance comparisons based on *n*emo_90_, every data fold was held out from training once to obtain minimally biased predictions for every gene in the entire set. Furthermore, for the cross-species comparison, the *B. napus* test sets were randomly down-sampled to match the size of the *A. thaliana* test sets for better comparability; however, the training sets were not down-sampled.

Genes were defined as well-predicted if the absolute error (|*ε*|) was lower or equal to the median absolute error across all genes (Fig. 7c). Accordingly, genes for which the signed error fell above or below the range of well-predicted genes were considered over-predicted or under-predicted, respectively (Fig. 7a).

### Attribution

Attribution scores for the input sequences with respect to the model output were calculated using the GradientExplainer from SHAP v0.42.0 (Lundberg and Lee 2017). This explainer implements the expected gradients algorithm (Erion et al. 2021), which is an extension of the integrated gradients algorithm (Sundararajan et al. 2017). It calculates robust attribution scores by sampling multiple reference sequences instead of using a single reference. For each input sequence, a set of 10 reference sequences was generated by (i) splitting the sequence into fragments that contained nucleotide information and noninformative (masked) fragments that contained only Ns. (ii) Sequence fragments containing nucleotide information were then shuffled 10 times using the dinuc shuffle function from DeepLIFT v0.6.13.0 (Shrikumar et al. 2017). The masked sequence fragments were copied 10 times and (iii) concatenated back together with the shuffled fragments (Figure S29). This approach made sure that the references stayed true to actual input sequences by containing contiguous stretches of shuffled nucleotides, interspersed with masked stretches that were unimportant by design. In the masked regions, all 10 references were identical to the respective input; therefore, the resulting attribution was automatically zero in those regions. Since the model exhibits some degree of overfitting (higher performance on the training set compared to the validation set [Figure S4b]), all attribution maps were calculated based on predictions made on the respective test set. For each test set, a separate *n*emo_90_ instance had been trained and used to calculate the attributions for the respective held-out test set. Attributions for all 10 test sets were then concatenated together to arrive at a complete set of minimally biased attributions for each gene in the dataset.

### Global and local importance scores

The sum of absolute attribution values across the 4 bases was used as a measure of local importance at a given sequence position and gene (Eq. 1). For a group of genes, the local importance scores were averaged at each position to give group-specific importance scores (Eq. 2). Global importance scores across all genes (Eq. 3) were used as a background reference value for normalization, together with the global standard deviation (Eq. 4).

Since coding sequences had been masked and attribution scores were set to zero by design in the respective regions, the number of genes in which a given sequence position was masked was accounted for when calculating averages and standard deviations of local importance scores across genes. This was done to minimize the effect that masking had on the resulting scores. Only well-predicted genes, for which the absolute prediction error was smaller than or equal to the median absolute prediction error, were used to calculate group-specific importance scores. Since attributions had only been calculated for unseen test data, it was assumed that selecting only well-predicted genes increased the likelihood that nonzero importance scores would coincide with generalizable patterns instead of artifacts from overfitting on the training set. The global importance scores, on the other hand, were calculated across all genes, including those that had been poorly predicted.


(1) $$\lambda_{g,p}=\sum_{b=1}^{4}\left|A_{g,p,b}\right|$$

(2) $$I_{\gamma ,p}=\frac{1}{n_{\gamma }}\cdot \sum_{g\in \gamma }\lambda_{g,p}\;\text{where}\;n_{\gamma }=\sum_{g\in \gamma }\sum_{b=1}^{4}S_{g,p,b}$$

(3) $$\mu_{p}=\frac{1}{n}\cdot \sum_{g\in G}\lambda_{g,p}\;\text{where}\;n=\sum_{g\in G}\sum_{b=1}^{4}S_{g,p,b}$$

(4) $$\sigma_{p}=\sqrt{\frac{1}{n}\cdot \;(\sum_{g\in G}{\left(\lambda_{g,p}-\mu_{p}\right)}^{2}+(N-n)\cdot \;\mu_{p}^{2})}$$

where *A_g_*_,*p*,*b*_ ∈ R is the Shapley value, measuring the attribution of base *b*, at position *p* of gene *g*, *S_g_*_,*p*,*b*_ ∈ {0, 1} is the corresponding value of the one-hot encoded input sequence and *λ_g_*_,*p*_ ∈ $R^{+}$ = {*x* ∈ R|*x* ≥ 0} is the local importance of gene *g* at position *p*. *I_γ_*_,*p*_ ∈ $R^{+}$ is the group-specific importance of position *p* for group *γ* ⊂ *G*. *G* is the set of all genes, with *g* ∈ *G* referring to any gene in the dataset. *µ_p_* ∈ $R^{+}$ is the global background importance calculated across all genes in the dataset and *σ_p_* is the global standard deviation of local importance scores at position *p* across all genes in the dataset. *N* is the total number of genes in the entire dataset, *n* is the total number of genes that are *not* masked at position *p*, while *n_γ_* is the number of genes in group *γ* that are *not* masked at position *p*. Note that *λ_g_*_,*p*_ is equal to zero at masked positions.

### Smoothing and normalization of importance scores

Due to the use of overlapping pooling windows, the input regions in which the overlap occurred were connected to a greater number of neurons in the deeper layers. Therefore, changes in these regions had a greater potential to have an impact on the output of the model. This inherent architectural bias is reflected in the importance scores as a periodic signal, with a period equal to the stride *s* of the respective pooling layer and, accordingly, a frequency equal to *s*^−1^. A Fourier analysis of the global background importance confirmed a large peak at the expected frequency for the first pooling layer, as well as some of its harmonic frequencies, but also showed that the effect of deeper pooling layers was negligible. Applying a Savitzky–Golay filter (Savitzky and Golay 1964) with a kernel width of 75 (5 times the period of the noise signal) and a polynomial order of 1 resulted in a smooth signal that was comparable to the signal that could be achieved by directly filtering the identified noise frequencies (Figure S30). In most figures, a window size of 101 was used for smoothing.

In order to highlight group-specific differences in the importance landscape (Fig. 5b and c), importance scores were normalized to make them more comparable between different regions in the input and different groups of genes. The normalization was done in a 4-step process (Figures S9 and S10). First, Savitzky–Golay filters were applied to the global importance, the global standard deviation of local importances, and the group-specific importances, as described above, using the R-package gsignal 0.37 (van Boxtel et al. 2021). Second, importance scores were mean-normalized by subtracting the mean importance across the promoter and terminator for each respective group. This was done due to the additivity of Shapley values (Lundberg and Lee 2017), which means that the attributions of a given sample add up to the difference between the predicted value and a reference value. Due to this property, the sum of the importance scores of a given gene is correlated with the predicted expression of that gene, which, depending on the context, leads to potential bias when comparing importance scores between groups of genes that may vary in their average expression. Third, group-specific residual importance scores were calculated by subtracting the mean-normalized global importance from the mean-normalized group-specific importances. This step highlights how the importance scores of each group differ from the global background importance. Lastly, the group-specific residual importance scores were divided by the global standard deviation of local importance scores in order to emphasize differences in areas with low variability and de-emphasize differences in areas with high variability.


(5) $$I_{\gamma ,p}^{*}=\frac{\left(\;\varphi (I_{\gamma ,p})-\frac{1}{L}\sum_{\;p=1}^{L}\varphi (I_{\gamma ,p})\right)-\left(\;\varphi (\mu_{p})-\frac{1}{L}\sum_{\;p=1}^{L}\varphi (\mu_{p})\right)}{\varphi (\sigma_{p})}$$

where $I_{\gamma ,p}^{*}\in R$ is the normalized importance of group *γ* at the sequence position *p*, *φ*(.) denotes the Savitzky–Golay filter and *L* = 12,400 is the combined length of the 2 input sequences.

### Analysis of TF-MoDISco seqlet enrichment

We used TF-MoDISco (Shrikumar et al. 2018) (tfmodisco-lite v2.2.1) to discover short sequence fragments associated with high positive or low negative attribution values (seqlets) and assemble them into predictive patterns (modisco motifs -w 100 -n 20,000) and then compare them to known motifs in the JASPAR-2024 database (Rauluseviciute et al. 2023) of plant TF motifs (modisco report –write-tomtom). Due to protein-coding regions being masked in the input sequences, leading to long stretches of zero attributions in the corresponding attribution matrices, we were unable to run TF-MoDISco on the entire input sequence; instead, we split the input sequences ±500 bp around the TSS and TTS into 100 bp regions that we analyzed one at a time, using only genes that were not masked within the respective region. For each of the 100 bp regions, we counted the number of seqlets associated with each of the 15 TF families, based on the best hit across the JASPAR database for the corresponding predictive pattern.

### In silico motif insertion analysis

Motifs were systematically inserted into moderately expressed genes from each of the 10 data folds to assess their position-dependent effects on model predictions. For each TF family, the position weight matrix of each motif of the respective family (from *A. thaliana* or *Arabidopsis lyrata*) was gathered from the JASPAR database (Rauluseviciute et al. 2023). If there were multiple versions of the motif, the latest version was chosen. For each data fold, a set of 10 motifs from each TF family was randomly sampled. The position weight matrices were converted into position probability matrices that were used to dynamically generate the sequences to insert. The 10 motifs of each TF family were evenly inserted into the ±500 bp sequence intervals around the TSS and TTS, respectively. Each motif was inserted at every 10th position with a unique offset between 0 and 9 to ensure complete and uniform coverage. At a given position, the sequence to insert the motif into was randomly selected from the subset of moderately expressed genes of a given data fold that were not masked at the respective position. Predictions were then made for all mutated sequences (each containing one motif insertion) using the *n*emo_90_ model instance that had not been trained on the respective data fold from which sequences were sampled. This procedure was repeated for all 10 data folds and both species, using the model instances that were trained on the respective species. In total, 10 motif insertions were made at each position for each TF family. Each of the 10 insertions per family per position was made into a different random wild-type sequence (one from each data fold). For each mutated sequence, a set of 3 reference sequences was created from the same underlying wild-type sequence by inserting control motifs at the same respective positions. The control motifs consisted of a reversed version of the TF-binding motif, a randomly scrambled version of the TF-binding motif, and a fully random motif with the same length as the corresponding TF-binding motif. Residual position-dependent effects of TF-binding motifs were calculated by subtracting predictions made on reference sequences containing scrambled and fully random motifs from predictions made on the corresponding test sequences containing the intact TF-binding motifs. Scrambled and fully random motifs were used to, respectively, control for effects based only on GC-content and effects that only depend on the position of insertion, irrespective of the motif. Baseline predictions made on the wild-type inputs were subtracted from the predictions made on each type of mutated sequence to assess the effect sizes of each type of insertion. The median change in predicted expression (with respect to the reference or wild-type sequence) was calculated for each TF family at each position. Median changes in expression were smoothed across positions by applying Savitzky–Golay filters of size 101 and polynomial order 1.

### Model validation with real-world data

Gene expression (RNA-Seq) data for young pre-vernalization leaves for Express617 and semi-winter oilseed rape ZS11 were obtained from public databases (Zhang et al. 2024b) and the BRAVO project from the John Innes Centre (JIC) (Woolfenden et al. 2025). To ensure maximum comparability across annotations, structural gene annotation was lifted from Express617 onto ZS11 using Liftoff v1.6.3 (Shumate and Salzberg 2021). Gene expression was predicted using *n*emo_100_. RNA-Seq data was preprocessed and quantified in the same manner as for model training. Genes were prefiltered to only keep genes where expression in one of the ecotypes was higher across all the samples. Differences in the predicted gene expression between Express617 and ZS11 were calculated, and genes were sorted by this value. Top 100 and top 1,000 genes where (Express 617 predicted expression) > (ZS11 predicted expression) and (ZS11 predicted expression) > (Express 617 predicted expression) were extracted, and actual observed levels of expression were compared. To detect SVs between the 2 ecotypes, the ZS11 genome (Song et al. 2020) was aligned to the Express617 genome v1 using minimap2 v2.24 (—ax asm5 —cs —r2k) (Li 2018). Then, SVs were called with svim-asm v1.0.2 (haploid —min_sv_size 50 —max_sv_size 50,000) (Heller and Vingron 2020). RegioneR (Gel et al. 2015) was used to test the over-representation of SVs in extended gene bodies (1 kb upstream and downstream).

## Supplementary Material

kiag473_Supplementary_Data

## Data Availability

The source code for this project is available at https://github.com/GoliczGenomeLab/nemo.
