# Supplementary material for: A deep learning model captures position-specific effects of plant regulatory sequences and suggests genes under complex regulation
Source: Plant Physiol. 2026 Jul 7;201(4):kiag473. doi: 10.1093/plphys/kiag473 (PMC13436691; doi:10.1093/plphys/kiag473)
Supplement: Supplementary file 1 [file kiag473_supplementary_data.zip › nemo_REVISION2_Supplementary_Figures_2.pdf]

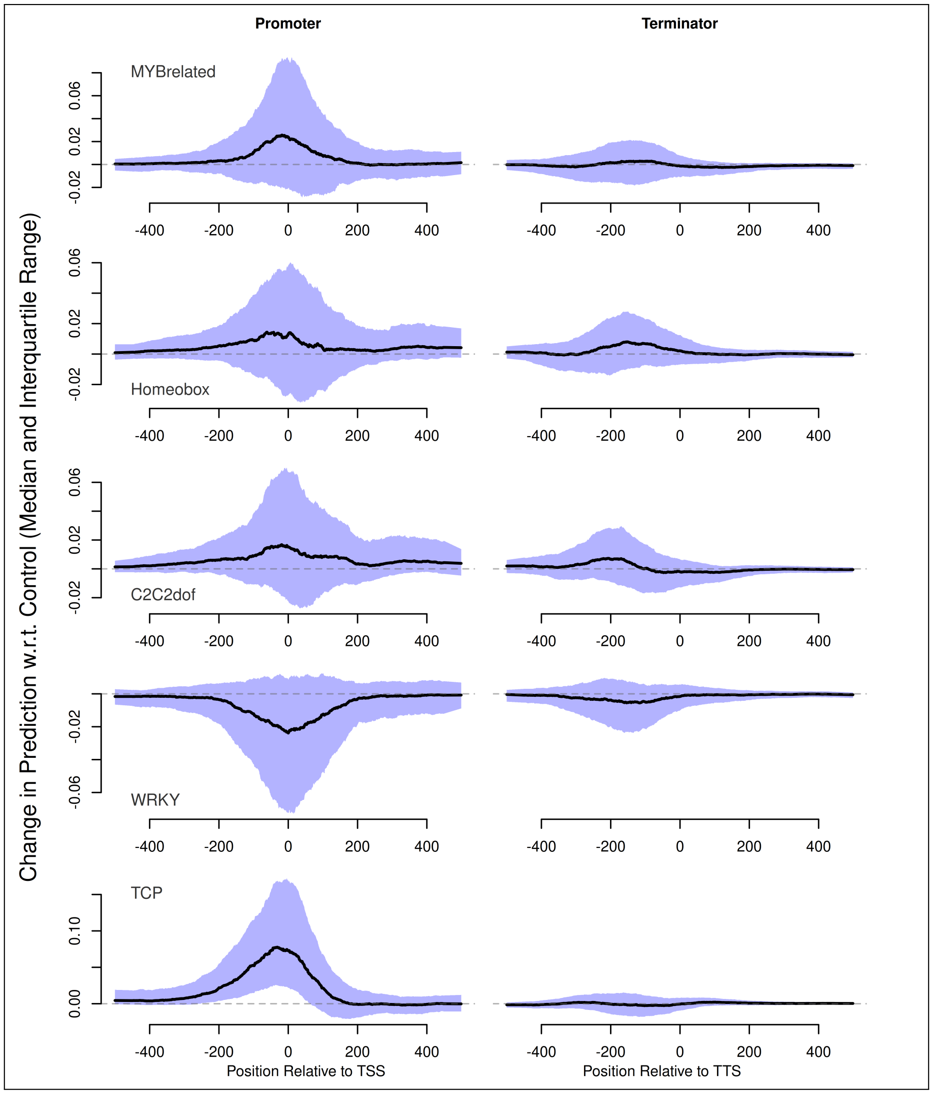

Fig. S18. Predicted changes in expression caused by insertion of TFs of the MYB-related, Homeobox, C2C2-Dof, WRKY and TCP families with respect to the insertion position relative to the TSS and TTS in *B. napus*. Expression changes were measured with respect to controls containing a random motif at the same position in the same sequence context. Black lines show the median change in expression at each position, while the shaded area shows the upper and lower quartiles. Dashed horizontal lines represent baseline expression. At each position 10 random TFs of the respective family were inserted into different genes. Values were smoothed using a 101 bp sliding window.

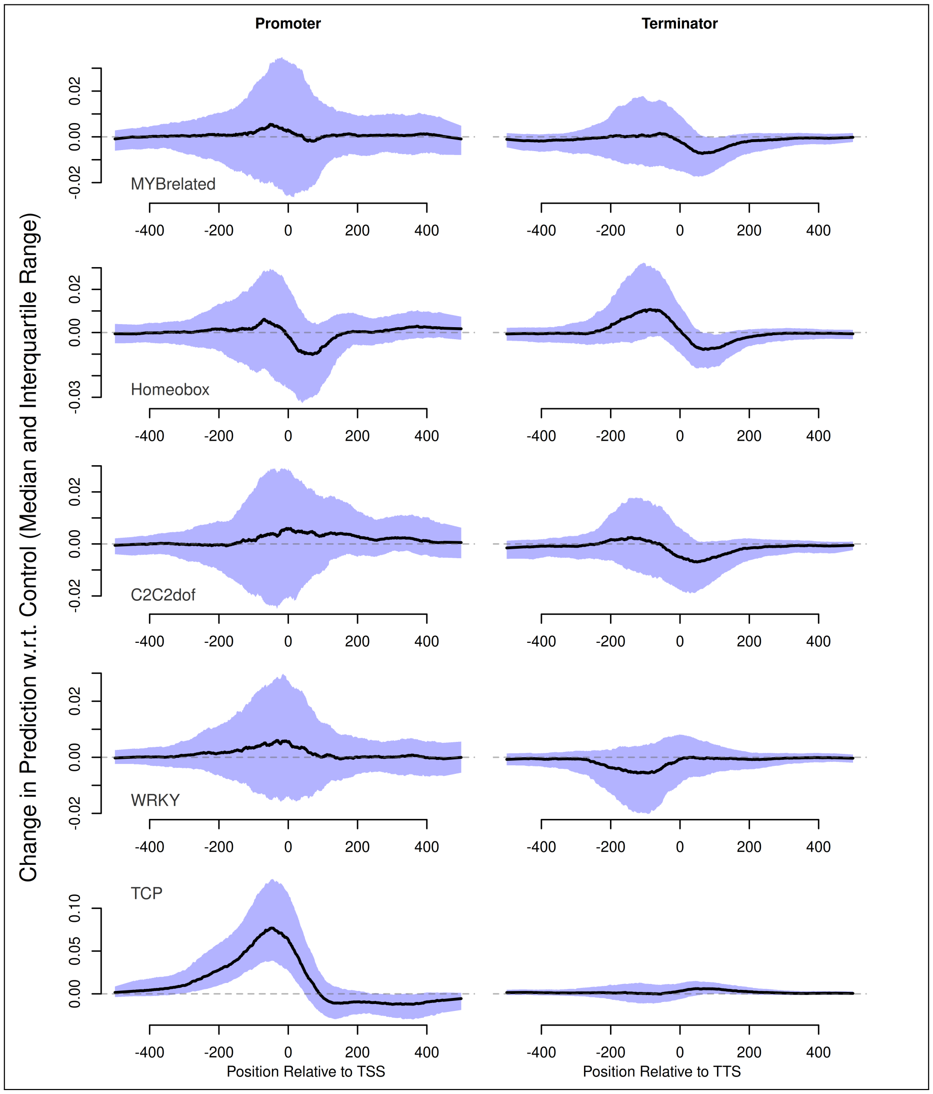

Fig. S19. Predicted changes in expression caused by insertion of TFs of the MYB-related, Homeobox, C2C2-Dof, WRKY and TCP families with respect to the insertion position relative to the TSS and TTS in *A. thaliana*. Expression changes were measured with respect to controls containing a random motif at the same position in the same sequence context. Black lines show the median change in expression at each position, while the shaded area shows the upper and lower quartiles. Dashed horizontal lines represent baseline expression. At each position 10 random TFs of the respective family were inserted into different genes. Values were smoothed using a 101 bp sliding window.

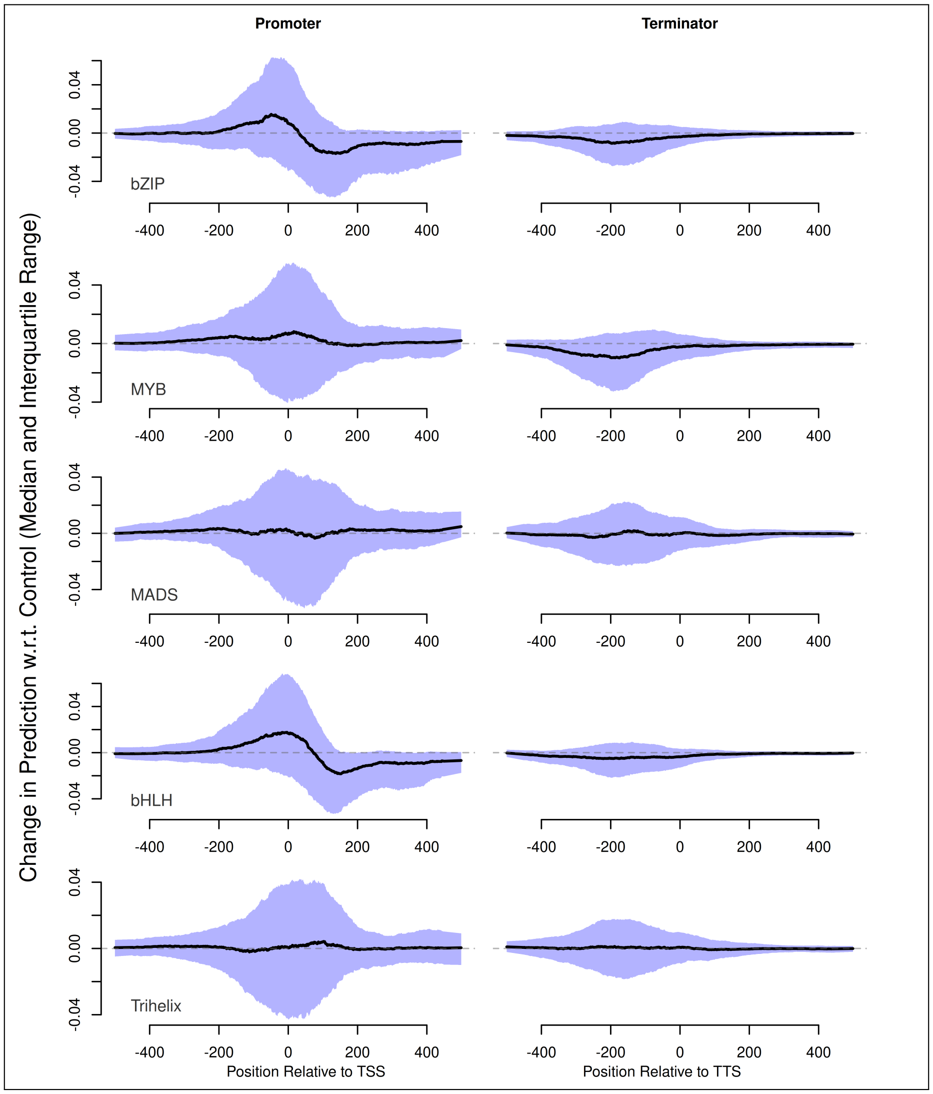

Fig. S20. Predicted changes in expression caused by insertion of TFs of the bZIP, MYB, MADS, bHLH and Trihelix families with respect to the insertion position relative to the TSS and TTS in *B. napus*. Expression changes were measured with respect to controls containing a random motif at the same position in the same sequence context. Black lines show the median change in expression at each position, while the shaded area shows the upper and lower quartiles. Dashed horizontal lines represent baseline expression. At each position 10 random TFs of the respective family were inserted into different genes. Values were smoothed using a 101 bp sliding window.

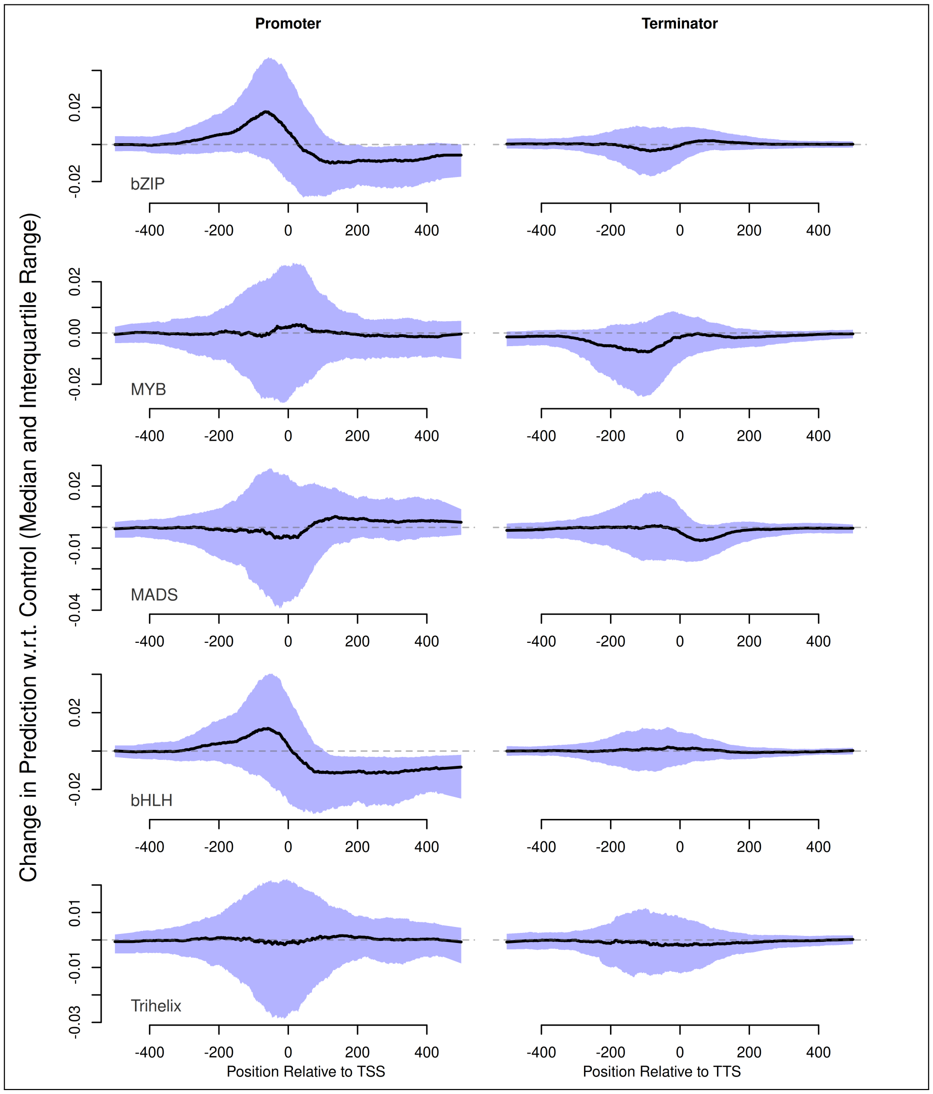

Fig. S21. Predicted changes in expression caused by insertion of TFs of the bZIP, MYB, MADS, bHLH and Trihelix families with respect to the insertion position relative to the TSS and TTS in *A. thaliana*. Expression changes were measured with respect to controls containing a random motif at the same position in the same sequence context. Black lines show the median change in expression at each position, while the shaded area shows the upper and lower quartiles. Dashed horizontal lines represent baseline expression. At each position 10 random TFs of the respective family were inserted into different genes. Values were smoothed using a 101 bp sliding window.

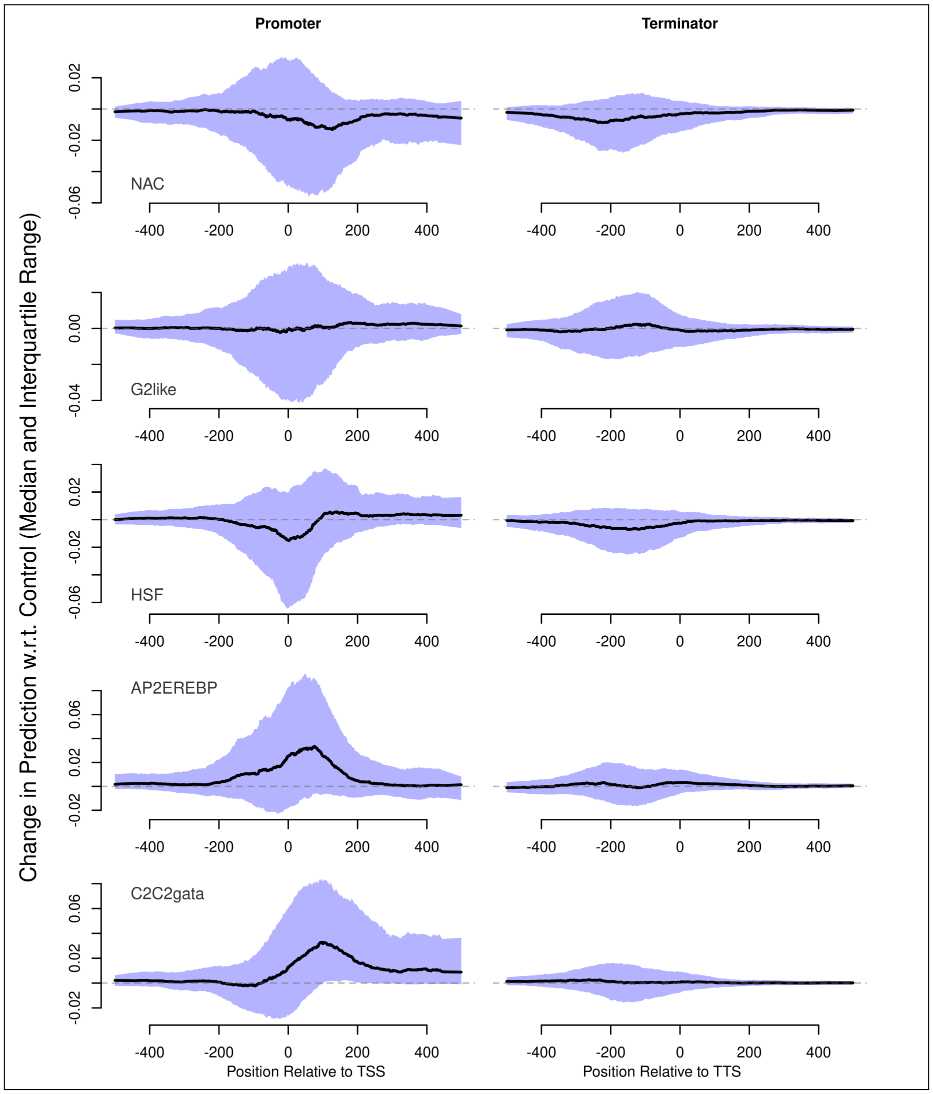

Fig. S22. Predicted changes in expression caused by insertion of TFs of the NAC, G2-like, HSF, AP2/EREBP and C2C2-GATA families with respect to the insertion position relative to the TSS and TTS in *B. napus*. Expression changes were measured with respect to controls containing a random motif at the same position in the same sequence context. Black lines show the median change in expression at each position, while the shaded area shows the upper and lower quartiles. Dashed horizontal lines represent baseline expression. At each position 10 random TFs of the respective family were inserted into different genes. Values were smoothed using a 101 bp sliding window.

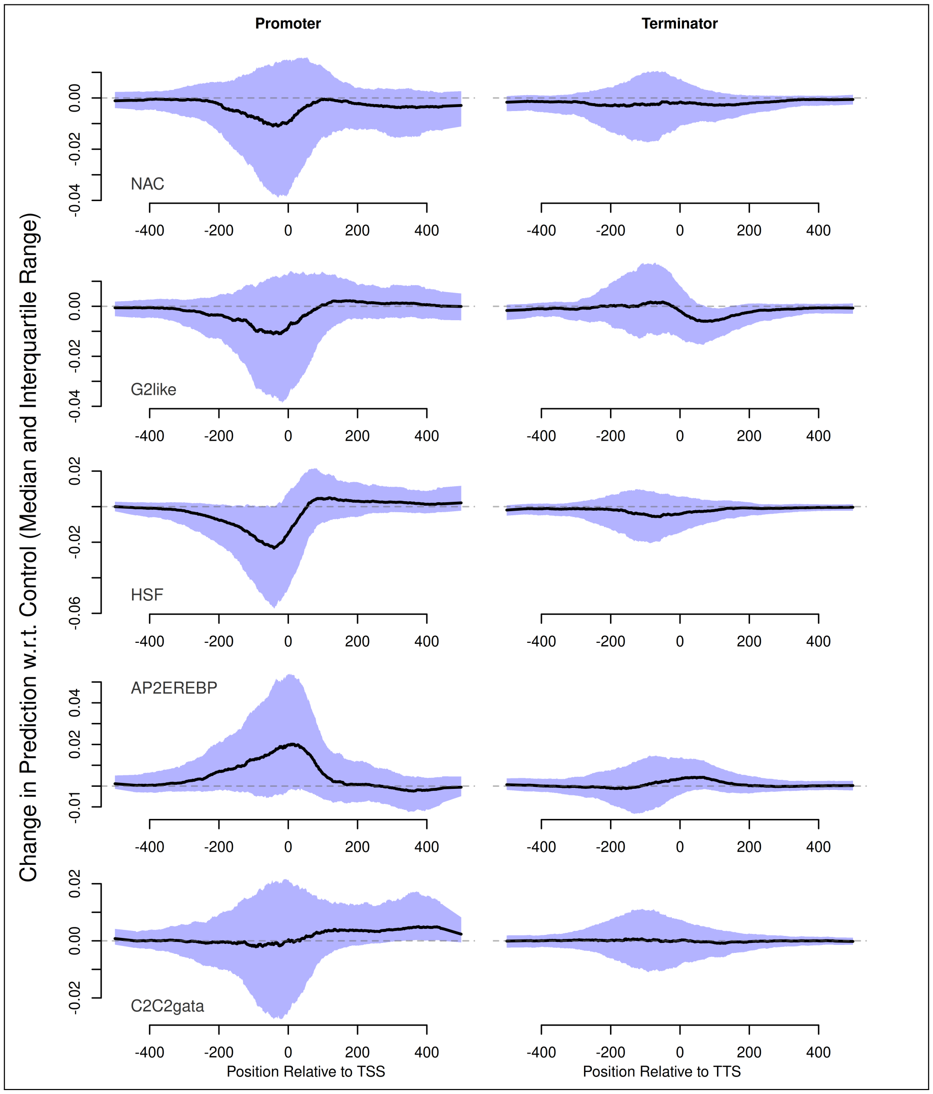

Fig. S23. Predicted changes in expression caused by insertion of TFs of the NAC, G2-like, HSF, AP2/EREBP and C2C2-GATA families with respect to the insertion position relative to the TSS and TTS in *A. thaliana*. Expression changes were measured with respect to controls containing a random motif at the same position in the same sequence context. Black lines show the median change in expression at each position, while the shaded area shows the upper and lower quartiles. Dashed horizontal lines represent baseline expression. At each position 10 random TFs of the respective family were inserted into different genes. Values were smoothed using a 101 bp sliding window.

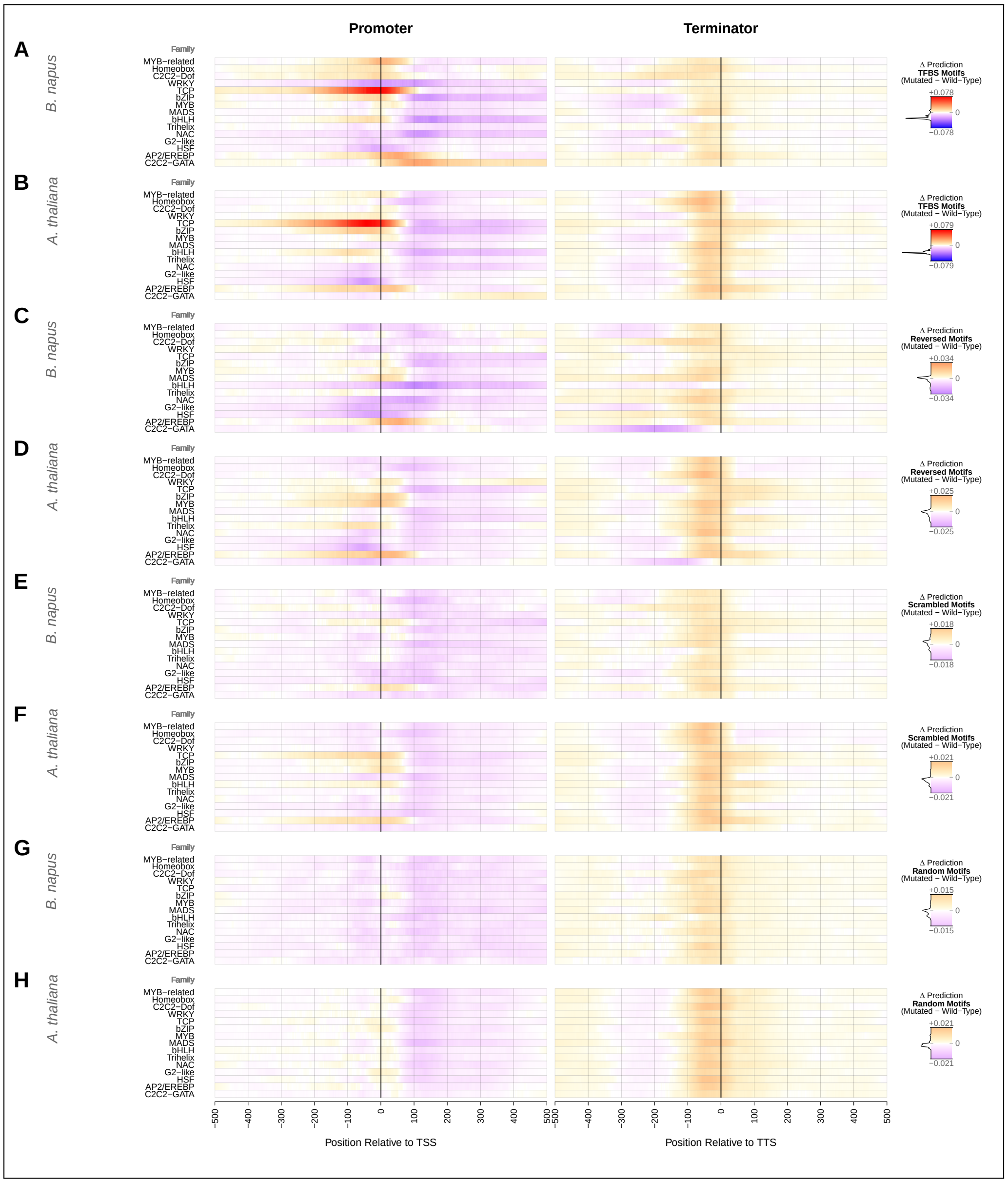

Fig. S24. Predicted change in expression based on the insertion of control motifs, with respect to the position of insertion relative to the TSS and TTS. (A-H) Color values are scaled with respect to the maximum absolute predicted change across all subplots. Red signifies a positive predicted change in expression with respect to baseline (wild-type) and blue signifies a negative predicted change in expression. The color scales indicate the range of values (± maximum absolute value), as well as the overall distribution of values within the respective subplot. (A,B) Intact TF binding motifs. (C,D) Reversed (but not complemented) versions of motifs of the respective family were inserted. (E,F) Scrambled versions of motifs of the respective family were inserted. (G,H) Random sequences with the same length as motifs from the respective family were inserted. The respective motifs used as reference motifs in each case were the same that were used for the insertion analysis in the main text. Values were smoothed using a 101 bp sliding window.

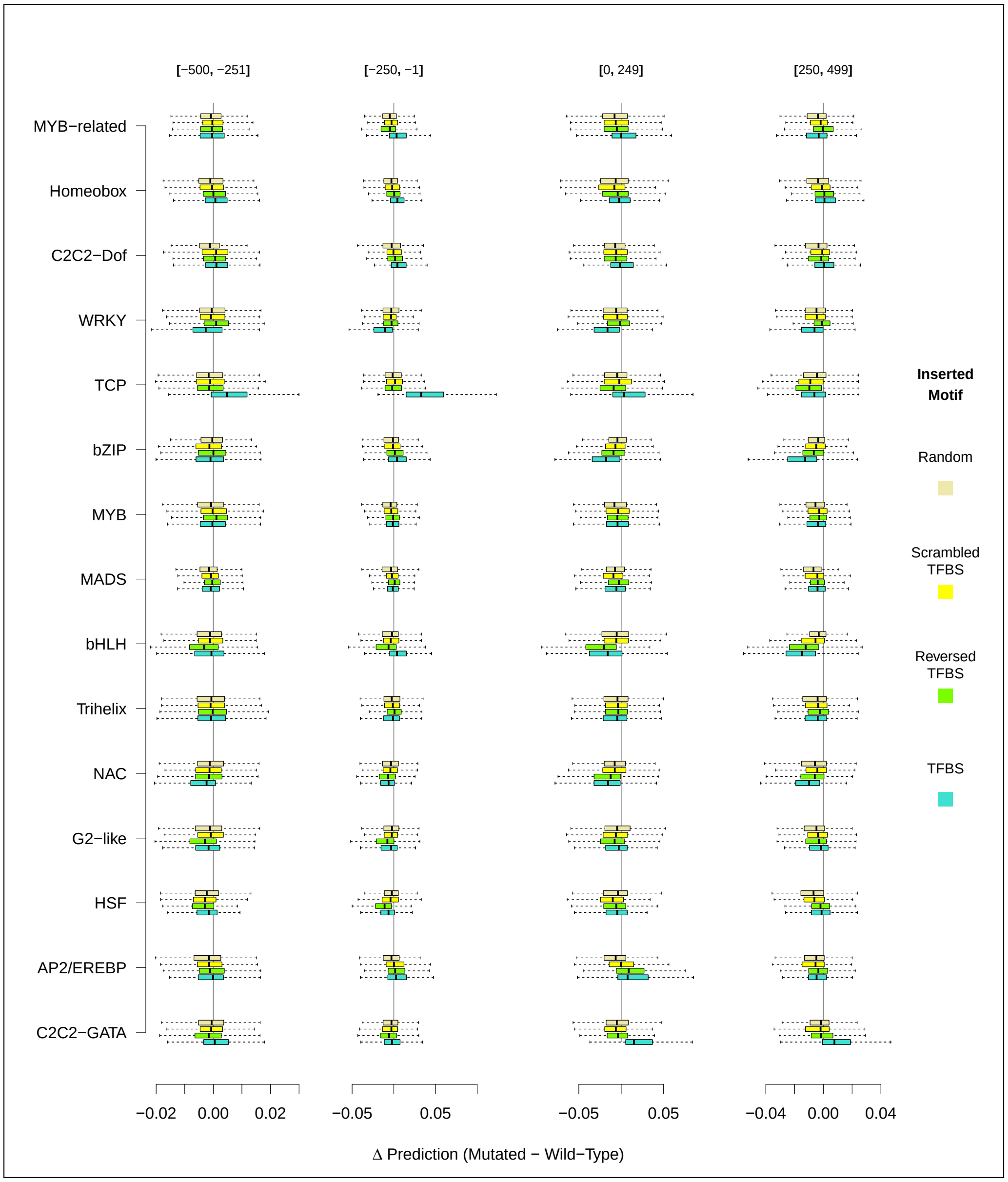

Fig. S25. Pooled effects of motif insertions into *B. napus* promoters. Boxplots show distribution of median effects across all positions in 250 bp intervals around the TSS. The boundaries of the respective pooling intervals, relative to the TSS, are indicated at the top. Colors indicate the type of motif that was inserted. Positive values (to the right of the vertical line) indicate increased predicted expression after motif insertion.

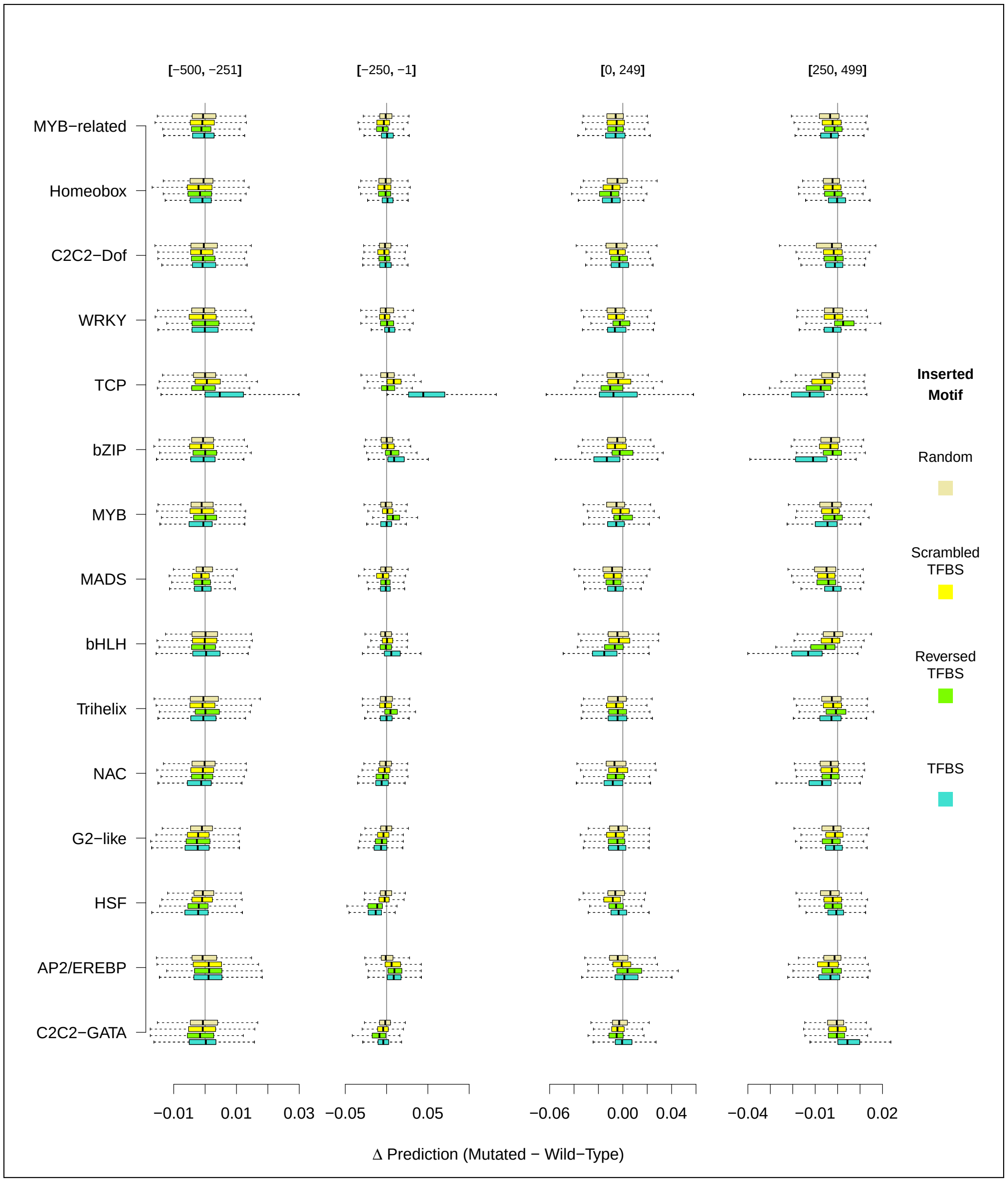

Fig. S26. Pooled effects of motif insertions into *A. thaliana* promoters. Boxplots show distribution of median effects across all positions in 250 bp intervals around the TSS. The boundaries of the respective pooling intervals, relative to the TSS, are indicated at the top. Colors indicate the type of motif that was inserted. Positive values (to the right of the vertical line) indicate increased predicted expression after motif insertion.

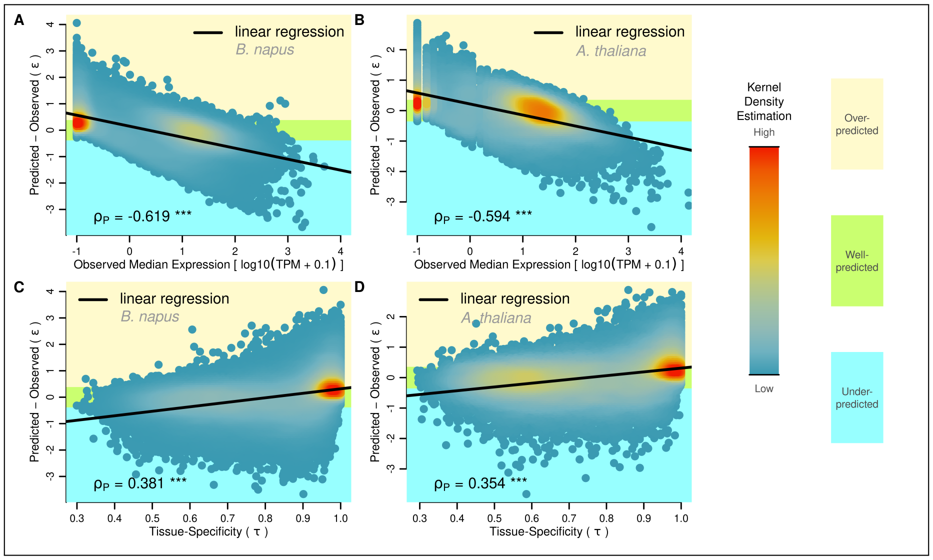

Fig. S27. Correlation of expression level and tissue-specificity on *A. thaliana* predictions. (A, B) Prediction errors are plotted against the median expression, showing Pearson correlations. (C, D) Prediction errors are plotted against the tissue-specificity calculated using the τ metric, showing Pearson correlations. Predictions were made for the entire data set via cross validation. The coloration of points is based on Gaussian kernel density estimations.

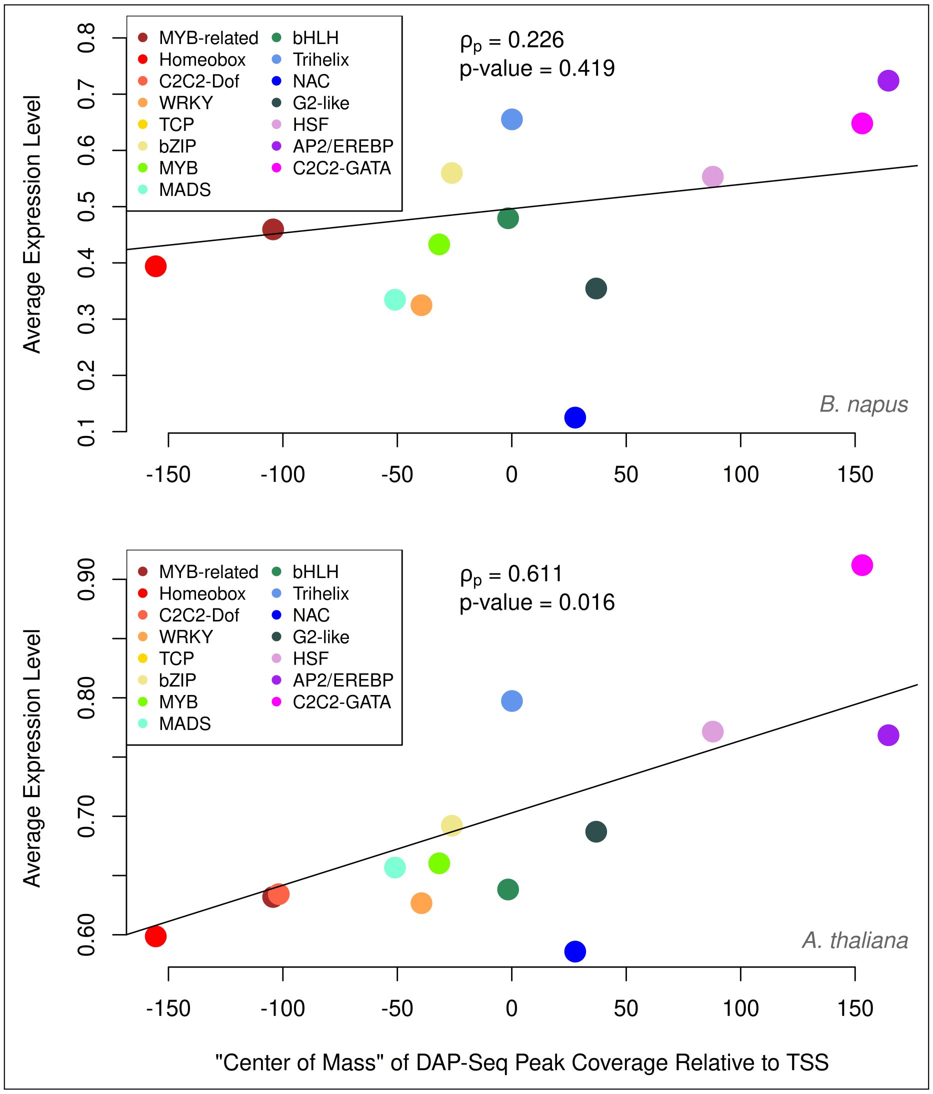

Fig. S28. Correlation of average expression levels and the "center of mass" of DAP-Seq signals. For each group of genes associated with a particular TF family, the group-specific average expression was calculated. For the corresponding DAP-Seq signals, "center of mass" was calculated by calculating a weighted average over the sequence positions $\pm$ 500 bp relative to the TSS, using the DAP-Seq peak coverage as the weight for each position.

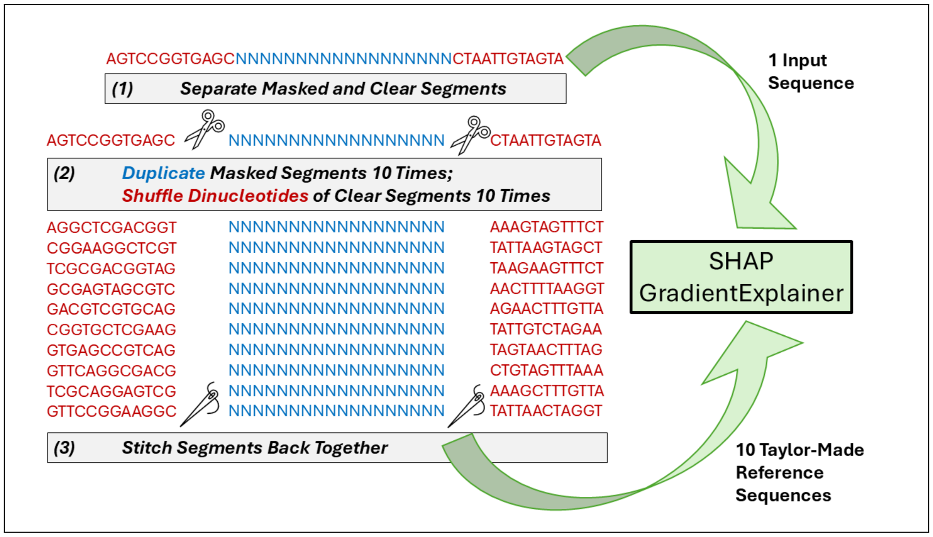

Fig. S29. Calculation of attribution scores. For each input sequence, a set of 10 reference sequences was randomly generated by shuffling dinucleotides of non-coding (clear) sequences. Coding (masked) sequences were maintained in the references, making them unimportant by definition. The input sequences and the corresponding sets of custom reference sequences were used to calculate attributions using the SHAP GradientExplainer.

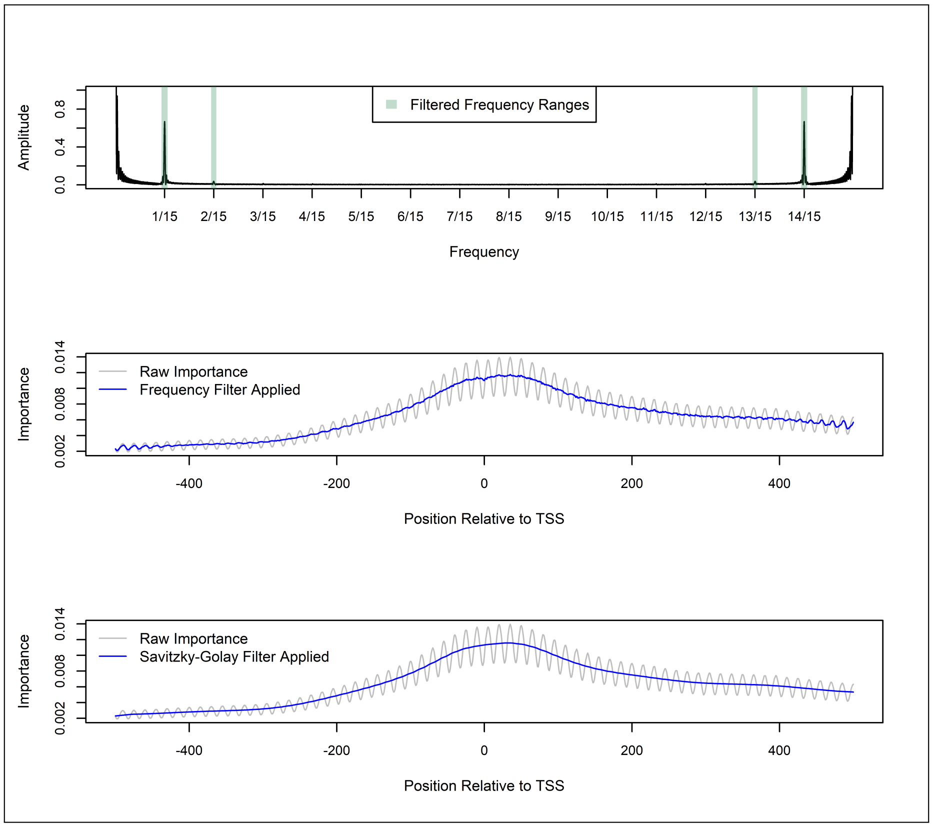

Fig. S30. Fourier analysis of importance scores. (Top) Fourier transformation of importance scores calculated across all genes. The periodic signal with a frequency of $\frac{1}{15}$ (plus harmonics) is caused by overlapping pooling windows with a stride of 15. Shaded areas show frequency bands filtered out by setting them to 0 (Middle), resulting in much smoother importance scores. Using a Savitzky-Golay filter with a polynomial order of 1 and a window size of 75, achieves similar results, but easier and cleaner (Bottom).
