# Supplementary material for: A deep learning model captures position-specific effects of plant regulatory sequences and suggests genes under complex regulation
Source: Plant Physiol. 2026 Jul 7;201(4):kiag473. doi: 10.1093/plphys/kiag473 (PMC13436691; doi:10.1093/plphys/kiag473)
Supplement: Supplementary file 1 [file kiag473_supplementary_data.zip › nemo_REVISION2_Supplementary_Tables.pdf]

Table S1. Express617 RNA-seq sample information for all tissues, stages, and studies

Tissue	Sample Name	Biological Replicate	Timepoint	PRJ	SRA
Leaf	ExL7	1	7 DAP	PRJEB107092	
Apex	ExA21a	1	21 DAP	PRJEB107092	
Apex	ExA21b	2	21 DAP	PRJEB107092	
Apex	ExA21c	3	21 DAP	PRJEB107092	
Apex	ExA42a	1	42 DAP	PRJEB107092	
Apex	ExA42b	2	42 DAP	PRJEB107092	
Apex	ExA42c	3	42 DAP	PRJEB107092	
Apex	ExA63a	1	63 DAP	PRJEB107092	
Apex	ExA63b	2	63 DAP	PRJEB107092	
Apex	ExA63c	3	63 DAP	PRJEB107092	
Apex	ExA64a	1	64 DAP	PRJEB107092	
Apex	ExA64b	2	64 DAP	PRJEB107092	
Apex	ExA64c	3	64 DAP	PRJEB107092	
Apex	ExA71a	1	71 DAP	PRJEB107092	
Apex	ExA71b	2	71 DAP	PRJEB107092	
Apex	ExA71c	3	71 DAP	PRJEB107092	
Apex	ExA98a	1	98 DAP	PRJEB107092	
Apex	ExA98b	2	98 DAP	PRJEB107092	
Apex	ExA98c	3	98 DAP	PRJEB107092	
Leaf	ExL21	1	21 DAP	PRJNA648031	SRS7070880
Leaf	ExL42	1	42 DAP	PRJNA648031	SRS7070881
Leaf	ExL63	1	63 DAP	PRJNA648031	SRS7070845
Leaf	ExL64	1	64 DAP	PRJEB107092	
Leaf	ExL71	1	71 DAP	PRJEB107092	
Leaf	ExL98	1	98 DAP	PRJEB107092	
Anther	ExAnt1_1	1	Sanders stage 12-13	PRJEB107092	
Anther	ExAnt1_2	2	Sanders stage 12-13	PRJEB107092	
Anther	ExAnt1_3	3	Sanders stage 12-13	PRJEB107092	
Bud	ExBud1_1	1	Sanders stage 6-7	PRJEB107092	
Bud	ExBud1_2	2	Sanders stage 6-7	PRJEB107092	
Bud	ExBud1_3	3	Sanders stage 6-7	PRJEB107092	
Bud	ExBud2_1	1	Sanders stage 10-11	PRJEB107092	
Bud	ExBud2_2	2	Sanders stage 10-11	PRJEB107092	
Bud	ExBud2_3	3	Sanders stage 10-11	PRJEB107092	
Gynoecia	ExGyn1_1	1	Sanders stage 12-13	PRJEB107092	
Gynoecia	ExGyn1_2	2	Sanders stage 12-13	PRJEB107092	
Gynoecia	ExGyn1_3	3	Sanders stage 12-13	PRJEB107092	
Embryo	ExpG3_1	1	Green stage	PRJEB86494	ERS23779109
Embryo	ExpG3_2	2	Green stage	PRJEB86494	ERS23779110
Embryo	ExpG3_3	3	Green stage	PRJEB86494	ERS23779111
Endosperm	ExpE3_1	1	Green stage	PRJEB86494	ERS23779112
Endosperm	Exp_E3_2	2	Green stage	PRJEB86494	ERS23779113
Endosperm	Exp_E3_3	3	Green stage	PRJEB86494	ERS23779114
Seed coat	ExpSC3_1	1	Green stage	PRJEB86494	ERS23779115
Seed coat	Exp_SC3_2	2	Green stage	PRJEB86494	ERS23779116
Seed coat	Exp_SC3_3	3	Green stage	PRJEB86494	ERS23779117
Silique wall	ExpSW3_1	1	Green stage	PRJEB86494	ERS23779118
Silique wall	ExpSW3_2	2	Green stage	PRJEB86494	ERS23779119
Silique wall	ExpSW3_3	3	Green stage	PRJEB86494	ERS23779120
Gynoecia wall	ExpGW_1	1	Pre-fertilisation (24h before anthesis)	PRJEB86494	ERS23779136
Gynoecia wall	ExpGW_2	2	Pre-fertilisation (24h before anthesis)	PRJEB86494	ERS23779137
Gynoecia wall	ExpGW_3	3	Pre-fertilisation (24h before anthesis)	PRJEB86494	ERS23779138

Embryo	ExpH1_1	1	Heart stage	PRJEB86494	ERS23779121
Embryo	Exp_H1_2	2	Heart stage	PRJEB86494	ERS23779122
Embryo	Exp_H1_4	3	Heart stage	PRJEB86494	ERS23779123
Endosperm	ExpE1_1	1	Heart stage	PRJEB86494	ERS23779124
Endosperm	ExpE1_2	2	Heart stage	PRJEB86494	ERS23779125
Endosperm	Exp_E1_4	3	Heart stage	PRJEB86494	ERS23779126
Seed coat	ExpSC1_1	1	Heart stage	PRJEB86494	ERS23779127
Seed coat	Exp_SC1_2	2	Heart stage	PRJEB86494	ERS23779128
Seed coat	Exp_SC1_4	3	Heart stage	PRJEB86494	ERS23779129
Embryo	ExpM4_1	1	Mature (not fully dry)	PRJEB86494	ERS23779130
Embryo	ExpM4_2	2	Mature (not fully dry)	PRJEB86494	ERS23779131
Embryo	ExpM4_3	3	Mature (not fully dry)	PRJEB86494	ERS23779132
Seed coat	Exp_SCM_1	1	Mature (not fully dry)	PRJEB86494	ERS23779133
Seed coat	Exp_SCM_2	2	Mature (not fully dry)	PRJEB86494	ERS23779134
Seed coat	Exp_SCM_3	3	Mature (not fully dry)	PRJEB86494	ERS23779135
Ovules	ExpOV_1	1	Pre-fertilisation (24h before anthesis)	PRJEB86494	ERS23779139
Ovules	ExpOV_2	2	Pre-fertilisation (24h before anthesis)	PRJEB86494	ERS23779140
Ovules	ExpOV_3	3	Pre-fertilisation (24h before anthesis)	PRJEB86494	ERS23779141
Embryo	ExpT2_1	1	Torpedo stage	PRJEB86494	ERS23779142
Embryo	Exp_T2_2	2	Torpedo stage	PRJEB86494	ERS23779143
Embryo	Exp_T2_4	3	Torpedo stage	PRJEB86494	ERS23779144
Endosperm	ExpE2_1	1	Torpedo stage	PRJEB86494	ERS23779147
Endosperm	ExpE2_2	2	Torpedo stage	PRJEB86494	ERS23779146
Endosperm	Exp_E2_4	3	Torpedo stage	PRJEB86494	ERS23779145
Seed coat	ExpSC2_1	1	Torpedo stage	PRJEB86494	ERS23779148
Seed coat	Exp_SC2_2	2	Torpedo stage	PRJEB86494	ERS23779149
Seed coat	Exp_SC2_4	3	Torpedo stage	PRJEB86494	ERS23779150
Root	RNA_Rt1	1	9 DAP	PRJNA1359611	
Root	RNA_Rt2	2	9 DAP	PRJNA1359611	
Seedling	RNA_Sd1	1	4 DAP	PRJNA1359611	
Seedling	RNA_Sd2	2	4 DAP	PRJNA1359611	
Leaf	RNA_Lf1	1	28 DAP	PRJNA1359611	
Leaf	RNA_Lf2	2	28 DAP	PRJNA1359611	
Silique	RNA_Sq1	1	based on size	PRJNA1359611	
Silique	RNA_Sq2	2	based on size	PRJNA1359611	
Flower	RNA_Fl1	1	based on size	PRJNA1359611	
Flower	RNA_Fl2	2	based on size	PRJNA1359611	

The hyperparameters were tuned in six stages, namely, optimizing the architecture, validating the architecture, fine-tuning the architecture, optimizing the training strategy, fine-tuning the training strategy and some final toches. Search spaces are given as a list of items "(item1, item2)" or a numeric range "[from, to]". An asterisk (*) symbol after the closing bracket signifies that the values were sampled from a logarithmic distribution. Hyperparameters that apply for multiple layers were individually optimized for each layer. The best values found are then given as a descending list from shallow layers to deeper layers. P and T signify values that apply to the promoter and terminator branches, respectively. In the first three stages, the hyperparameters were tuned to minimize the validation loss. For all stages the same 80% of data were used as a training set, 10% were used for validation and 10% were held out. In the first stage (Table S2), the Optuna TPE sampler was used to sample hyperparameters hierarchically, meaning that the higher order hyperparameters depend on one or more lower order hyperparameters. The respective conditional relationships are given in gray italics. The conditions correspond to a

Boolean hyperparameter above, unless stated otherwise. In the second stage (Table S3) the Optuna grid sampler was used to test the optimized model architecture against simplified versions of the model with shared hyperparameters between layers and/or branches. In the third stage (Table S4) the architecture was fine-tuned using the TPE sampler with a narrow search space around the best values found in the first two stages and small step sizes. Before the fourth stage, a learning rate range test was performed to find the highest possible learning rate, using a modified version of a publicly available range finder (https://github.com/surmenok/keras_lr_finder). This was done to implement a one-cycle learning rate policy. For the learning rate range test and the subsequent hyperparameter optimizations, the batch size was fixed at a value of 130, which is the highest batch size found that did not lead to crashes, due to exceeding the memory capacity. In stages four five and six, hyperparameters relating to the training itself were optimized, including the loss function, therefore, instead of minimizing validation loss, hyperparameters were tuned to maximize the coefficient of determination on the validation set. While in stage four, the respective hyperparameters were tuned across a broad search space, they were tuned across a narrow space around the previously best found vales in stage five, using small step sizes, in order to arrive at the final model. In stage six, some final touches were made to three hyperparameters that potentially influence training efficiency.

Table S2. Hyperparameter search space for optimizing the neural network architecture
Optuna TPE sampler used, validation MSE minimized, 100 warm-up trials
Total number of trials: 1122
Number of pruned trials: 892
Number of complete trials: 209
Number of failed trials: 20

1st order	2nd order	3rd order	4th order	search space	step	best value
outside sequence interval(1)	-	-	-	$[1000, 15000]$	1000	5000
inside sequence interval(2)	-	-	-	$[100, 1500]$	100	1200
batch size	-	-	-	$2^{[2,7]}$	1	2^5
number of convolutional layers	-	-	-	$[1, 6]$	1	P:6; T:2
number of LSTM layers	-	-	-	$[0, 2]$	1	P:0; T:2
use exponential activation(3)	-	-	-	$(True, False)$	boolean	*False*
if True	first_layer_kernel_size	-	-	$2^{[4,5]}$	1	-
convolutional kernel size	-	-	-	$2^{[2,5]}$	1	P:$2^{(2,2,4,2,5,5)}$; T:$2^{(2,3)}$
number of convolutional filters	-	-	-	$2^{[5,9]}$	1	P:$2^{(9,8,7,7,9,6)}$; T:$2^{(5,5)}$
pool size	-	-	-	$2^{[0,6]}$	1	P:$2^{(2,2,4,2,5,5)}$; T:$2^{(2,3)}$
pool type(4)	-	-	-	(max, avrg)	categorical	P:(m,a,a,m,a,m); T:(a,m)
fraction of pool size used as pool stride(5)	-	-	-	[0.5, 1.0]	0.1	P:(0.7,0.5,1.0,0.9,0.5,0.7); T:(0.7,1.0)
fraction of kernel size used as conv. stride(5)	-	-	-	[0.0, 0.5]	0.1	P:(0.0,0.2,0.2,0.5,0.5,0.3); T:(0.5,0.0)
activation function	-	-	-	(ReLU, LReLU, PReLU, SELU, GELU, ELU, Swish, SoftPlus, Mish)	categorical	GELU
if LReLU or PReLU	alpha parameter of ReLU function(6)	-	-	$[5 \cdot 10^{-6}, 5 \cdot 10^{-1}]$*	continuous	-
if ELU	alpha parameter of ELU function(7)	-	-	[0.2, 2]*	continuous	-
if not (ReLU or LReLU or PReLU or SELU)	initialization(8)	-	-	(He_normal, Glorot_normal)	categorical	Glorot_normal
1 - μ (Batchnorm momentum)(9)				$[1 \cdot 10^{-4}, 4 \cdot 10^{-1}]$*	continuous	0.0823
use dropout(10)	-	-	-	$(True, False)$	boolean	*True*
if True	use dropout only after last layer	-	-	$(True, False)$	boolean	*True*
-	*if True*	last layer dropout rate	-	$[10^{-5}, 1]$*	continuous	0.0677
-	*if False*	dense layer dropout rate	-	$[10^{-5}, 1]$*	continuous	-
-	*if False*	use dropout after conv. layers	-	$(True, False)$	boolean	-
-	-	*if True*	conv. layer dropout rate	$[10^{-5}, 1]$*	continuous	-
-	*if False & nlstm > 0*	use dropout after LSTM layer	-	$[True, False]$	boolean	-
-	-	*if True*	LSTM layer dropout	$[10^{-5}, 1]$*	continuous	-
if nlstm > 0	type of skip connection	-	-	(none, add, mult, concat)(11)	categorical	none
if number of LSTM layers > 0	LSTM units(12)	-	-	$2^{[5,9]}$	1	T: $2^{(5,5)}$
number of dense layers	-	-	-	$[1, 3]$	1	1
number of dense layer neurons	-	-	-	$2^{[1,10]}$	1	2^3

(1) upstream of TSS and downstream of TTS; (2) downstream of TSS and upstream of TTS ;(3) after the first convolutional layer; (4) "max"/"m" = max-pooling, "avrg"/"a" = average pooling; (5) $stride = max(1, \lceil size \cdot fraction \rceil)$; (6) Slope for LReLU and initialization of the slope for PReLU; (7) smoothness of ELU function; (8) For ReLU-like activations, initialization was set to He_normal, for SELU it was set to LeCun_normal; (9) μ = Batchnormalization momenturm; (10) In case of SELU activation, alpha-dropout was used instead of regular dropout; (11) Implementation of skip-connection across LSTM layers. "none" = no skip connection; "add" = element-wise addition; "mult" = element-wise multiplication; "concat" = concatenation along feature dimension; (12) In case of an additive or multiplicative skip connection, the LSTM units of the last LSTM layer were set to the same dimension as the feature axis of the skip-connection, regardless of the hyperparameter value.

Table S3. **Hyperparameter search space for validating the architecture**
Optuna grid sampler used, validation MSE minimized
Total number of trials: 49
Number of pruned trials: 0
Number of complete trials: 47
Number of failed trials: 2

hyperparameter	search space	best value
promoter branch$^{(1)}$	[promoter hyperparameters, terminator hyperparameters]	promoter hyperparameters
terminator branch$^{(1)}$	[promoter hyperparameters, terminator hyperparameters]	promoter hyperparameters
use default conv. stride$^{(2)}$	$[True, False]$	$True$
use default pool stride$^{(3)}$	$[True, False]$	$False$
(global) pool type$^{(4)}$	[max, average, optimized]	average

(1) Optimal hyperparameters for the promoter and terminator branch were validated by making them interchangeable and testing all possible combinations; (2) the default convolutional stride is 1; (3) the default stride of pooling layers is equal to the respective pooling window size; (4) global pool types (same type used for all pooling layers) were tested against the optimized set of pooling types that were optimized for each individual pooling layer.

Table S4. **Hyperparameter search space for fine-tuning the neural network architecture**
Optuna TPE sampler used, validation MSE minimized, 100 warm-up trials
Total number of trials: 1110
Number of pruned trials: 966
Number of complete trials: 135
Number of failed trials: 8

1st order	2nd order	search space	step	best value
batch size	-	$[16, 32]$*	1	18
kernel size (conv. layers 1 and 2)	-	$[2, 8]$*	1	P:$(5, 8)$; T:$(4, 4)$
kernel size (conv. layers 3 and 4)	-	$[4, 16]$*	1	P:$(9, 10)$; T:$(16, 15)$
kernel size (conv. layers 5 and 6)	-	$[16, 64]$*	1	P:$(46, 62)$; T:$(20, 22)$
number of conv. filters$^{(1)}$	-	$[64, 512]$*	1	P:$(288, 106, 227, 187, 64, 152)$;
				T:$(219, 449, 259, 154, 105, 152)$
pool size$^{(2)}$	-	$[1, 64]$*	1	$(1, 21, 1, 1, 18, 8)$
fraction of pool size used as stride$^{(3)}$	-	[0.5, 1.0]	0.1	(0.6, 0.7, 0.8, 0.6, 0.7, 0.7)
use cross-branch LSTM layer$^{(4)}$	-	[True, False]	boolean	False
if True	number of LSTM units	[16, 128]*	1	-
number of dense layers	-	$[1, 2]$	1	2
number of neurons (dense layer 1)	-	$[8, 1024]$*	1	750
# dense layers > 1	number of neurons (dense layer 2)	$[2, 8]$*	1	3
dropout rate after last layer	-	$[0.005, 0.5]$*	continuous	0.01063
$1 - \mu^{(5)}$	-	$[0.01, 0.2]$*	continuous	0.15784

(1) The number of filters for the last convolutional layer was only sampled once and used for both branches, in order to ensure that the output dimensions are identical between the two branches; (2) pool sizes were only sampled once and used for both branches, in order to ensure that the output dimensions are identical between the two branches; (3) $pool\_stride = max(1, \lceil size \cdot fraction \rceil)$; (4) LSTM layer applied to the concatenated outputs of both convolutional branches; (5) µ = Batchnormalization momenturm.

Table S5. Hyperparameter search space for optimizing model training
Optuna TPE sampler used, validation r^2 maximized, 100 warm-up trials
Total number of trials: 623
Number of pruned trials: 0
Number of complete trials: 608
Number of failed trials: 13

hyperparameter	search space	step	best value
number of training epochs	[3, 30]*	1	6
$1 - \beta_2{}^{(1)}$	[10^{-5},10^{-1}]*	continuous	0.001793
$\varepsilon^{(2)}$	[10^{-8},10^{-6}]*	continuous	$8.327 \cdot 10^{-7}$
weight decay	[10^{-6}, 10^{-4}]*	continuous	$3.209 \cdot 10^{-5}$
learning rate peak shift$^{(3)}$	[0.2, 0.8]	continuous	0.7954
final learning rate scale$^{(4)}$	[0, 1]	continuous	0.8513
maximum learning rate$^{(5)}$	[0.01584893, 0.37850893]*	continuous	$2.965 \cdot 10^{-2}$
maximum $\beta_1{}^{(6)}$	[0.9, 0.99]	continuous	0.9288
minimum $\beta_1{}^{(6)}$	[0.8, 0.9]	continuous	0.8630
huber loss $\delta^{(7)}$	[0.135, 13.5]	continuous	11.267
dropout rate after last layer$^{(8)}$	[0.0, 0.01063]	continuous	$1.243 \cdot 10^{-3}$

(1) β_2 corresponds to the "beta_2" parameter of the Tensorflow implementation of the Nadam algorithm; (2) ε corresponds to the "epsilon" parameter of the Tensorflow implementation of the Nadam algorithm; (3) the relative position, at which the learning rate reaches its peak with respect to the complete cycle length; (4) relative scale of the final learning rate at the end of the cycle, with respect to initial learning rate, see 5; (5) the lower bound of the maximum learning rate is the learning rate at which, during the learning rate range test, the lowest training loss overall was reached, while the upper bound is the highest learning rate that was reached before the training loss began rapidly diverging; $initial\ LR = \frac{max(LR)}{25}$; (6) β_1 corresponds to the "beta_1" parameter of the Tensorflow implementation of the Nadam algorithm, which corresponds to momentum and is cycled in the opposite direction of the learning rate; (7) corresponds to the "delta" parameter of the Tensorflow implementation of Huber loss; (8) since high learning rates have a regularizing effect, dropout regularization is adjusted.

Table S6. Hyperparameter search space for fine-tuning model training
Optuna TPE sampler used, validation r^2 maximized, 10 warm-up trials
Total number of trials: 533
Number of pruned trials: 0
Number of complete trials: 511
Number of failed trials: 21

hyperparameter	search space	step	best value
number of training epochs$^{(1)}$	[6, 10]	1	6
final learning rate scale	[0, 0.9]	continuous	0.8758
maximum learning rate	[$2.965 \cdot 10^{-2}$, $5 \cdot 10^{-2}$]	continuous	$4.873 \cdot 10^{-2}$
initial learning rate	[$\frac{max.\ learning\ rate}{25}$, $\frac{max.\ learning\ rate}{20}$]	continuous	$1.299 \cdot 10^{-3}$
maximum β_1	[0.9, 0.95]	continuous	0.9274
minimum β_1	[0.83, 0.89]	continuous	0.8899
huber loss $\delta^{(2)}$	[0.135, 13.5]*	continuous	2.296

(1) The learning rate peak is shifted according to the number of epochs, to maintain a constant relative location with respect to the whole cycle length; (2) sampled from logarithmic distribution in this stage.

Table S7. Hyperparameter search space for final touches to model training
Optuna TPE sampler used, validation r^2 maximized, 10 warm-up trials
Total number of trials: 322
Number of pruned trials: 0
Number of complete trials: 311
Number of failed trials: 9

hyperparameter	search space	step	best value
maximum learning rate	[0.01, 0.1]	continuous	$6.630 \cdot 10^{-2}$
Batchnormalization Momentum (μ)$^{(1)}$	[0.7, 0.9]	continuous	0.8167
huber loss δ	[1.0, 3.0]	continuous	2.9466

(1) The optimal value of μ depends on the batch size. Since it had previously been optimized at lower batch sizes, it needed to be adjusted to the high batch size used for the one-cycle learning rate policy.

Table S8. Final set of best hyperparameters

hyperparameter	best value
batch size	130
kernel initialization	Glorot normal
activation function	GELU
length of outside sequence interval	5000
length of inside sequence interval	1200
number of convolutional layers	6
number of conv. filters	P:$(288, 106, 227, 187, 64, 152)$; T:$(219, 449, 259, 154, 105, 152)$
size of conv. kernels	P:$(5, 8, 9, 10, 46, 62)$; T:$(4, 4, 16, 15, 20, 22)$
pooling type	average pooling
size of pooling windows	P&T: (1, 21, 1, 1, 18, 8)
pooling strides	P&T: (1, 15, 1, 1, 13, 6)
number of dense layers	2
number of dense layer neurons	$(750, 3)$
dropout rate after last layer	$1.243 \cdot 10^{-3}$
number of epochs	6
learning rate peak shift	0.7954
initial learning rate	$1.299 \cdot 10^{-3}$
maximum learning rate	$6.630 \cdot 10^{-2}$
final learning rate scale	0.8758
initial Nadam β_1	0.9274
minimum Nadam β_1	0.8899
Nadam β_2	0.9982
Nadam ε	$1.133 \cdot 10^{-6}$
Nadam weight decay	$3.209 \cdot 10^{-5}$
Batchnormalization momentum (μ)	0.8167
huber loss δ	2.9466
