# Supplementary material for: A deep learning model captures position-specific effects of plant regulatory sequences and suggests genes under complex regulation
Source: Plant Physiol. 2026 Jul 7;201(4):kiag473. doi: 10.1093/plphys/kiag473 (PMC13436691; doi:10.1093/plphys/kiag473)
Supplement: Supplementary file 1 [file kiag473_supplementary_data.zip › nemo_REVISION2_Supplementary_Figures_1.pdf]

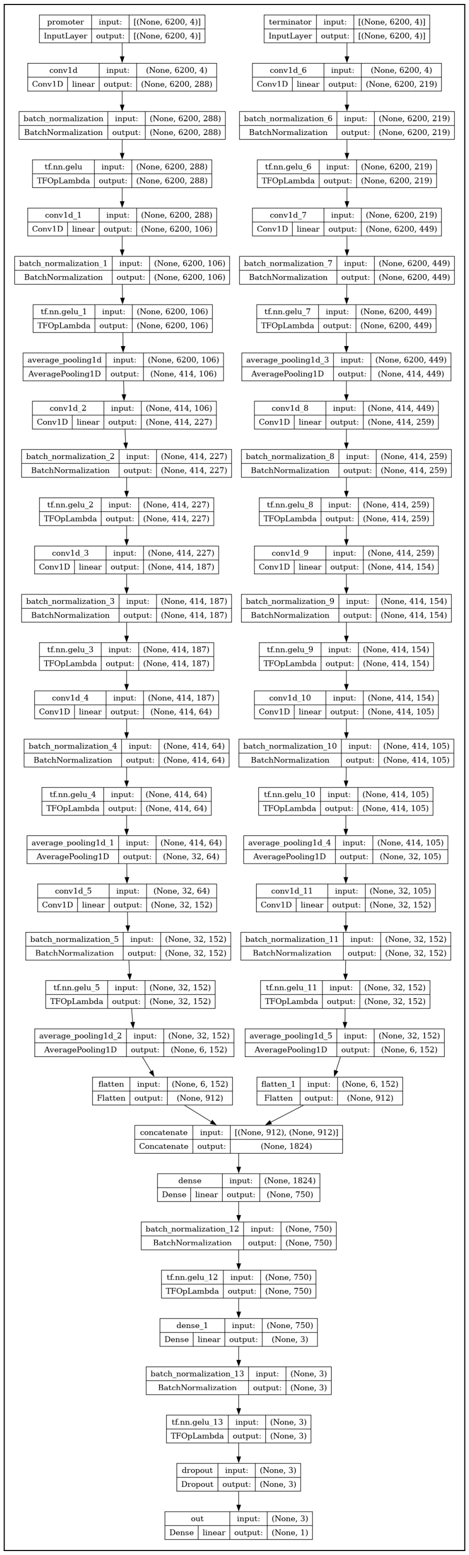

Fig. S1. The final *n*emo architecture. After hyperparameter optimization, the architecture contains a stack of six convolutional layers in each convolutional branch, interspersed by batch normalization, GELU activation and average pooling. Information processing is done in two dense layers interspersed by batch normalization and GELU activation before a final dropout layer and the output.

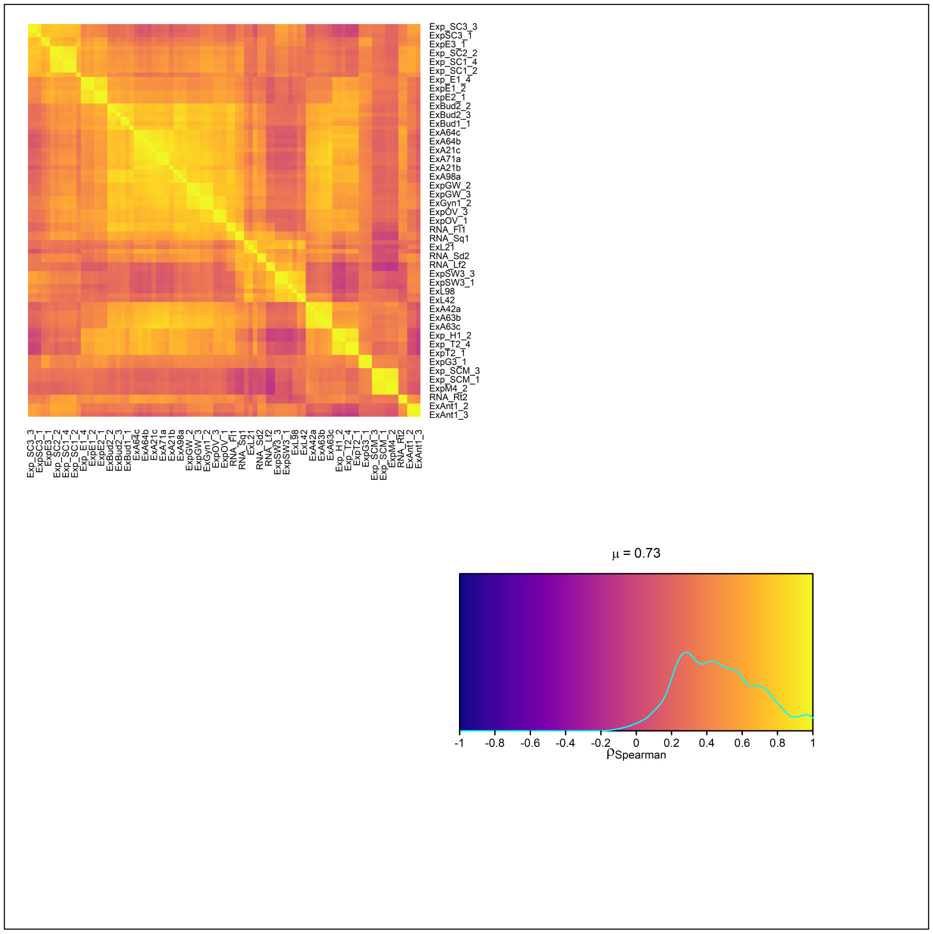

Fig. S2. Pair-wise Spearman correlations between *B. napus* samples based on TPM-normalized expression values. The 5000 most highly expressed genes across samples were used to compute correlation coefficients. The overall distribution of correlation coefficients is depicted in the bottom right.

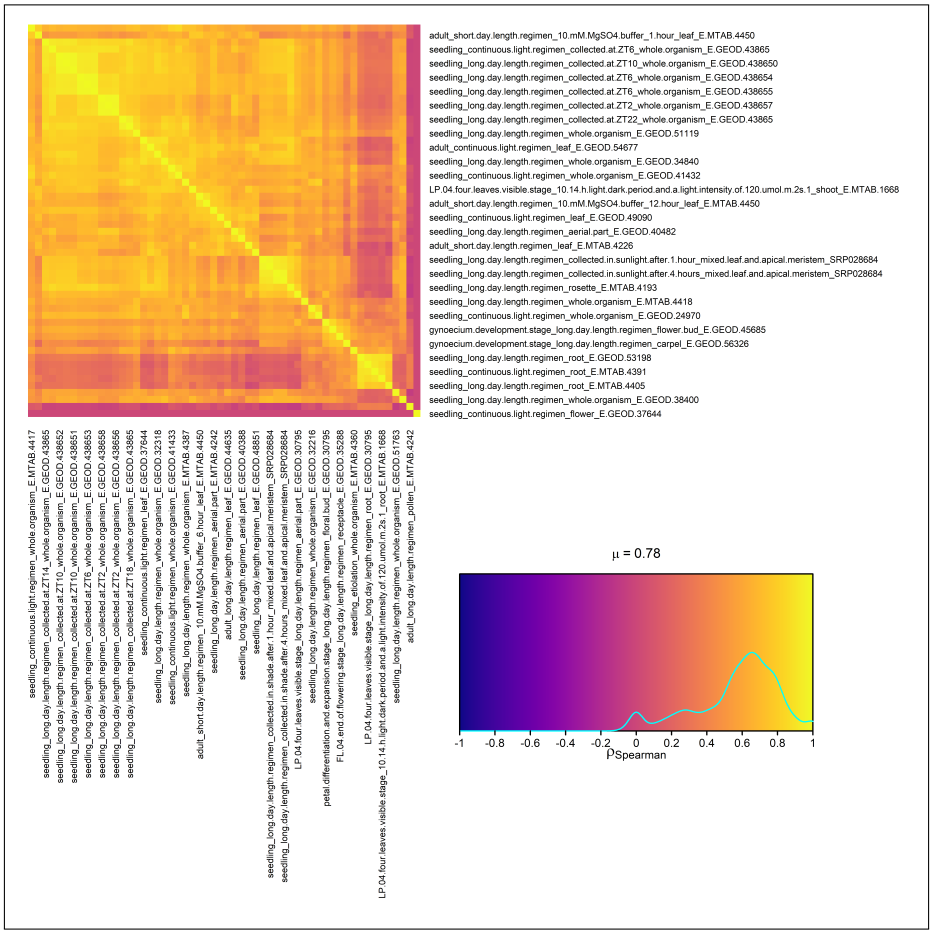

Fig. S3. Pair-wise Spearman correlations between *A. thaliana* samples based on TPM-normalized expression values. The 5000 most highly expressed genes across samples were used to compute correlation coefficients. The overall distribution of correlation coefficients is depicted in the bottom right. Due to their low overall correlation, two samples ("E.GEOD.37644", "E.MTAB.4242") were discarded.

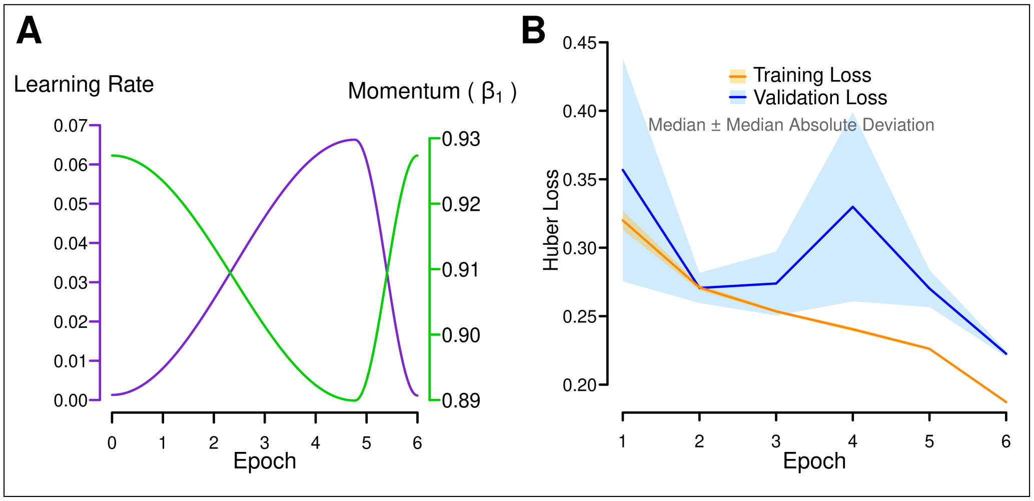

Fig. S4. Model training using one-cycle learning rate schedule. (A) The one-cycle schedules that are applied to the learning rate (left axis) and momentum (right axis). (B) Training and validation loss dynamics during training. Lines and shaded areas represent the median ± the median absolute deviation at each training epoch for 10 training runs using the same training set.

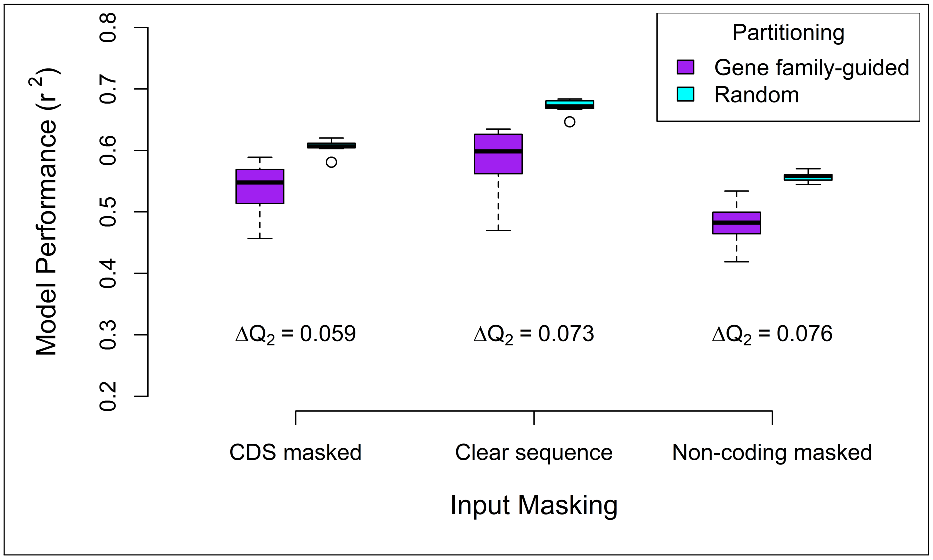

Fig. S5. The effect of CDS masking on overinflated performance estimations in *B. napus*. Differences in median performance (ΔQ_2) between gene family-guided partitioning (purple) and random partitioning (cyan) are lowest when masking coding sequences (left), versus retaining the complete sequence (middle) or masking non-coding sequences (right). For this analysis instances of *n*emo$_{90}$ were trained on 90% of data from differentially masked/partitioned data sets and tested on the remaining 10% of the same set. This was done for all data fold permutations of each data set.

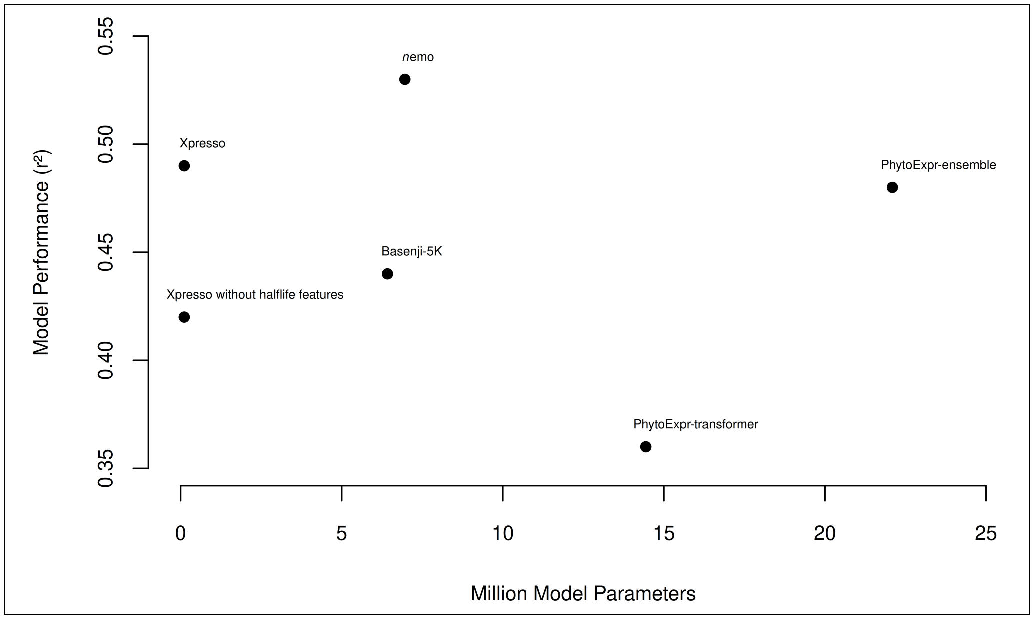

Fig. S6. Median model performance across 10 training runs on 80% of data, tested on 10% of data containing gene families not seen during training. Performance is plotted against model sizes in terms of total parameter count.

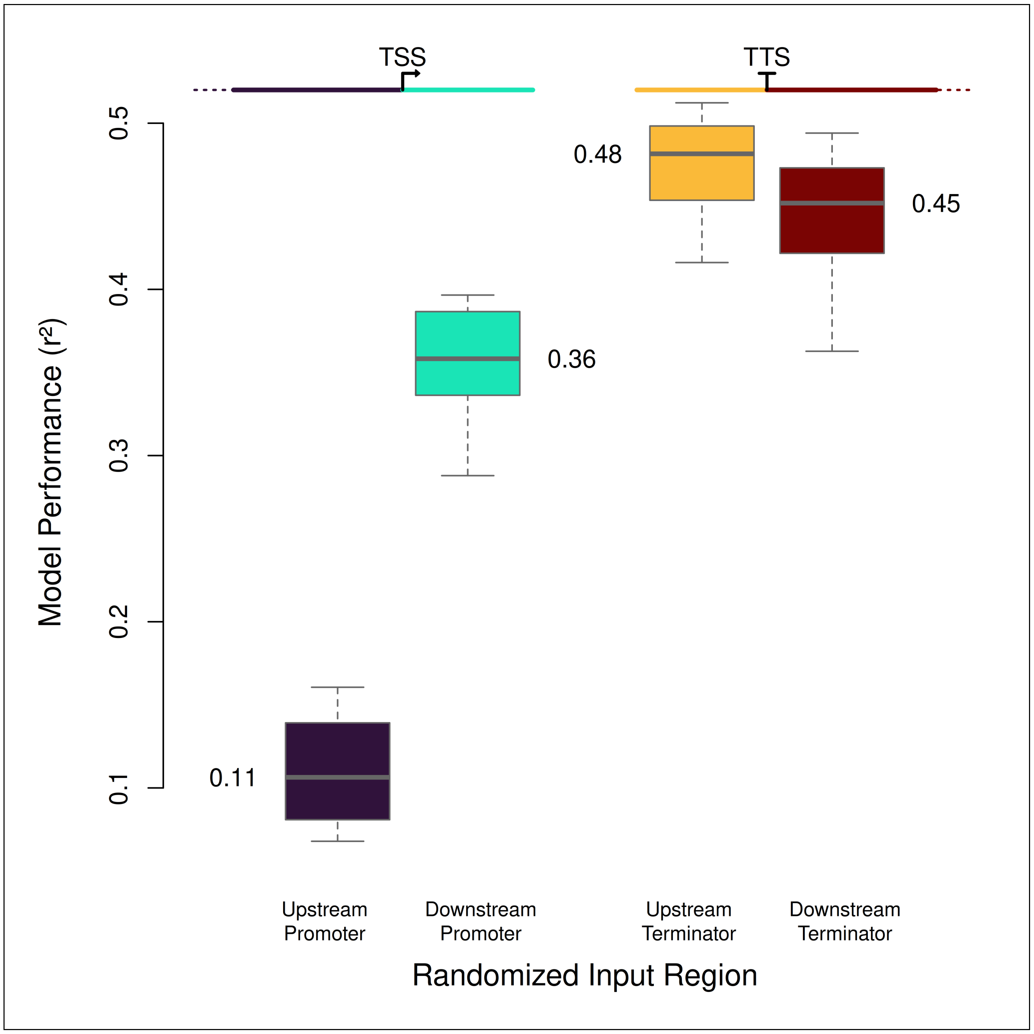

Fig. S7. Performance of *n*emo$_{90}$ instances on partially randomized versions of all ten data folds (each box is made from 10 data points, 1 per data fold). Each box represents prediction performance after randomization of the respective region in the input sequence, while maintaining masked segments from the original sequence.

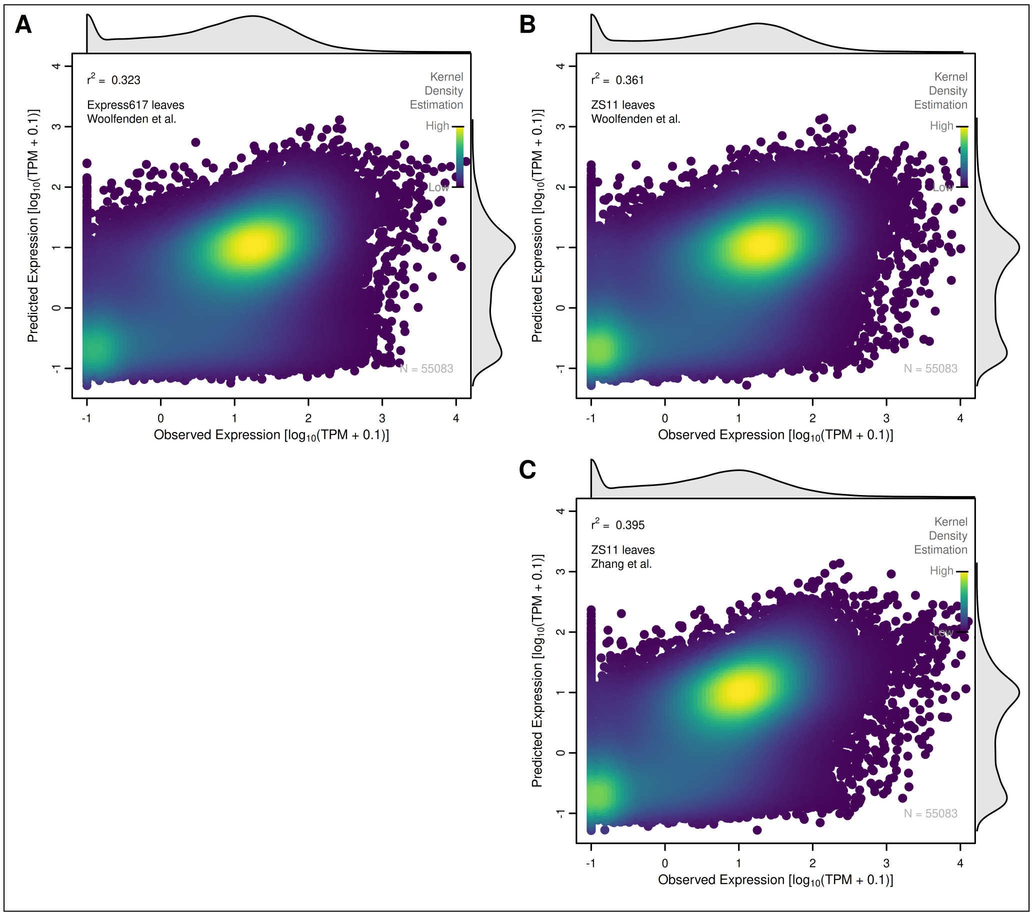

Fig. S8. Predictions of *n*emo$_{100}$ on genes from the two *B. napus* ecotypes Express617 and ZS11, plotted against observed median expression across leaf samples. (A) Express617 leaf data from Woolfenden et al. together with an additional leaf sample (21 DAP) produced for this study by the same lab. (B,C) ZS11 leaf data from two different labs: Woolfenden et al. (B) and Zhang et al. (C).

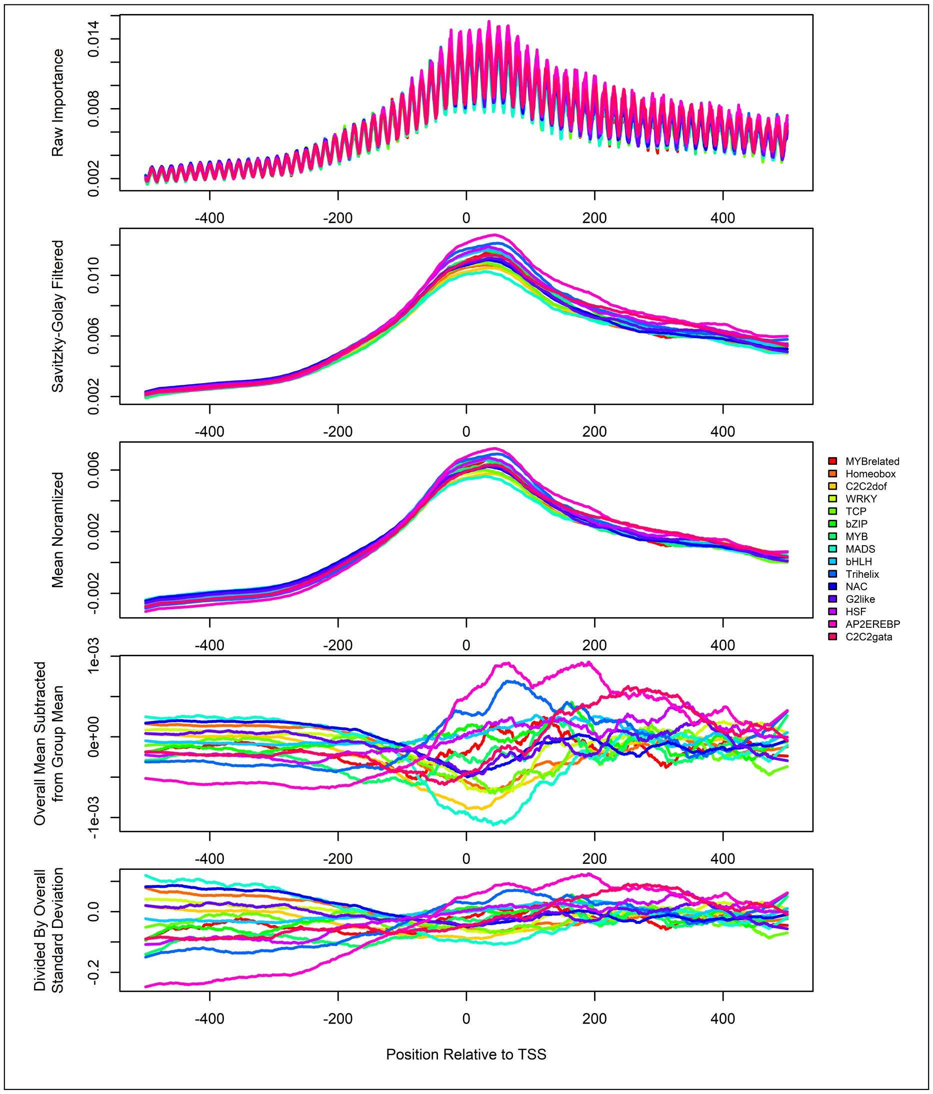

Fig. S9. Normalization of group-specific importance scores (*B. napus*). Savitzky-Golay filters are applied to filter out the periodic noise signal caused by overlapping pooling windows. Filtered importance scores are first mean normalized by subtracting the average importance across the sequence from the scores at each position. This mitigates bias due to differences in average expression between groups. Then, at each position, the (mean-normalized) average importance across all genes is subtracted from the (mean-normalized) group-specific importance in order to arrive at group-specific residual scores. Lastly, at each position, the residual score is divided by the standard deviation across all genes, in order to emphasize differences in less-variable regions and de-emphasize differences in highly variable regions.

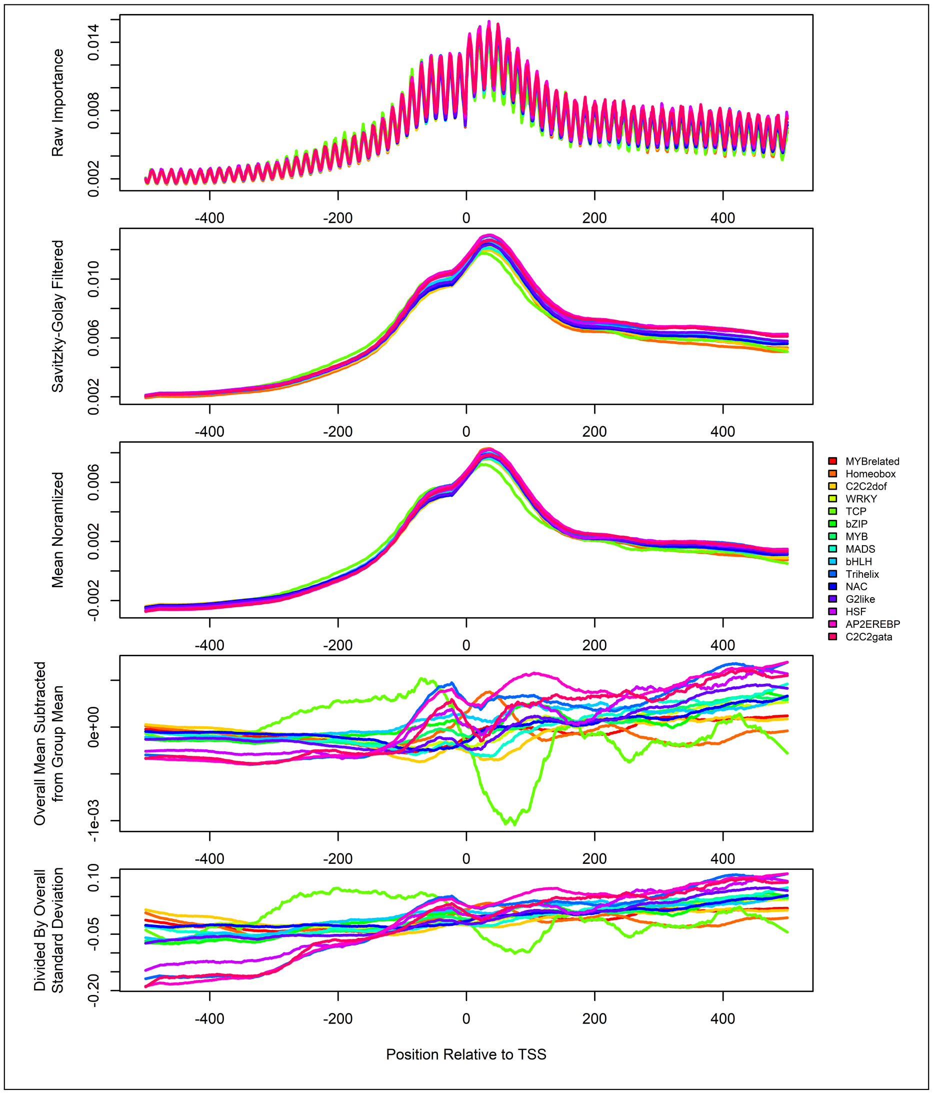

Fig. S10. Normalization of group-specific importance scores (*A. thaliana*). Savitzky-Golay filters are applied to filter out the periodic noise signal caused by overlapping pooling windows. Filtered importance scores are first mean normalized by subtracting the average importance across the sequence from the scores at each position. This mitigates bias due to differences in average expression between groups. Then, at each position, the (mean-normalized) average importance across all genes is subtracted from the (mean-normalized) group-specific importance in order to arrive at group-specific residual scores. Lastly, at each position, the residual score is divided by the standard deviation across all genes, in order to emphasize differences in less-variable regions and de-emphasize differences in highly variable regions.

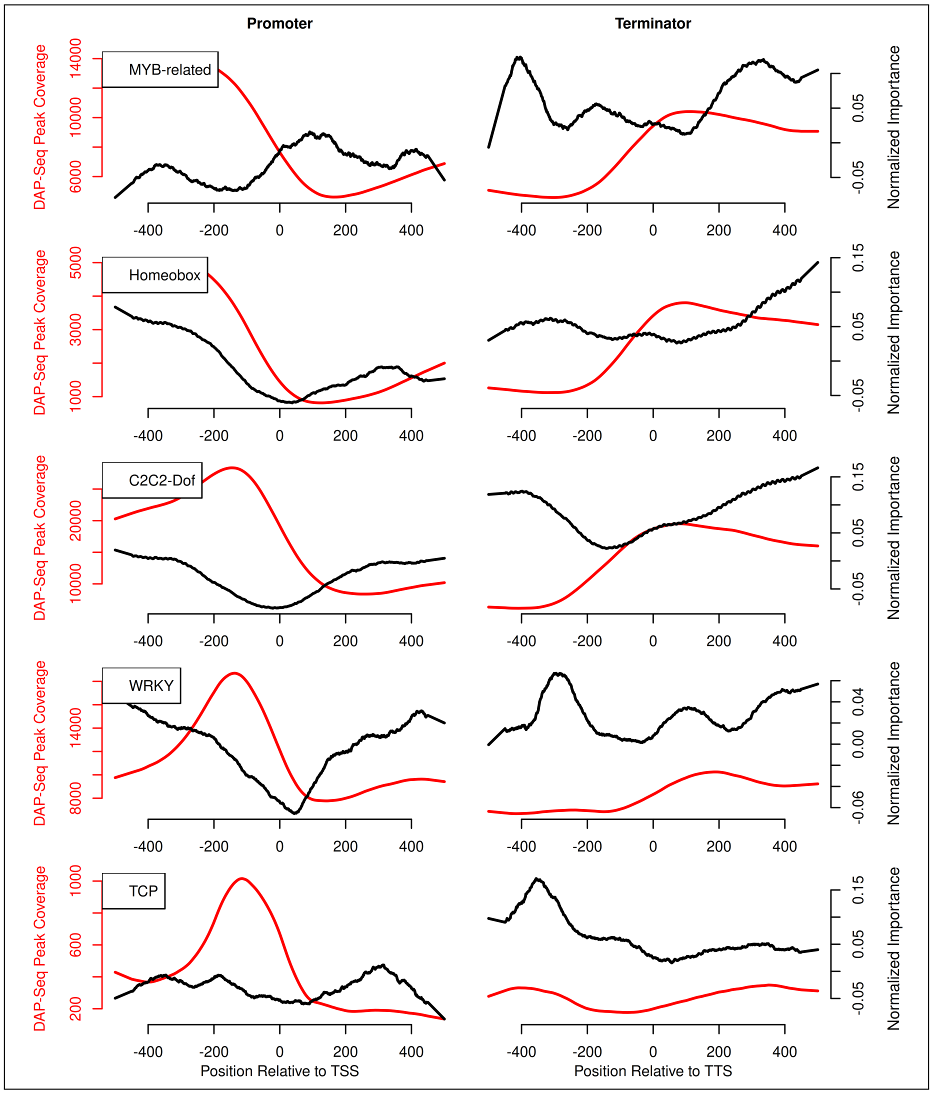

Fig. S11. Normalized importance scores from *B. napus* (black) and DAP-Seq peak coverage from *A. thaliana* (red) plotted against sequence positions $\pm 0.5kb$ around the TSS and TTS, for TFs of the families MYB-related, Homeobox, C2C2-Dof, WRKY and TCP. Values were smoothed using a 101 bp sliding window.

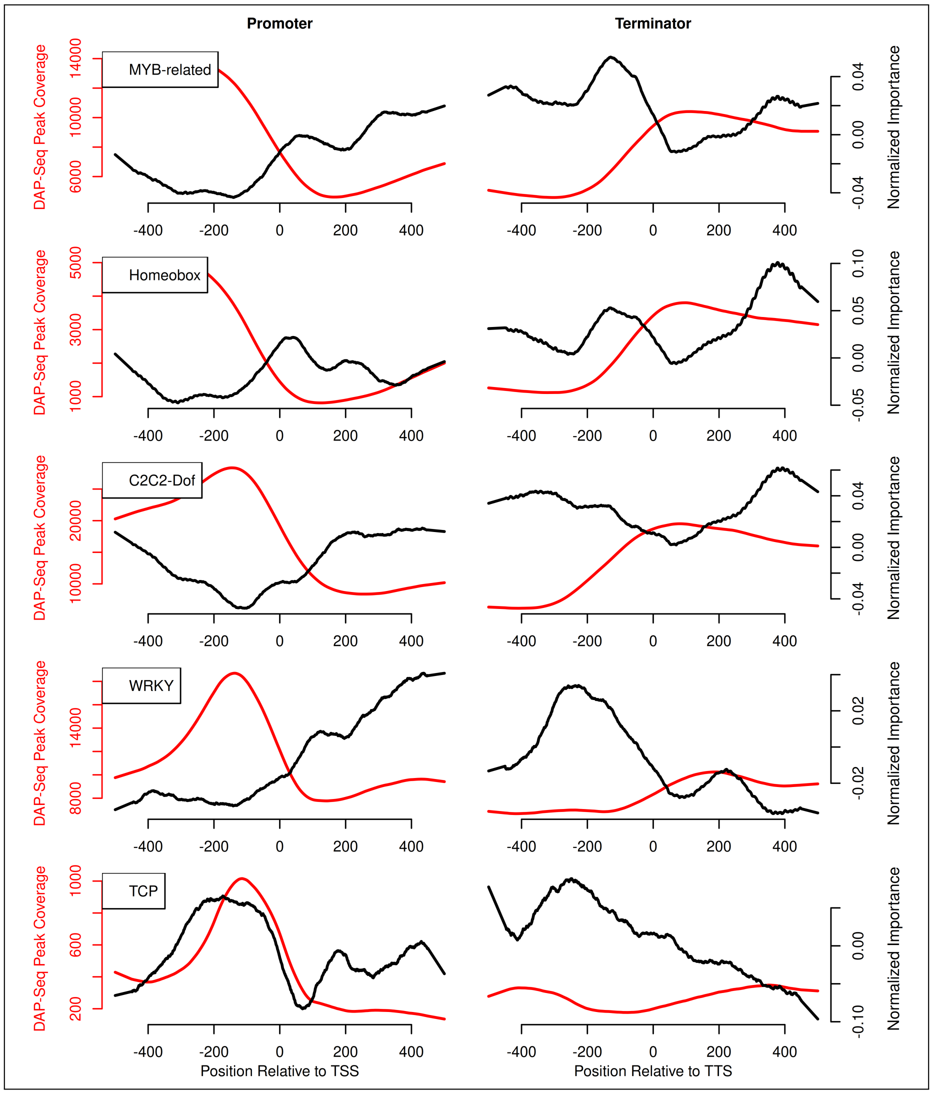

Fig. S12. Normalized importance scores from *A. thaliana* (black) and DAP-Seq peak coverage from *A. thaliana* (red) plotted against sequence positions $\pm 0.5kb$ around the TSS and TTS, for TFs of the families MYB-related, Homeobox, C2C2-Dof, WRKY and TCP. Values were smoothed using a 101 bp sliding window.

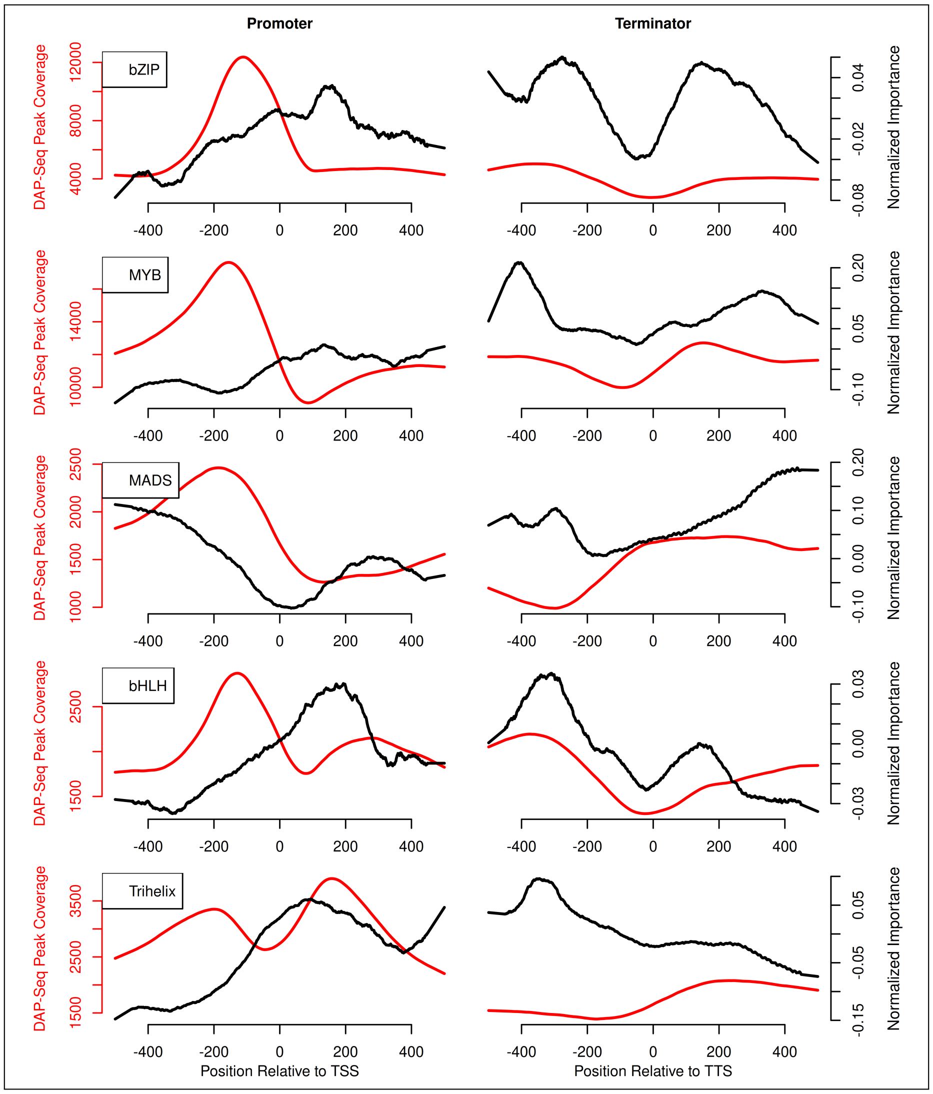

Fig. S13. Normalized importance scores from *B. napus* (black) and DAP-Seq peak coverage from *A. thaliana* (red) plotted against sequence positions $\pm 0.5kb$ around the TSS and TTS, for TFs of the families bZIP, MYB, MADS, bHLH, Trihelix. Values were smoothed using a 101 bp sliding window.

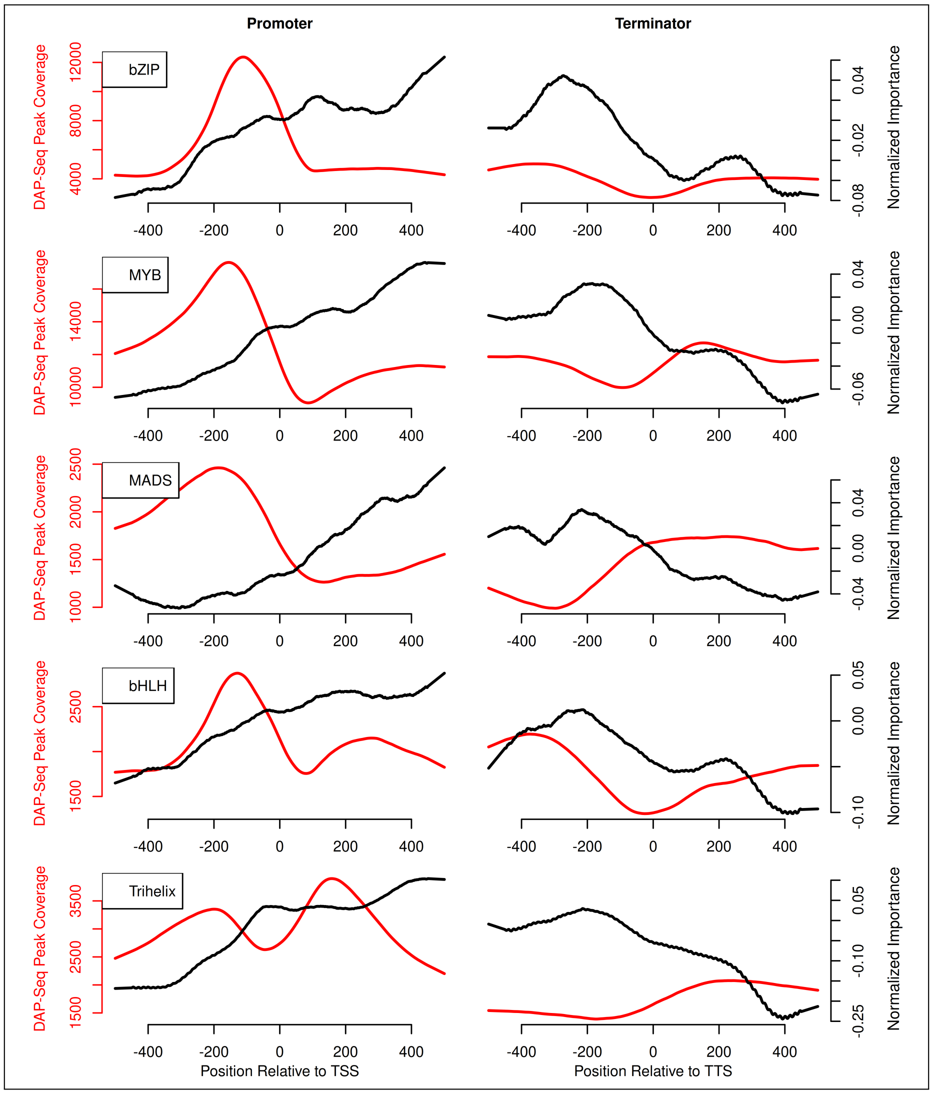

Fig. S14. Normalized importance scores from *A. thaliana* (black) and DAP-Seq peak coverage from *A. thaliana* (red) plotted against sequence positions $\pm 0.5kb$ around the TSS and TTS, for TFs of the families bZIP, MYB, MADS, bHLH, Trihelix. Values were smoothed using a 101 bp sliding window.

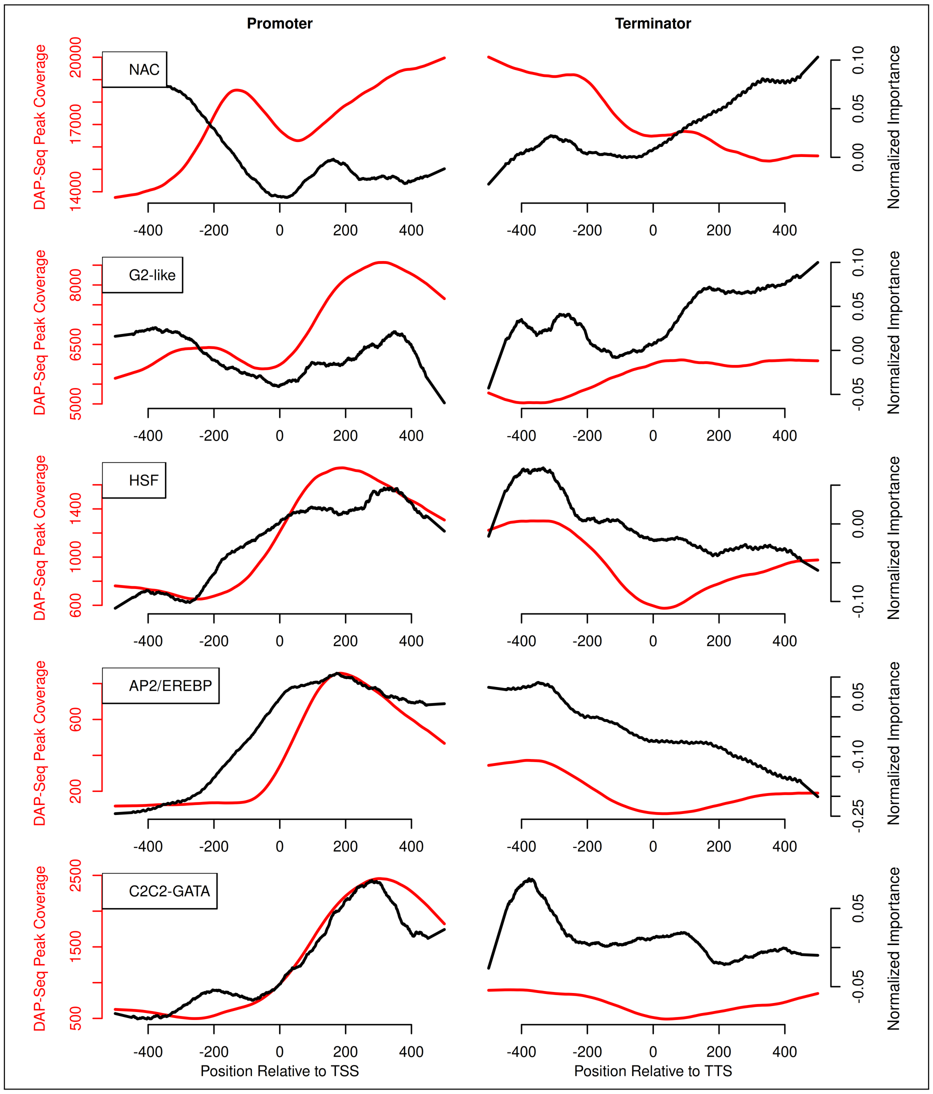

Fig. S15. Normalized importance scores from *B. napus* (black) and DAP-Seq peak coverage from *A. thaliana* (red) plotted against sequence positions $\pm 0.5kb$ around the TSS and TTS, for TFs of the families NAC, G2-like, HSF, AP2/EREBP, C2C2-GATA. Values were smoothed using a 101 bp sliding window.

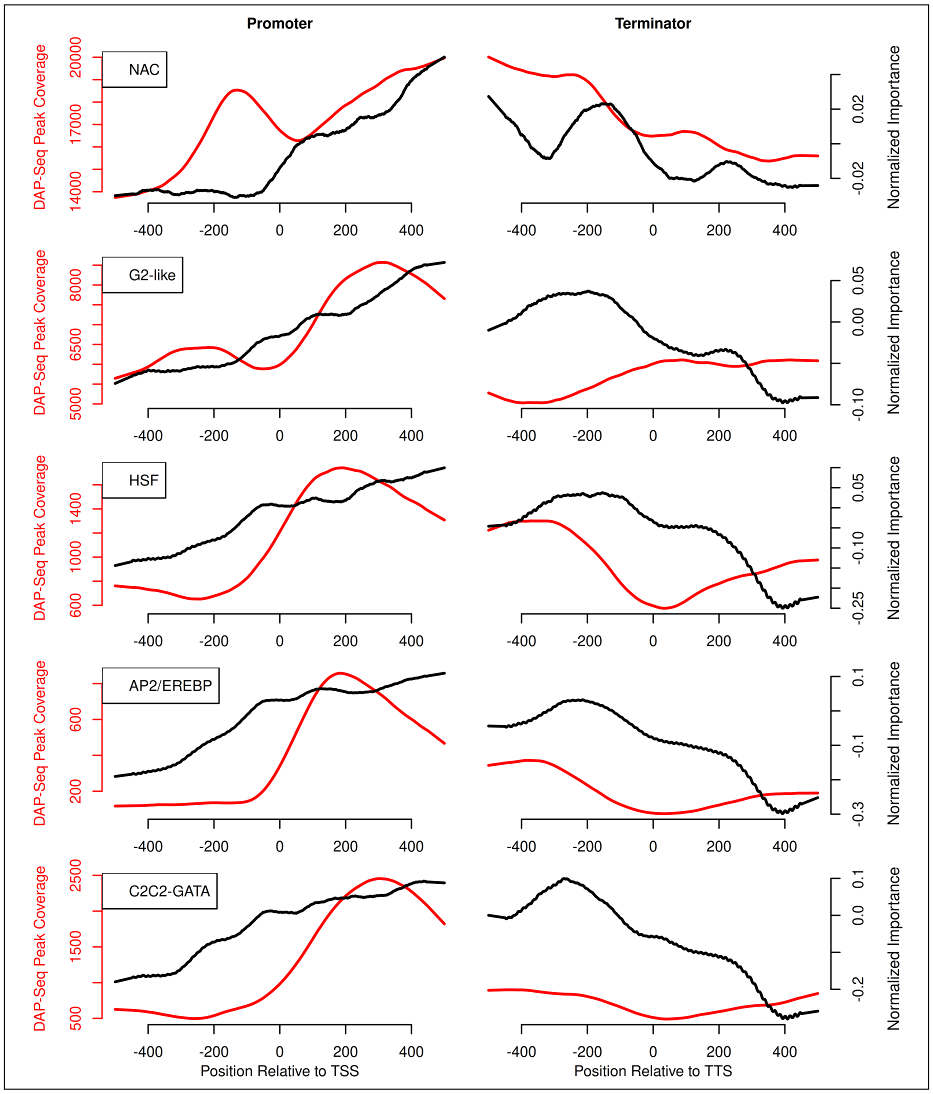

Fig. S16. Normalized importance scores from *A. thaliana* (black) and DAP-Seq peak coverage from *A. thaliana* (red) plotted against sequence positions $\pm 0.5kb$ around the TSS and TTS, for TFs of the families NAC, G2-like, HSF, AP2/EREBP, C2C2-GATA. Values were smoothed using a 101 bp sliding window.

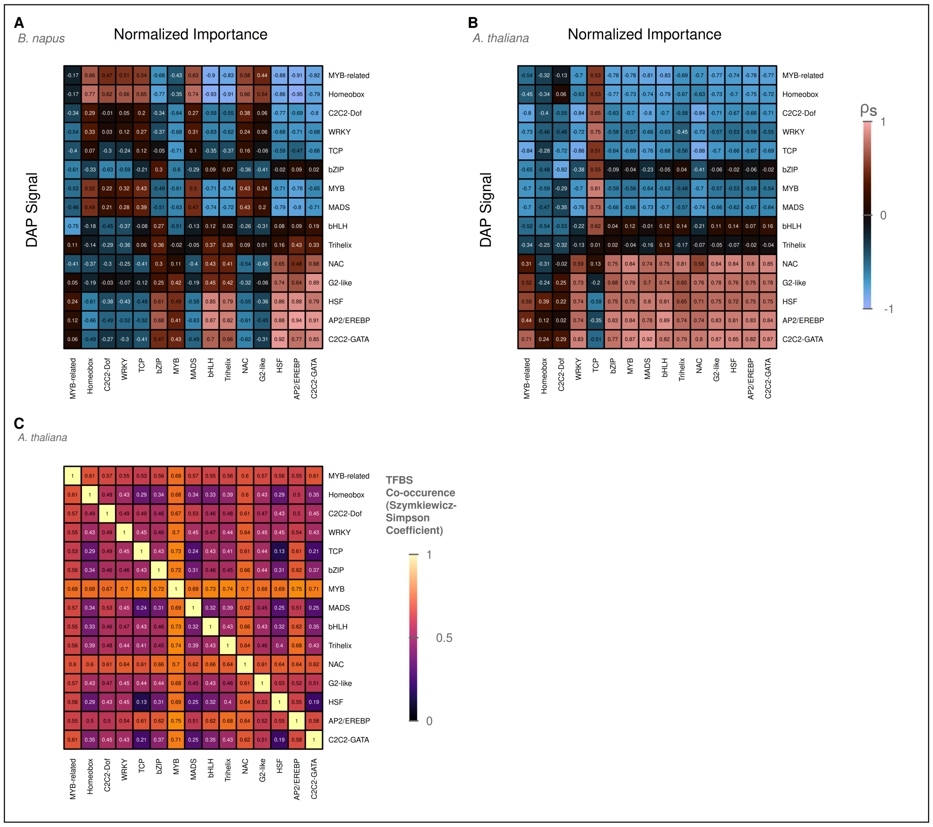

Fig. S17. TF co-occurrences and importance score correlations. (A, B) Spearman correlations between DAP-Seq signals and normalized (group-spceific) importance scores for *B. napus* (A) and *A. thaliana* (B). The interval ±500 bp around the TSS was used to calculate correlation coefficients. (C) Co-occurences of TFBSs measured by the Szymkiewicz-Simpson coefficient based on DAP-Seq peaks from different TF families overlapping the same promoter region. DAP-Seq data was only available for *A. thaliana*.
